# VCP activator reverses nuclear proteostasis defects and enhances TDP-43 aggregate clearance in multisystem proteinopathy models

**DOI:** 10.1172/JCI169039

**Published:** 2024-05-23

**Authors:** Jessica M. Phan, Benjamin C. Creekmore, Aivi T. Nguyen, Darya D. Bershadskaya, Nabil F. Darwich, Carolyn N. Mann, Edward B. Lee

**Affiliations:** Translational Neuropathology Research Laboratory, Department of Pathology and Laboratory Medicine, Perelman School of Medicine, University of Pennsylvania, Philadelphia, Pennsylvania, USA.

**Keywords:** Neuroscience, Therapeutics, Genetic diseases, Neurodegeneration, Pharmacology

## Abstract

Pathogenic variants in valosin-containing protein (*VCP*) cause multisystem proteinopathy (MSP), a disease characterized by multiple clinical phenotypes including inclusion body myopathy, Paget’s disease of the bone, and frontotemporal dementia (FTD). How such diverse phenotypes are driven by pathogenic VCP variants is not known. We found that these diseases exhibit a common pathologic feature: ubiquitinated intranuclear inclusions affecting myocytes, osteoclasts, and neurons. Moreover, knock-in cell lines harboring MSP variants show a reduction in nuclear VCP. Given that MSP is associated with neuronal intranuclear inclusions comprised of TDP-43 protein, we developed a cellular model whereby proteostatic stress results in the formation of insoluble intranuclear TDP-43 aggregates. Consistent with a loss of nuclear VCP function, cells harboring MSP variants or cells treated with VCP inhibitor exhibited decreased clearance of insoluble intranuclear TDP-43 aggregates. Moreover, we identified 4 compounds that activate VCP primarily by increasing D2 ATPase activity, where pharmacologic VCP activation appears to enhance clearance of insoluble intranuclear TDP-43 aggregate. Our findings suggest that VCP function is important for nuclear protein homeostasis, that impaired nuclear proteostasis may contribute to MSP, and that VCP activation may be a potential therapeutic by virtue of enhancing the clearance of intranuclear protein aggregates.

## Abstract 2

*VCP* variants associated with multisystem proteinopathy lead to nuclear defects in protein clearance and TDP-43 aggregation that can be reversed with VCP activators.

## Graphical abstract

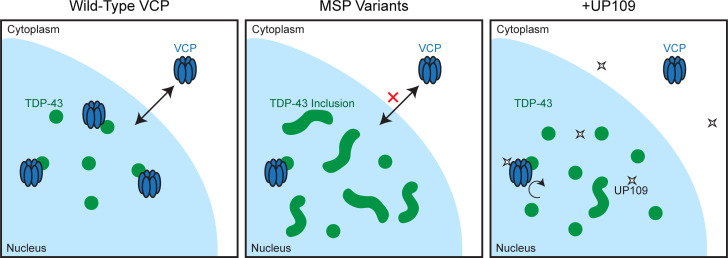

## Introduction

Inclusion body myopathy (IBM) associated with Paget disease of bone and frontotemporal dementia (IBMPFD, also known as multisystem proteinopathy, MSP) is a fatal disease that affects multiple organ systems including muscle, bone, and/or brain and causes progressive muscle weakness, defects in bone formation, and/or frontotemporal dementia (FTD) (1, 2). MSP is caused by pathogenic variants affecting valosin-containing protein (VCP), a member of the ATPases associated with diverse cellular activities (AAA+) superfamily of proteins (3, 4).

The structural conformation of VCP and the coordination of its subunits are critical for its role in substrate processing (5). VCP consists of an N-terminal domain (NTD) and 2 ATPase domains, D1 and D2. Characteristic of other AAA+ proteins, VCP forms a homohexameric structure with a central pore. VCP, together with cofactors such as UFD1 and NPLOC4 (UN), binds to polyubiquitinated substrates and unfolds ubiquitin and then the attached substrate by pulling the peptide chain through its central pore (6–8). Recently, we reported that VCP also has activity against polyubiquitinated protein aggregates (9). During this process of protein unfolding, the D1 and D2 ATPase domains of VCP seem to have distinct roles in substrate processing. The D1 ATPase domain needs to bind but not hydrolyze ATP for VCP to unfold substrate. The D2 ATPase domain, however, uses ATP hydrolysis to processively pull unfolded substrate through VCP’s central pore (8, 10).

VCP is involved in various cellular pathways including autophagy, membrane fusion, and cell cycle control, where VCP is typically involved in extracting or unfolding key protein substrates to affect downstream organelle function (11). Indeed, VCP is also critical in several protein control quality pathways (the ubiquitin proteasome system, endoplasmic-reticulum associated degradation, mitochondria-associated degradation, and ribosomal-associated degradation) in which VCP binds to polyubiquitinated substrates and utilizes mechanical energy driven by ATP hydrolysis to unfold structured substrates (6–8). Once unfolded by VCP, these substrates are then often targeted to the proteasome for degradation (12–14).

Although MSP can affect multiple different organ systems, it is unclear whether there is a common pathology across muscle, bone, and brain tissue in the diseases associated with pathogenic VCP variants (2). The neuropathology of FTD in the setting of MSP is frontotemporal lobar degeneration with TDP-43 inclusions (FTLD-TDP) type D where ubiquitinated TDP-43 inclusions are found predominantly inside nuclei of affected neurons (15, 16). Intranuclear inclusions are also a feature of inclusion body myositis (17, 18). Structurally, pathogenic VCP variants associated with MSP are located near the interface of the N-terminal and D1 ATPase domains (19). In vitro biochemistry has shown these variants are associated with increased ATPase activity and enhanced substrate processing/unfolding (19). They also affect NTD structural dynamics and cofactor binding (19). Altered VCP function can lead to a variety of cellular defects, as MSP variants have been associated with altered endoplasmic reticulum–associated degradation, autophagosome/lysosome maturation, endolysosomal processing, and mitochondrial fusion (11). We and others have shown that VCP appears to exhibit disaggregase activity against ubiquitinated inclusions, thereby reducing intracellular protein aggregation (9, 20–22). Thus, an important question is how pathogenic MSP variants in VCP, which are overactive in vitro, can lead to protein aggregation in vivo.

Mouse models expressing pathogenic MSP variants have been reported to recapitulate multiple MSP disease phenotypes including skeletal muscle abnormalities, behavioral defects, and increased expression of ubiquitinated proteins while also being associated with cytoplasmic TDP-43 accumulation (22). While pathogenic VCP variants drive MSP disease pathogenesis, relatively few studies, mainly limited to yeast, have investigated VCP’s role in nuclear protein homeostasis (23, 24). With evidence of pathological overlap of intranuclear inclusions across muscle and brain tissues, we wanted to explore VCP’s role in nuclear protein homeostasis.

In this study, we performed IHC on FTLD-TDP, IBM, and Paget’s disease of bone tissue samples and identified the presence of ubiquitinated intranuclear inclusions in all 3 diseases linked to MSP. We then generated homozygous knock-in of pathogenic VCP variants into HeLa cells and found that cells harboring A232E and R155H VCP exhibited a reduction in nuclear VCP expression. To test VCP’s effects on TDP-43 protein homeostasis in mutant cells, we modeled nuclear TDP-43 aggregation by expressing TDP-43 deficient in RNA binding (TDP-4FL) that forms soluble nuclear inclusions. We found that when the proteasome is blocked in cells expressing TDP-4FL, these intranuclear inclusions became ubiquitinated and insoluble. When TDP-4FL was expressed in cells expressing VCP-A232E and R155H variants, or VCP was inhibited with CB5083, a specific VCP inhibitor, clearance of TDP-4FL aggregates was disrupted. Additionally, we identified 4 small molecule VCP activators that stimulate VCP’s D2 ATPase activity. Moreover, when HeLa cells or iPSC-induced cortical neurons were treated with VCP activator, clearance of TDP-4FL aggregates was augmented. These results suggest that nuclear reduction of VCP expression associated with pathogenic MSP variants results in a loss of nuclear protein homeostasis and reduced clearance of intranuclear TDP-43 aggregates, and that this phenotype may be rescued by a small molecule VCP activator.

## Results

### Ubiquitinated intranuclear inclusions in FTLD-TDP type D, IBM, and Paget’s disease of bone.

**Figure 1:**
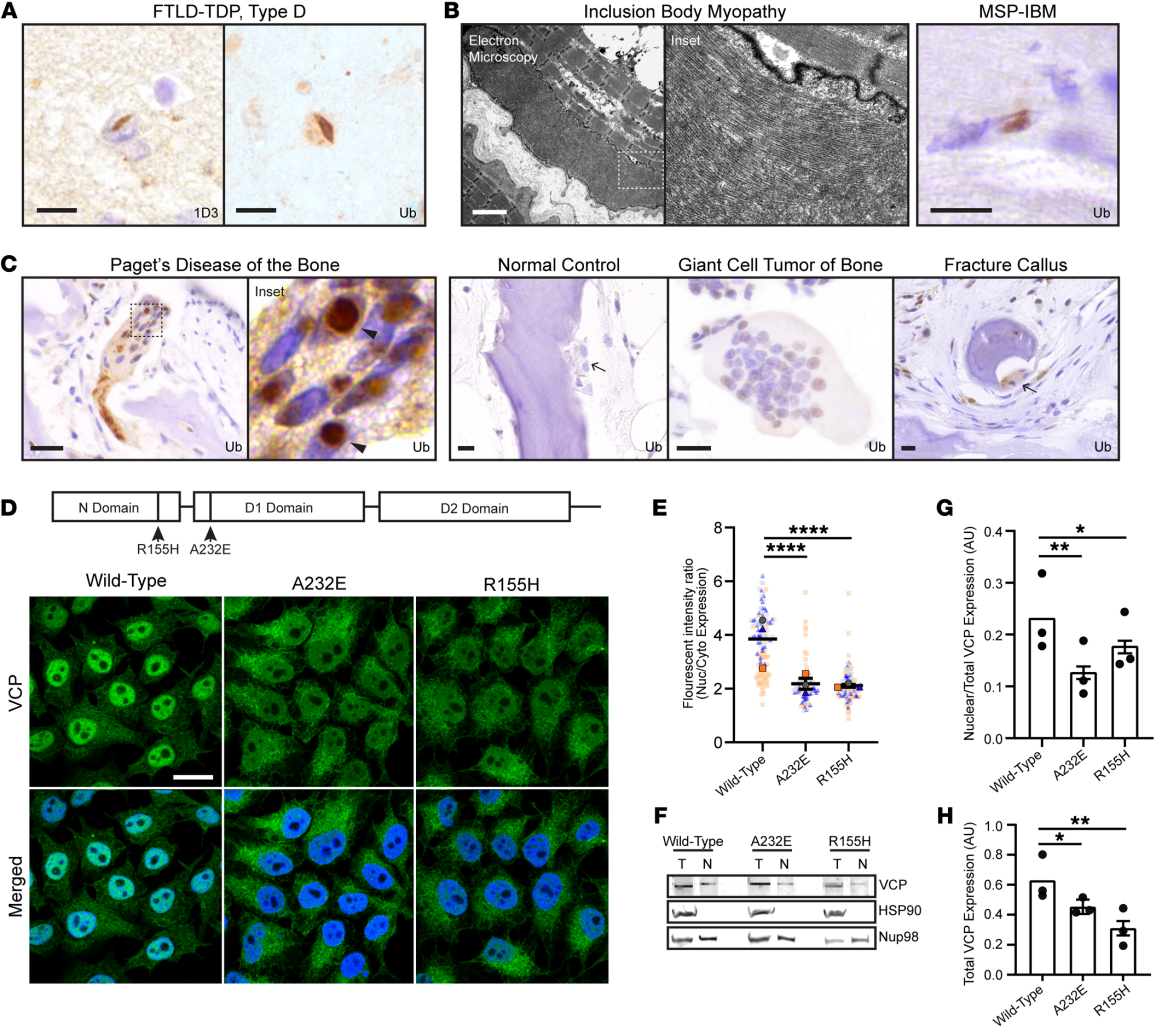
Ubiquitinated intranuclear inclusions in FTLD-TDP, IBM, and Paget’s disease of bone, and reduced nuclear VCP localization in CRISPR-edited cells harboring pathogenic VCP variants. (**A)** IHC stains of FTLD-TDP type D neocortical brain tissue for phosphorylated TDP-43 (1D3) or ubiquitin. Scale bar: 10 μm. (**B**) Electron microscopy and IHC stain for ubiquitin of IBM muscle. Scale bars: electron microscopy 2 μm; IHC 10 μm. (**C**) IHC stains for ubiquitin of Paget’s disease of bone, normal control bone, giant cell tumor of the bone, or fracture callus. Arrowheads point to ubiquitin-positive intranuclear inclusions and arrows point to osteoclasts without intranuclear inclusions. Scale bars: 10 μm. (**D**) Immunofluorescence staining for VCP (green) and DAPI (blue) of WT HeLa cell lines versus CRISPR/Cas9 knock-in HeLa cell lines harboring A232E or R155H VCP pathogenic variants. Scale bar: 15 μm. (**E**) Ratio of nuclear-to-cytoplasmic VCP immunofluorescence intensity (*n* = 325 cells across 3 cell lines from 3 independent experiments denoted with blue triangles, gray circles, or orange squares. Solid shapes represent mean average of each biological replicate and transparent shapes represent individual data points; data shown as individual data points with overall beta ± SE. LME, **** *P* < 0.0001). (**F**) Immunoblot of total (T) or nuclear (N) VCP, HSP90 (cytoplasmic marker), Nup98 (nuclear marker) protein in WT, A232E, or R155H cell lines. (**G**) Quantification of nuclear to total VCP protein expression of A232E or R155H normalized to WT. (**H**) Quantification of total VCP protein expression of A232E or R155H normalized to WT (For **G** and **H**: *n* = 3 experiments; results are expressed as β ± SEM; LME, *P < 0.05, **P < 0.01, ****P ≤ 0.0001).

Given the diverse tissues that are affected in MSP, we sought to identify whether there is a common pathology across FTLD-TDP type D, IBM, and Paget’s disease of bone. In individuals with FTD, pathogenic VCP variants are known to be associated with ubiquitinated neuronal intranuclear inclusions comprised of TDP-43 (representative examples shown in Figure 1A) (16, 25). Similarly, IBM is known to be associated with ubiquitin-positive intranuclear inclusions of an unknown protein, as shown by electron microscopy and IHC (Figure 1B) (17, 26, 27). It has not been determined whether Paget’s disease of bone, a disease associated with overactive osteoclasts, also exhibits ubiquitinated intranuclear inclusions. Ten bone biopsies from cases of Paget’s disease of bone were immunostained for ubiquitin, of which 2 biopsies were excluded due to scant tissue with rare to absent osteoclasts. The remaining 8 cases showed osteoclasts with strongly ubiquitin-positive inclusions including conspicuous, spherical intranuclear inclusions together with a few granular, apparently cytoplasmic aggregates (Figure 1C). Osteoclasts from normal bone (*n* = 10) or other osteoclast-rich lesions including giant cell tumor of bone (*n* = 10) and fracture calluses (*n* = 10) did not exhibit intranuclear inclusions. Our findings are consistent with prior electron microscopy studies indicating that osteoclasts in Paget’s disease of bone exhibit abnormal intranuclear inclusions (18) and suggests that these 3 disparate diseases share a common pathology.

### Nuclear VCP expression is reduced in HeLa cells expressing A232E and R155H variants.

The presence of ubiquitin-positive intranuclear inclusions in FTLD-TDP type D, IBM, and Paget’s disease of bone suggested that these diseases are associated with a loss of nuclear proteostasis. Prior overexpression studies have suggested that pathogenic VCP variants are partially excluded from the nucleus (28, 29). To determine whether pathogenic VCP variants result in changes in the subcellular localization of endogenous VCP, CRISPR/Cas9 was used to edit the endogenous *VCP* locus by knocking in 2 different pathogenic *VCP* variants into HeLa cells: p.A232E (the variant associated with the highest ATPase activity in vitro) and p.R155H (the most common pathogenic MSP variant). Targeted sequencing of knock-in cell lines at the top predicted off-target genomic sites failed to reveal off-target mutations (Supplemental Table 1; supplemental material available online with this article; https://doi.org/10.1172/JCI169039DS1). Mutant cell lines were immunostained for VCP and compared with parental HeLa cells, which revealed that VCP is localized to both the cytoplasm and nucleus. However, A232E or R155H knock-in cell lines showed a reduction in the relative amount of nuclear VCP (Figure 1D). Quantitative image analysis showed that there is an approximately 60% reduction in the fluorescence intensity ratio of nuclear to cytoplasmic VCP signal in A232E and R155H cells compared with WT cells (*P* < 0.0001; Figure 1E). These findings were reconfirmed with an immunoblot of nuclear and total VCP protein expression (Figure 1F). The ratio of nuclear-to-total VCP expression showed a 30%–50% reduction in VCP expression compared with WT (*P* < 0.01 for A232E and P< 0.05 for R155H with or without Bonferonni correction; Figure 1G). Overall VCP expression of in VCP-A232E and VCP-R155H cells was also significantly reduced by 30%–50% compared with WT (*P* < 0.05 for A232E and *P* < 0.01 for R155H with or without Bonferonni correction; Figure 1H).

Given the overall decreased VCP expression, we assessed whether there might be global defects in proteostasis in VCP-A232E and VCP-R155H cells. At baseline, VCP-A232E and VCP-R155H cells exhibited strongly ubiquitin-positive intranuclear puncta that were not observed in WT cells (Supplemental Figure 1A). Additionally, proteasome inhibition led to an increase of overall ubiquitination in VCP-A232E and VCP-R155H cells compared with WT cells, particularly within the nucleus (Supplemental Figure 1A). These findings were confirmed biochemically with sequential extraction of soluble and insoluble proteins that were immunoblotted for ubiquitin (Supplemental Figure 1B). Proteasome inhibition led to an increase of ubiquitinated insoluble proteins in VCP-A232E and VCP-R155H cells compared with WT cells (*P* < 0.05 for insoluble protein; Supplemental Figure 1, B–D). These results indicate that overall protein homeostasis is disrupted in the VCP-A232E and R155H cell lines.

### Proteasome inhibition promotes the formation of insoluble ubiquitinated intranuclear TDP-43 inclusions.

While pathogenic VCP variants have been linked to a variety of cytoplasmic defects, we hypothesized that the reduced nuclear localization of mutant VCP would lead to diminished nuclear proteostasis. To better study VCP’s role in nuclear protein homeostasis, we focused our studies on TDP-43 since it is known to be the protein that forms inclusions in FTLD-TDP type D. We expressed myc-tagged TDP-43, which showed diffuse nuclear expression in WT cells (Supplemental Figure 2A). To promote the formation of intranuclear TDP-43 inclusions, cells were treated with MG132 to inhibit the proteasome, which resulted in the formation of coarse nuclear puncta, as demonstrated by immunofluorescence (Supplemental Figure 2A). Moreover, these nuclear aggregates were positive for both ubiquitin and VCP (Supplemental Figure 2, E and F). These morphologic changes were associated with an increase in insoluble TDP-43 protein over time, as determine by immunoblotting (*P* < 0.05; Supplemental Figure 2, B–D).

**Figure 2:**
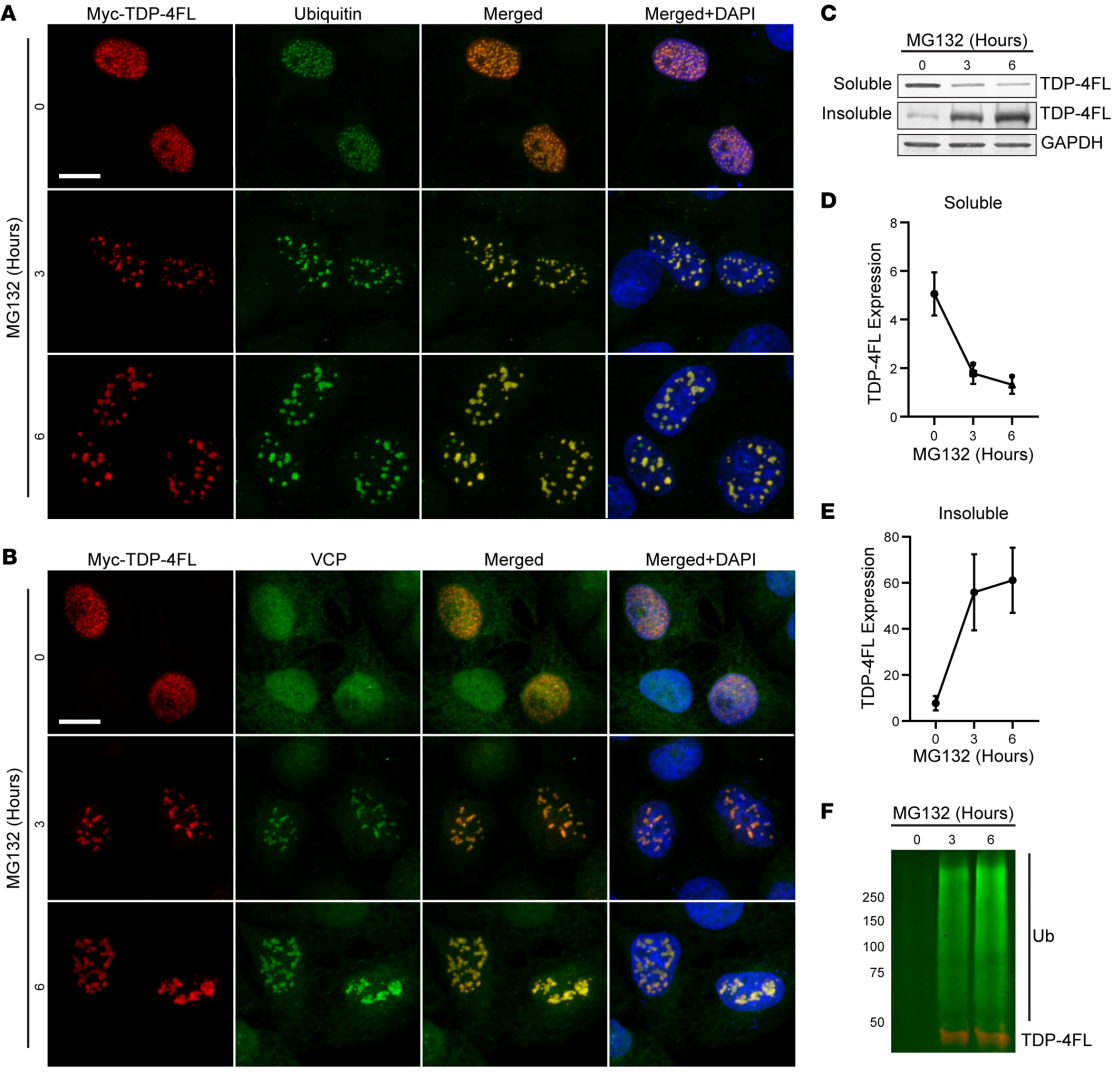
Proteasome inhibition promotes insoluble intranuclear TDP-4FL inclusions that colocalize with ubiquitin and VCP. (**A** and **B**) Immunofluorescence confocal images of HeLa cells expressing TDP-4FL treated with MG132 for 0, 3, or 6 hours stained for myc (TDP-4FL, red) and either ubiquitin (**A**, green) or VCP (**B**, green). Scale bar: 10 μm. (**C**) Immunoblots for myc (TDP-4FL) from soluble and insoluble protein fractions from HeLa cells expressing TDP-4FL treated with MG132. GAPDH provided as a loading control. (**D** and **E)** Quantification of (**D**) soluble or (**E**) insoluble TDP-4FL immunoblots. (*n* = 4, results are expressed as mean ± SEM. 1-way ANOVA, **P < 0.01 for soluble protein, *P < 0.05 for insoluble protein). (**F**) Anti-myc immunoprecipitation from insoluble protein fractions, immunoblotted for myc (TDP-4FL, red) and ubiquitin (green).

RNA binding has been demonstrated to alter TDP-43 dynamics, promoting self-association into phase-separated puncta called anisosomes (30–33). We therefore tested whether an RNA-binding–deficient TDP-43 mutant containing phenylalanine-to-leucine mutations within RNA recognition motifs 1 and 2 (F147/149/229/231L, hereafter referred to as TDP-4FL) would result in an even more robust model of intranuclear TDP-43 inclusions (31). Expression of TDP-4FL led to the formation of small, intranuclear, phase-separated anisosomes, as reported previously (32, 33) Supplemental Figure 3, A and B). Moreover, when the proteasome was inhibited with MG132 in cells expressing TDP-4FL, the size, morphology, and solubility of TDP-4FL protein inclusions were altered. At 3 or 6 hours of MG132 treatment, the small anisosome structures coalesced into larger, irregularly shaped aggregates. To confirm that this is not an off-target effect of MG132, we treated HeLa cells expressing TDP-4FL with 2 other proteasome inhibitors, ixazomib and epoxomicin, and found that these compounds affect TDP-4FL similarly, generating larger TDP-4FL inclusions upon proteasome inhibition (Supplemental Figure 4). Confocal double immunofluorescence images of MG132-treated cells revealed that these TDP-4FL aggregates colocalize with ubiquitin (using an antibody that recognizes K29-, K48-, and K63-linked polyubiquitin and monoubiquitin) (Figure 2A) and VCP (Figure 2B). To test the solubility of the TDP-4FL aggregates, sequential biochemical extraction of cells were used to separate RIPA-soluble proteins versus RIPA insoluble (urea soluble) proteins. MG132 treatment over time resulted in a decrease in soluble TDP-4FL (Figure 2, C and D) with a concomitant increase in insoluble TDP-4FL (*P* < 0.01 for soluble protein; *P* < 0.05 for insoluble protein; Figure 2, C and E). Immunoprecipitation of insoluble myc-TDP-4FL also revealed that MG132 treatment resulted in ubiquitination of TDP-4FL protein (Figure 2F).

### VCP variants lead to a defect in nuclear protein homeostasis.

**Figure 3:**
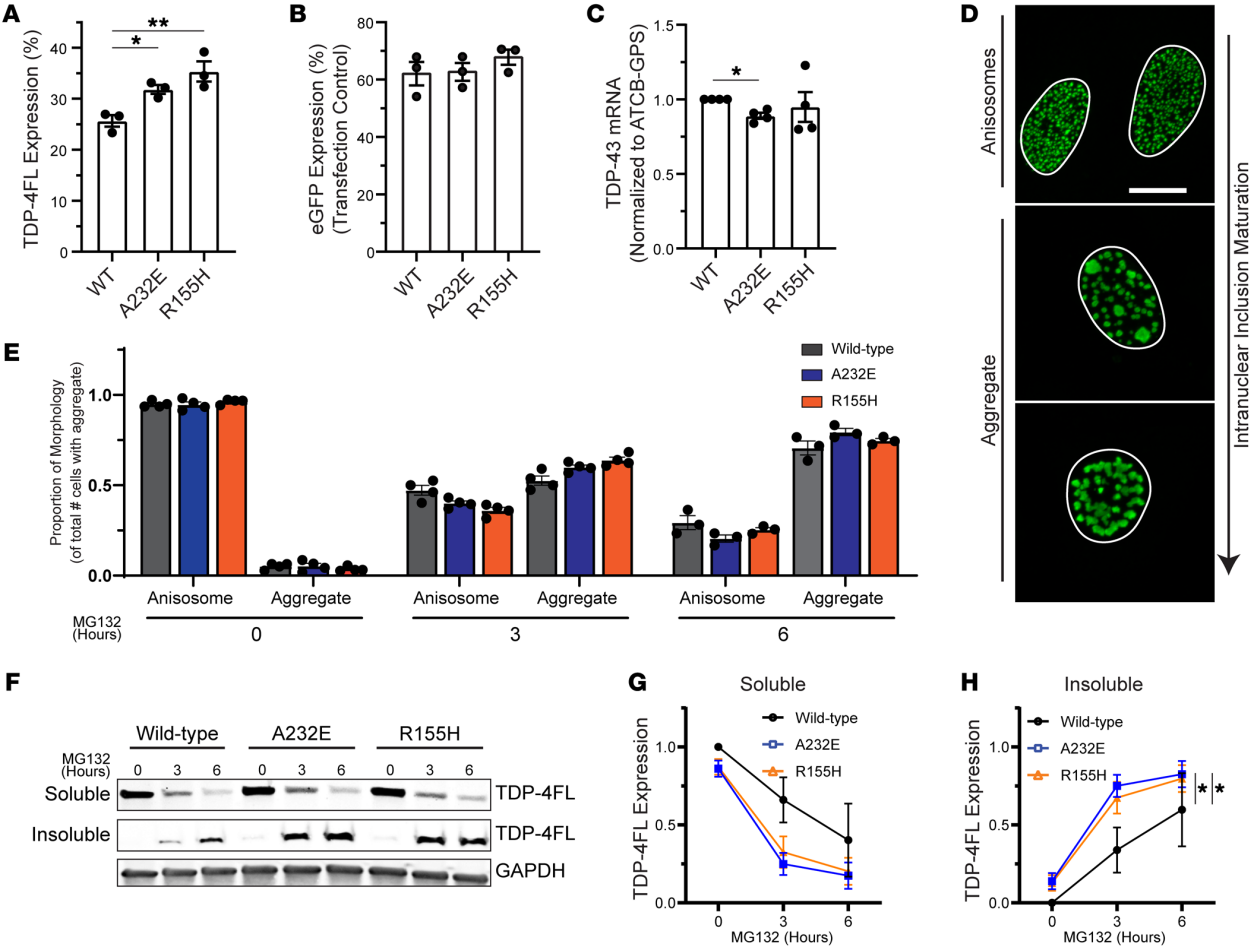
TDP-4FL anisosomes become larger and more insoluble due to pathogenic A232E and R155H VCP variants. (**A** and **B)** Analysis of immunofluorescence images to determine the percentage of cells expressing (**A**) TDP-4FL or (**B**) eGFP as a percentage of total cell counts after 24 hours of transient TDP-4FL or eGFP transfection in WT HeLa cells or knock-in HeLa cells harboring A232E and R155H VCP variants. (Results are expressed as mean percentage values from each experiment and overall mean ± SEM. TDP-4FL, *n* = 6,390 cells counted across 3 cell lines over 3 independent experiments; 1-way ANOVA, **P < 0.01; Dunnett’s multiple comparison post hoc, *P < 0.05, **P < 0.01. eGFP, *n* = 2,135 cells counted across 3 cell lines over 3 independent experiments; 1-way ANOVA, *P* = 0.459) (**C**) Quantification of TDP-43 mRNA levels by qPCR (*n* = 4, results expressed as mean ± SEM; 1 sample *t* test: *P < 0.05). (**D**) Representative confocal images of immunofluorescence for TDP-4FL with no MG132 treatment or MG132 for 6 hours. Nuclear structures were categorized as either anisosomes or aggregates based on morphology. Scale bar: 10 μm. (**E**) Analysis of immunofluorescence images to determine the proportion of anisosome versus aggregate morphology in WT, A232E, or R155H cells treated with MG132 (*n* = 7,058 cells counted across 3 cell lines over 3 independent experiments; results are expressed as mean proportion values from each experiment and overall β ± SE, LME for A232E cells: time *P* < 0.0001, genotype *P* = 0.708, time × genotype **P < 0.01; LME for R155H cells: time *P* < 0.0001, genotype *P* = 0. 734, time × genotype *P < 0.05). (**F**) Immunoblots of soluble and insoluble TDP-FL protein (mouse anti-myc antibody) in WT, A232E, and R155H cells treated with MG132. GAPDH shown as a loading control. (**G** and **H)** Quantification of immunoblots for (**G**) soluble and (**H**) insoluble TDP-FL protein (normalized to soluble + insoluble TDP-FL protein levels within genotype) in WT, A232E, and R155H cells treated with MG132. (*n* = 3, results expressed as mean ± SEM; 2-way ANOVA for soluble protein: time *P < 0.05, genotype *P < 0.05, time × genotype *P* = 0.3613; for insoluble protein: time *P < 0.05, genotype *P < 0.05, time × genotype *P* = 0.3613; Dunnett’s post hoc analysis: *P < 0.05).

To assess VCP’s effects on TDP-4FL protein homeostasis in mutant cells, we overexpressed TDP-4FL in our VCP-A232E and R155H cell lines. Interestingly, a higher proportion of A232E and R155H cells expressed TDP-4FL compared with WT cells (*P* < 0.01; Dunnett’s multiple comparison post hoc *P* < 0.05, *P* < 0.01; Figure 3A). This result was not due to altered transfection efficiency, as there was no difference in the proportion of cells expressing a control GFP construct, suggesting that increased TDP-4FL expression in the mutant cells may be driven by defects in VCP function (Figure 3B). qPCR also demonstrated that TARDBP mRNA was not increased in mutant cells. Rather, TARDBP mRNA was mildly decreased in VCP-A232E but not in VCP-R155H cells compared with WT cells (Figure 3C). These results suggest that increased TDP-4FL seen at the protein level was not due to differences in mRNA expression, but rather may be due to altered protein turnover. Importantly, higher TDP-4FL expression may have effects on the propensity for downstream aggregate formation.

Given that proteasome inhibition resulted in a change in the morphology of TDP-4FL inclusions from small anisosome to larger insoluble aggregate (Figure 3D), we assessed whether TDP-4FL inclusion morphology was altered in WT, VCP-A232E, and VCP-R155H cell lines with MG132 treatment over time. To test this, we categorized TDP-4FL positive cells across all 3 cell lines in a blinded manner as having either small anisosomes or larger aggregates (Supplemental Figure 3C). In all cell lines, there was a reduction in anisosome morphology with an increase in aggregate morphology upon treatment with MG132 over time. Moreover, there was also a small but statistically significant difference between cell lines, with both A232E and R155H cells exhibiting a higher proportion of cells with larger aggregate morphology compared with WT cells (A232E genotype x time *P* < 0.01; R155H genotype x time *P* < 0.05; Figure 3E).

TDP-4FL solubility was then assessed across cell lines to determine whether biochemical analysis provided a more robust readout of intranuclear inclusion insolubility. Cells were extracted with RIPA buffer followed by a more stringent urea buffer to obtain soluble versus insoluble protein fractions. WT, A232E, and R155H cells exhibited a similar decrease in soluble TDP-4FL protein levels with MG132 treatment over time (time *P* < 0.01, genotype *P* = 0.690, time x genotype *P* = 0.513; Figure 3, F and G, and Supplemental Figure 5A). However, at both 3 and 6 hours of MG132 treatment, insoluble TDP-4FL protein levels were significantly higher in A232E and R155H cells compared with WT cells (time *P* < 0.01, genotype *P* < 0.05, time x genotype *P* = 0.210; Dunnett’s post hoc analysis *P* < 0.05; Figure 3, F and H).

### Clearance of insoluble intranuclear TDP-4FL aggregates is diminished by VCP inhibitor CB5083.

**Figure 4:**
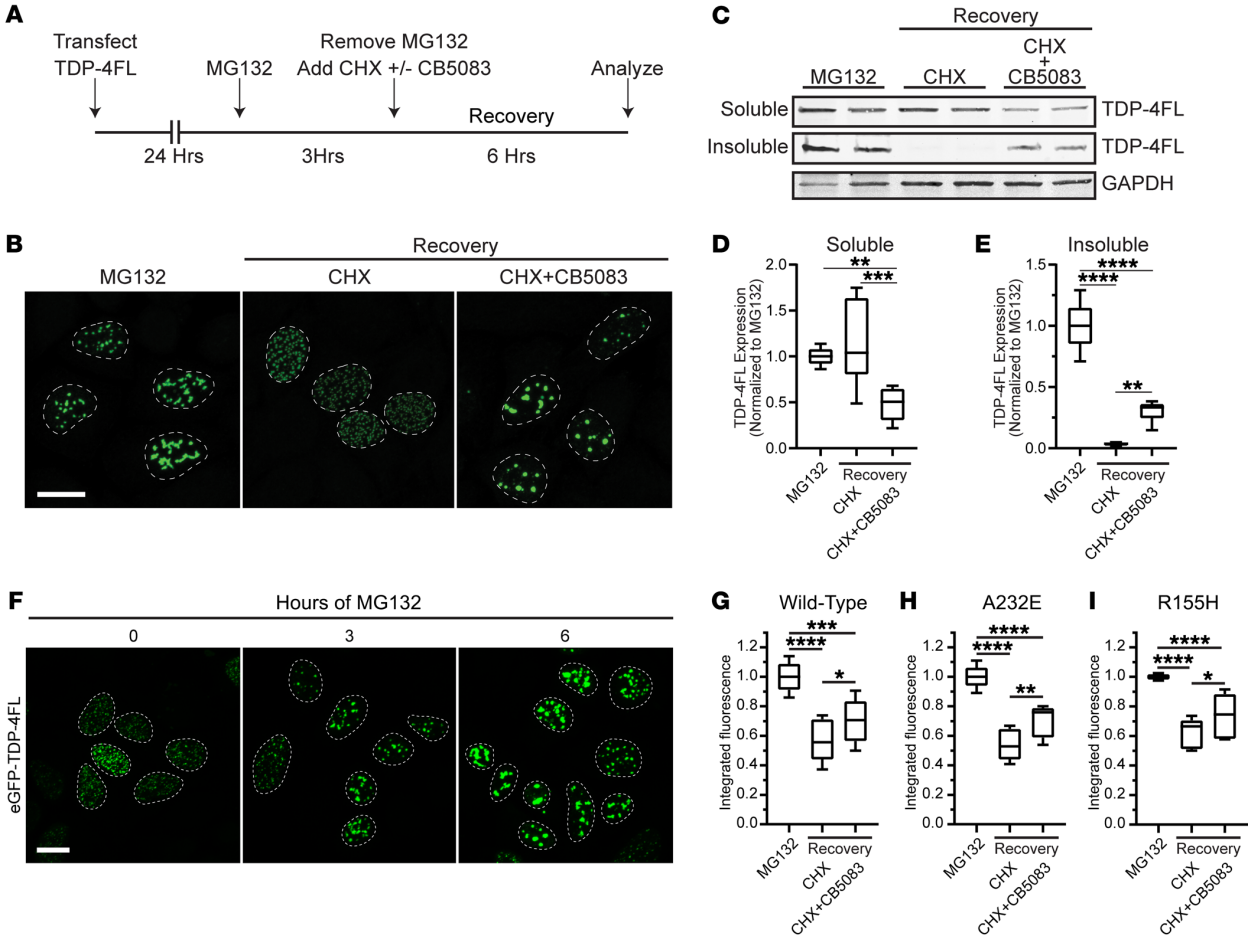
VCP inhibition impairs turnover of insoluble intranuclear TDP-4FL inclusions. (**A**) Schematic of transfection and drug treatment timing to examine the turnover of intranuclear TDP-4FL inclusions. (**B**) Representative confocal images of immunofluorescence for TDP-4FL in HeLa cells treated with MG132 (left) versus cells treated with MG132 followed by CHX (middle) or cells treated with MG132 followed by CHX + CB5083 (right). Scale bar: 10 μm. (**C**) Immunoblots for TDP-4FL of soluble and insoluble protein fractions from HeLa cells treated with MG132 versus HeLa cells that recovered with CHX alone or CHX + CB5083. GAPDH shown as a loading control. (**D**) Quantification of soluble TDP-4FL immunoblots (*n* = 3 with 2 technical replicates per experiment; data shown as box-and-whisker plot; LME, drug treatment *P* < 0.0001; Tukey’s post hoc analysis, **P < 0.01, ***P < 0.001). (**E**) Quantification of insoluble TDP-4FL immunoblots (*n* = 3 with 2 technical replicates per experiment; data shown as box-and-whisker plot; LME, drug treatment *P* < 0.0001; Tukey’s post hoc analysis, **P < 0.01, ****P < 0.0001). (**F**) Representative confocal images of HeLa cells expressing eGFP-TDP4FL treated with MG132. Scale bar: 10 μm. (**G**) WT HeLa cells or CRISPR/Cas9 knock-in HeLa cells harboring (**H**) A232E and (**I**) R155H VCP pathogenic variants. Cells expressing GFP-TDP-4FL were treated with MG132 versus cells treated with MG132 followed by CHX or cells treated with MG132 followed by CHX + CB5083 and analyzed by flow cytometry (*n* = 4 experiments each with 2 technical replicates per condition, data shown as box-and-whisker plot; LME drug treatment *P* < 0.0001; Tukey’s post-hoc analysis, *P < 0.05, **P < 0.01, ***P < 0.001, ****P < 0.0001).

To examine the turnover of intranuclear TDP-4FL aggregates in a more dynamic manner, HeLa cells expressing TDP-4FL were treated with MG132 for 3 hours to induce formation of insoluble intranuclear aggregates followed by removal of MG132 and addition of the translational inhibitor cycloheximide (CHX) for 6 hours. This CHX recovery period allowed cells to begin clearing intranuclear TDP-4FL aggregates in the absence of any potential effect of new protein synthesis, using sequential biochemical fractionation followed by immunoblotting of TDP-4FL from soluble and insoluble protein fractions as a measure of intranuclear aggregate clearance (Figure 4A). CHX recovery was done with or without the specific VCP inhibitor, CB5083, to determine whether VCP activity was involved in intranuclear aggregate clearance (Figure 4A). Cells treated with 3 hours of MG132 exhibited an accumulation of larger, irregularly shaped, intranuclear TDP-4FL aggregates. Upon recovery in the presence of CHX, intranuclear aggregates were resolved with cells exhibiting small TDP-4FL anisosomes. However, cotreatment with both CHX and CB5083 resulted in residual irregularly shaped intranuclear TDP-4FL aggregates (Figure 4B). Pretreatment of cells with MG132 prior to CB5083 treatment was necessary for the accumulation of insoluble TDP-4FL aggregates because treatment of CB5083 alone had no effect on TDP-4FL aggregate formation (Supplemental Figure 5B).

To provide biochemical evidence that VCP inhibition reduces intranuclear aggregate clearance, soluble and insoluble protein fractions were immunoblotted for TDP-4FL. While the CHX recovery resulted in a small increase in soluble TDP-4FL levels, the presence of CB5083 during the CHX recovery period was associated with a significant decrease in soluble TDP-4FL protein (*P* < 0.0001; Tukey’s post hoc analysis *P* < 0.01, *P* < 0.001; Figure 4, C and D). Even more evident was the near complete absence of insoluble TDP-4FL levels after the CHX recovery period. In contrast, the addition of CB5083 during the CHX recovery period resulted residual amounts of insoluble TDP-4FL protein, consistent with our hypothesis that VCP helps clear insoluble intranuclear TDP-4FL aggregates (*P* < 0.0001; Tukey’s post hoc analysis *P* < 0.01, *P* < 0.0001; Figure 4, C and E).

We sought to recapitulate these findings using a quantitative live cell approach. Cells were transfected with a TDP-4FL construct containing a GFP tag, and flow cytometry was used to determine integrated fluorescence intensity (percent GFP multiplied by median GFP fluorescence) as a cumulative measurement of TDP-4FL protein levels where higher integrated fluorescence intensity was used as a measure of decreased clearance of intranuclear TDP-4FL aggregates. To confirm that GFP-TDP-4FL protein responded to proteasome inhibition similarly to what was observed above using non-GFP–tagged TDP-4FL protein, cells expressing GFP-TDP-4FL were treated with MG132 for 0, 3, or 6 hours. Confocal immunofluorescence revealed that similar to myc-TDP-4FL, GFP-TDP-4FL formed intranuclear anisosome inclusions, and longer MG132 treatment led to an accumulation of larger GFP-TDP-4FL aggregates (Figure 4F). Thus, to study clearance and turnover of intranuclear GFP-TDP-4FL, integrated fluorescence intensity was measured using flow cytometry using the same experimental paradigm shown in Figure 4A. HeLa cells expressing GFP-TDP-4FL were first treated with MG132 for 3 hours to build up insoluble GFP-TDP-4FL aggregates followed by MG132 removal and recovery in CHX with or without CB5083 for 6 hours. In WT cells, recovery with CHX alone led to a decrease in integrated fluorescence intensity, consistent with the clearance of GFP-TDP-4FL aggregates upon removal of MG132. In contrast, with the presence of both CHX and CB5083 during recovery, integrated fluorescence intensity was significantly higher than CHX-only treatment, indicating that CB5083 inhibited the clearance of GFP-TDP-4FL aggregates (*P* < 0.0001; Tukey’s post hoc analysis *P* < 0.05, *P* < 0.001, *P* < 0.0001; Figure 4G). These results recapitulated our biochemical findings demonstrating that CB5083 treatment leads to decreased clearance of insoluble TDP-4FL aggregates (Figure 4, C and E) and confirm that VCP is involved in GFP-TDP-4FL clearance. In addition, when using CRISPR-edited cells expressing VCP-A232E and R155H, CB5083 also led to a greater integrated fluorescence intensity compared with CHX-only treatment (*P* < 0.0001; Tukey’s post hoc analysis *P* < 0.05, *P* < 0.01, *P* < 0.0001; Figure 4, H and I). These findings indicate that, in the setting of pathogenic VCP variants, pharmacologic VCP inhibition inhibits the turnover of intranuclear GFP-TDP-4FL aggregates.

### Small molecule VCP activators.

**Figure 5:**
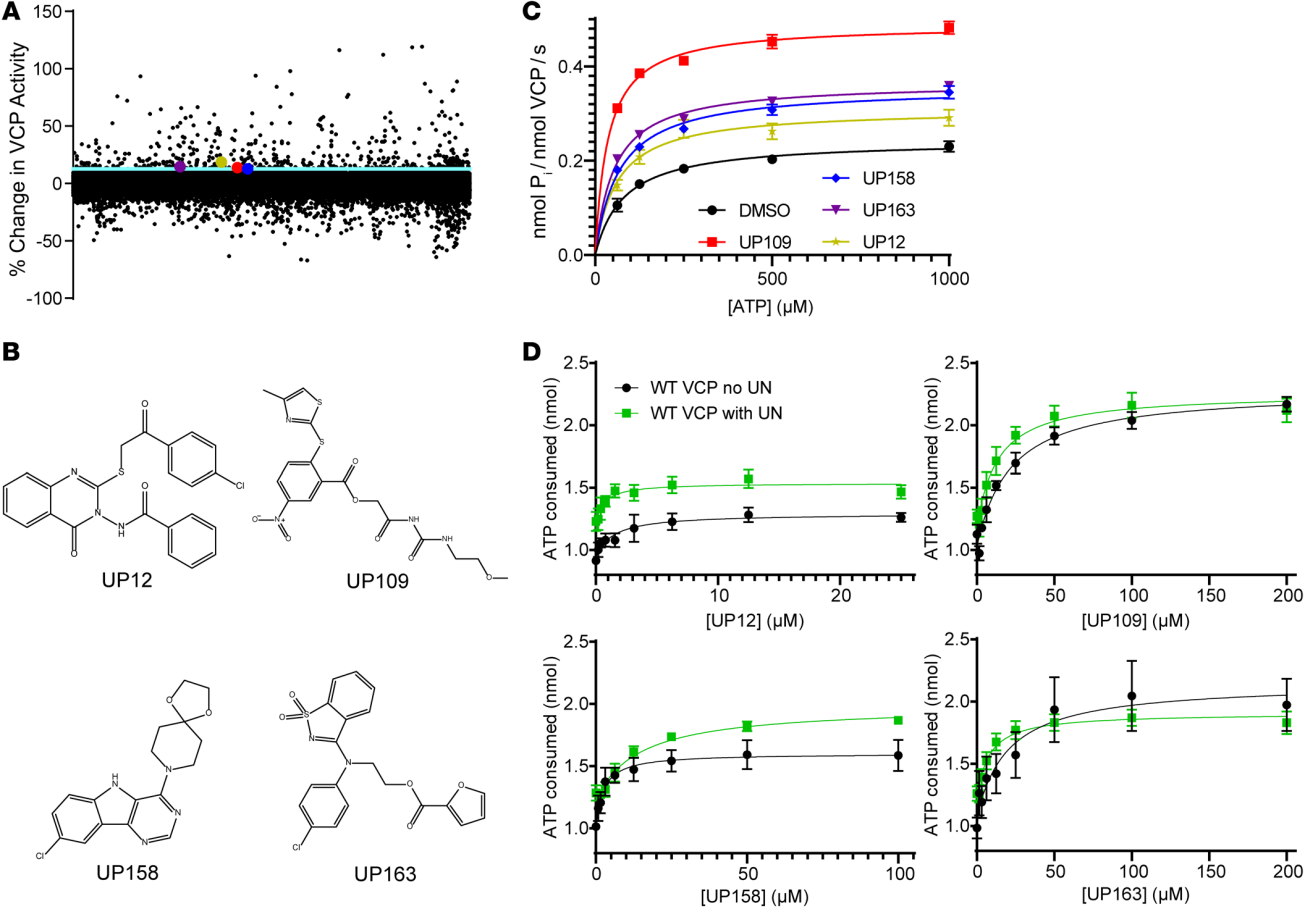
Four structurally heterogeneous compounds increase VCP ATPase activity in vitro. (**A**) Reanalysis of a small molecule screen for VCP modulators (AID 1481) showing percent change of VCP ATPase activity. Three SD above the mean (light blue) was chosen as a cutoff for potential VCP activators. The validated activators are colored (red, UP109; blue, UP158; purple, UP163; yellow, UP12). (**B**) Chemical structures of the 4 compounds validated as in vitro activators of VCP. (**C**) ATPase activity of recombinant VCP measured using an MESG-PNP assay upon treatment with compound versus 2% DMSO control over a range of ATP concentrations. Data were plotted as v_0_/E_T_ versus [ATP] to fit a Michaelis-Menten model using least squares regression for each activator. (**D**) ATPase activity based on a Kinase-Glo assay of recombinant VCP and without (black) or with recombinant UFD1/NPLOC4 (green) versus increasing concentrations of activator. Data were fit to 3-parameter dose curve equation using least squares regression (for **C** and **D**: *n* = 3 with 3 technical replicates per experiment; points are expressed as mean ± SEM).

The above evidence suggests that pathogenic VCP variants are associated with a common pathology (intranuclear inclusions) and a reduction in nuclear VCP localization. Moreover, VCP activity appears to enhance clearance of intranuclear aggregates. These results suggested that enhancing VCP function may counter the deleterious effects of pathogenic VCP variants. To identify small molecule compounds that increase VCP activity, we used a publicly available small molecule high-throughput screen data set that was intended to find VCP inhibitors using a luciferase-based ATPase assay (Kinase-Glo) to measure recombinant VCP activity (PubChem AID 1481). From this data set, potential VCP activator compounds were identified based on a cutoff of greater than 3 SDs above the mean of VCP activity. Compounds were excluded if they were found to be luciferase inhibitors by 2 other independent high-throughput small molecule screens for luciferase activity (PubChem AID 588342 and AID 1891). This exclusion step was necessary because the VCP screen was based on a luciferase readout of ATP remaining in the system where luciferase inhibition and VCP activation would result in similar signal changes (Figure 5A). From these potential activators, we identified a group of 106 structurally heterogeneous compounds that were commercially available for in vitro validation. Using the same Kinase-Glo assay to measure recombinant VCP ATPase activity, 4 compounds, designated UP12, UP109, UP158, and UP163 (colored dots, Figure 5A), significantly increased recombinant WT VCP ATPase activity and did not inhibit luciferase activity (Figure 5, B and D, and Supplemental Figure 6). These results were verified using an orthogonal ATPase assay using MESG-PNP to measure phosphate release (Figure 5C).

UP12 and UP158 exhibited a modest increase of VCP activity (39% and 50% increase, respectively), while UP109 and UP163 increase VCP activity 97% and 104% (Figure 5D and Table 1). UP12 and UP158 had similar EC_50_ values of 1.24 and 2.57 μM, respectively, while UP109 and UP163 had relatively high EC_50_ values of 24.7 and 9.00 μM, respectively. Notably, the relatively high EC_50_ for UP109 and UP163 is partially a reflection of higher maximum activity and not necessarily an indication of lower potency.

VCP ATPase activity can potentially be enhanced by increasing the turnover rate of ATP, increasing the affinity of ATP for VCP, or a combination of the 2. To determine how the activators affected k_cat_ and K_M_ of WT VCP, recombinant VCP with 25 μM activator was assessed for ATPase activity with increasing concentrations of ATP. The k_cat_, indicating the turnover rate of ATP by VCP, was significantly increased in all activator experiments. UP12 had the smallest increase of about 25%, while UP158 and UP163 had a more than 50% increase, and UP109 had a 100% increase in k_cat_ (Figure 5C and Table 1). The K_M_, the concentration of ATP that yields half the maximum rate of ATP turnover by VCP, was only significantly reduced with UP109, which resulted in a more than 2-fold decrease in K_M_ (36.17 μM) compared with DMSO (80.95 μM) (Figure 5C and Table 1). Additionally, the k_cat_-to-K_M_ ratio can be used to determine catalytic efficiency of VCP for ATP. The catalytic efficiency of VCP with DMSO was 3.01 mM^–1^ s^–1^. All compounds increased the k_cat_/K_M_, indicating an improvement in catalytic efficiency. UP12, UP158, and UP163 were similar at 5.03, 5.26, and 6.76 respectively (Table 1). For UP109, a synergistic effect of VCP turnover rate and affinity for ATP is indicated by the more-than 4-fold increase in catalytic efficiency to 13.52, suggesting it may be the most effective of the 4 compounds described.

In vivo, protein cofactors are often required for VCP activity. UFD1 and NPLOC4 (“UN” collectively), allow VCP to recognize ubiquitinated substrates for unfolding upstream of the 26S proteasome. Moreover, VCP ATPase activity in vitro increases in the presence of the UN cofactors (10). To determine how this series of activators affected recombinant VCP in the presence of UN, recombinant UN was used in a 1:1 molar ratio with VCP. Compared to without UN, UP163 had no change in EC_50_, while the EC_50_ of UP109 and UP12 exhibited non-significant decreases from 24.72 μM to 13.36 μM and 1.57 μM to 0.648 μM, respectively. The EC_50_ of UP158 significantly increased from 2.41 μM to 14.30 μM (Figure 5D and Table 1). Maximum percent activity stayed the same for UP158 (150.3% to 152.1%) with UN compared to without UN, but significantly decreased for UP12 (139.0% to 124.6%), UP109 (197.4% to 175.3%), and UP163 (204.0% to 151.8%) (Figure 5D and Table 1). With UN, UP12 had the lowest maximum percent activity, UP158 and UP163 had similar intermediate activities, and UP109 had the highest maximum percent activity. The varied effect of recombinant UN on EC_50_ and maximum percent activity with recombinant VCP may indicate distinct mechanisms of action by the 4 compounds.

**Figure 6:**
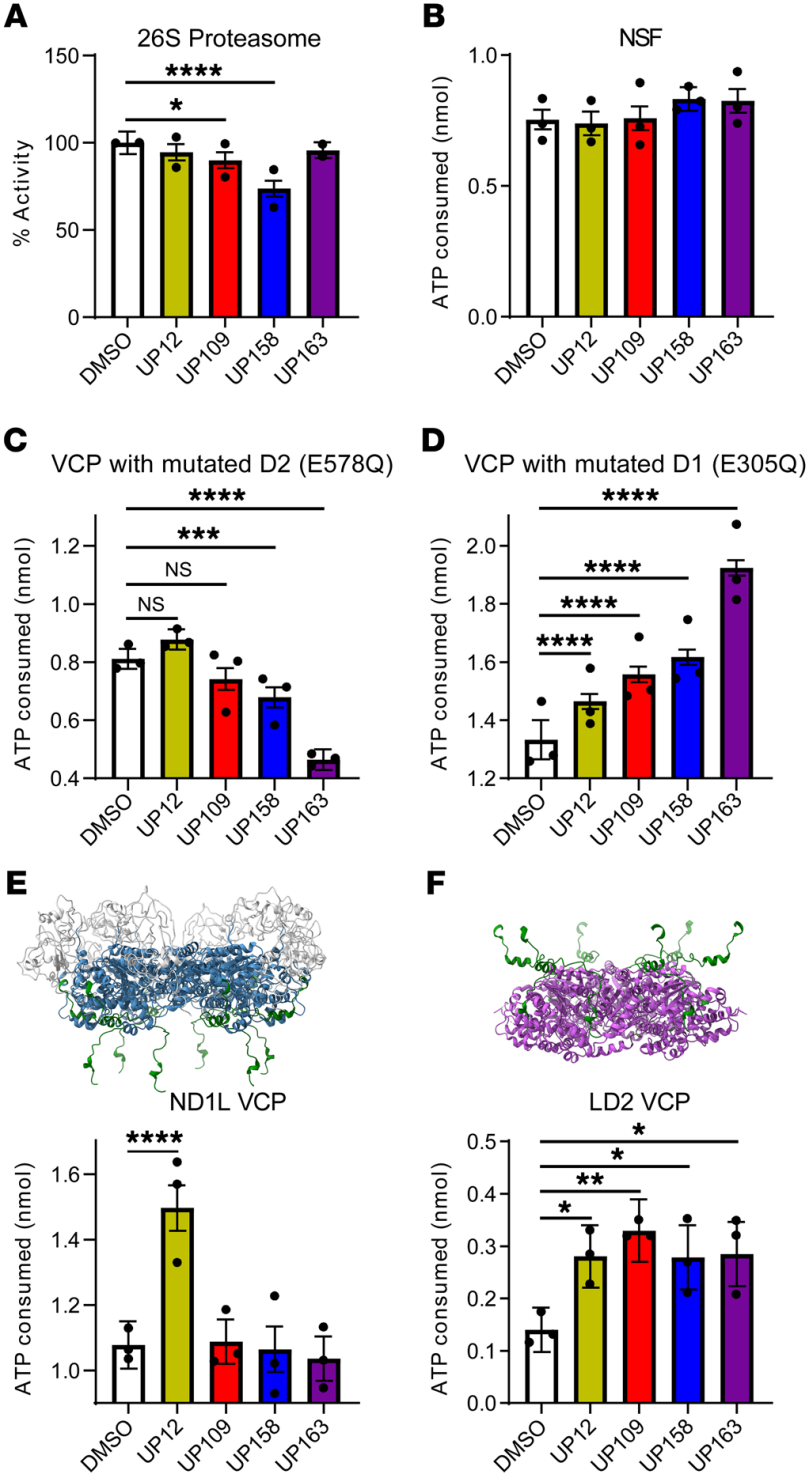
VCP activators exhibit ATPase domain–specific effects. (**A**) 26S proteasome activity with compound or 1% DMSO (*n* = 2 with 3 technical replicated per experiment). (**B**–**F**) ATP consumed as determined by a Kinase-Glo assay with compound or 2% DMSO and recombinant (**B**) N-ethylmaleimidesensitive-factor (NSF), (**C**) VCP (E578Q), (**D**) VCP (E305Q), (**E**) ND1L (aa1-481) VCP, or (**F**) LD2 (aa442-806) VCP (*n* = 3 with 3 technical replicates per experiment; for all panels, points are expressed as β ± SE, LME, *P < 0.05,**P < 0.01; ***P < 0.001; ****P < 0.0001). **D** and **E** show schematics of the VCP truncations used with NTD (white), D1 ATPase domain (blue), linker region shared between ND1L and LD2 (aa442-481) (green), and D2 ATPase domain (purple) (PDB:5FTN).

VCP is a member of the AAA+ ATPase family of proteins that includes many proteins with similar activities in mammals and other organisms. To determine the specificity of our compounds for VCP compared with a similar human AAA+ ATPase, the effect of these compounds on proteasome activity was determined using purified 26S proteasomes and fluorescence of the Suc-LLVY-AMC substrate with 25 μM compound. No compound significantly increased activity of 26S proteasome compared with DMSO, but UP109 and UP158 decreased activity of 26S proteasome (*P* < 0.05 and *P* < 0.0001, respectively; Figure 6A). Recombinant NSF ATPase activity was determined using 25 μM compound. No compound significantly increased ATPase activity of recombinant NSF compared with DMSO, indicating that UP12, UP109, UP158, and UP163 have at least some specificity for VCP over other AAA+ ATPases (Figure 6B). UP109 and UP158, however, may also have counteracting inhibitory effects on the 26S proteasome.

VCP has 2 distinct ATPase domains, D1 and D2, that have distinct functions. The D1 ATPase domain sits above the D2 ATPase domain in the homohexameric VCP double-ring structure. It has been suggested that ATP binding to D1 assists with initiation of substrate unfolding. D2, however, works to continuously unfold a protein substrate once it has been loaded (8, 10). Walker B mutations, which allow ATP to bind, but not be hydrolyzed, were added to the D1 (E305Q) or D2 (E578Q) ATPase active sites in recombinant VCP to isolate D2 and D1 ATPase activity, respectively, in the full-length recombinant VCP. ATPase activity was determined with 25 μM activator or DMSO to understand ATPase domain specificity of the compounds. With an inactive D2 and active D1 (VCP E578Q), only UP12 seemed to increase ATPase activity, though not significantly (*P* = 0.069). UP158 and UP163, interestingly, significantly decreased ATPase activity of the D1 ATPase domain (*P* < 0.001 and *P* < 0.0001, respectively). UP109 also decreased ATPase activity, though less than UP158 and UP163 and not significantly (*P* = 0.074; Figure 6C). However, with an inactive D1 and active D2 (VCP E305Q) all compounds significantly increased D2 ATPase activity (*P* < 0.0001 for all; Figure 6D). These are the first compounds, to our knowledge, that can increase D2 ATPase activity of VCP. UP12 is the only compound, to our knowledge, to increase ATPase activity of both domains.

As a hexamer with a total of 12 ATPase domains, VCP has many allosteric interactions that coordinate activity of D1 and D2 ATPase domains. To understand how the compounds specifically affect the D1 and D2 ATPase domains in the absence of coordination between the 2 ATPase domains, truncations that isolate the D1 and D2 ATPase domain were purified. One construct (aa1-481), termed ND1L, contained the NTD and D1 regions and a linker region (Figure 6E). The other construct (aa442-806), termed LD2, contained a linker region, D2, and the C-terminus (Figure 6F). ATPase activity was again measured with 25 μM compound or DMSO. With the ND1L construct, UP12 was the only compound to increase ATPase activity (*P* < 0.0001; Figure 6E), similar to the full-length VCP E578Q. Moreover, UP109, UP158, and UP163 did not inhibit the ATPase activity of the ND1L construct. The lack of inhibition with the truncated VCP compared with the full-length VCP E578Q indicates that UP109, UP158, and UP163 may bind outside of aa1-481, and the observed inhibition with the full-length VCP E578Q was an allosteric effect sourcing from within the D2 ATPase domain. With the LD2 construct, all compounds significantly increased ATPase activity, similar to full-length VCP E305Q (*P* < 0.05 for UP12, UP158, UP163; *P* < 0.01 for UP109) (Figure 6F). UP12, as the only compound to increase ATPase activity of both ND1L and LD2 likely binds to the linker region present in both truncated proteins (aa442–481), which is known to be required for ATPase activity of truncated VCP proteins (34–36). UP109, UP158, and UP163 likely bind in the D2 ATPase domain.

### Clearance of insoluble intranuclear TDP-4FL aggregates is enhanced by UP109.

**Figure 7:**
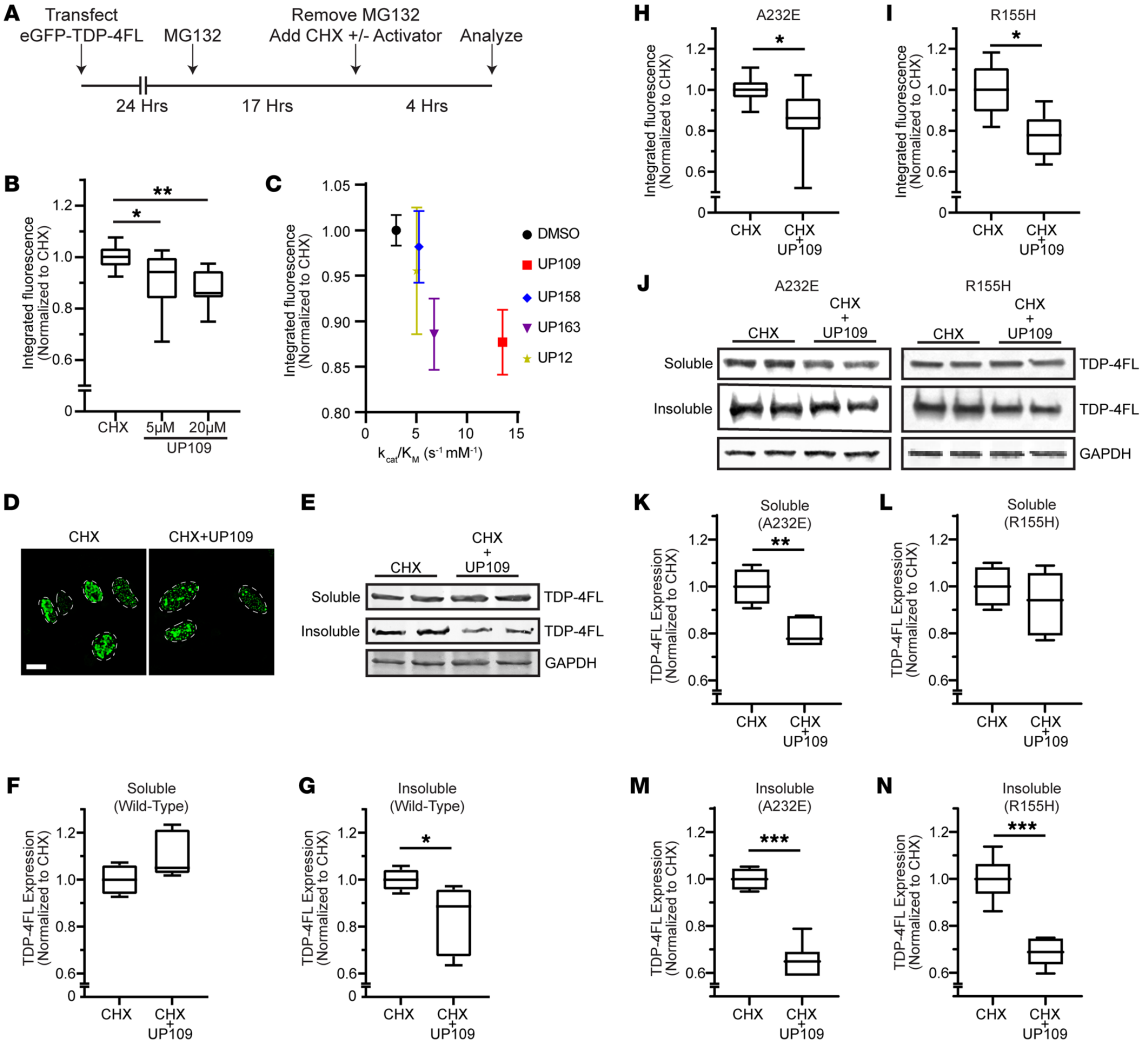
VCP activator enhances clearance of intranuclear TDP-4FL inclusions. (**A**) Schematic of transfection and drug treatment timing to examine the turnover of intranuclear TDP-4FL inclusions. (**B**) Cells expressing GFP-TDP-4FL were treated with MG132 followed by CHX with or without 5 or 20 μM of UP109 and analyzed by flow cytometry (*n* = 4 with 2 technical replicates per experiment; data shown as box-and-whisker plot; LME, *P < 0.05, **P < 0.01). (**C**) Comparison of cellular integrated fluorescence to in vitro k_cat_/K_M_ values for activators plotted as mean ± SEM of integrated fluorescence for activators at 20 μM. (**D**) Representative confocal microscopy images of HeLa cells expressing TDP-4FL after treatment with MG132 followed by CHX with or without 5 μM UP109. Scale bar: 10 μm. (**E**) Immunoblot for myc (TDP-4FL) of soluble and insoluble protein fractions after treatment with MG132 followed by CHX with or without 5 μM UP109. GAPDH shown as a loading control. (**F** and **G)** Quantification of TDP-4FL immunoblots from (**F**) soluble protein fractions (*n* = 3 with 2 technical replicates per experiment; data shown as box-and-whisker plot; LME, *P* = 0.0541) and (**G**) insoluble protein fractions (*n* = 3 with 2 technical replicates per experiment; LME, *P < 0.05). (**H**) A232E and (**I**) R155H cell lines were transfected with GFP-TDP-4FL and then treated with MG132 followed by CHX with or without 5 μM of UP109. Integrated fluorescence intensity was measured by flow cytometry (*n* = 4 with 2 technical replicates per experiment; data shown as box-and-whisker plot; LME, *P < 0.05). (**J**) Immunoblot for myc (TDP-4FL) of soluble and insoluble protein fractions of A232E and R155H cell lines after treatment with MG132 followed by CHX with or without 5 μM UP109. GAPDH shown as a loading control. (**K** and **L**) Quantification of A232E (**K**) and R155H (**L**) TDP-4FL immunoblots from soluble protein fractions. (**M** and **N)** Quantification of A232E (**M**) and R155H (**N**) TDP-4FL immunoblots from insoluble protein fractions (for **K**–**N**: *n* = 3 with 2 technical replicates per experiment; data shown as box-and-whisker plot; LME, **P < 0.01 ***P < 0.001).

Because UP109 modulated the greatest increase in VCP ATPase activity in our in vitro studies, we hypothesized that UP109 would enhance clearance of TDP-4FL aggregates in our cell models. To test whether UP109 would enhance clearance of TDP-4FL aggregates in our cell models, cells expressing GFP-TDP-4FL were first treated with MG132 for 17 hours to build up insoluble intranuclear aggregates followed by removal of MG132 and addition of CHX with or without 5 μM or 20 μM of UP109 for 4 hours (Figure 7A). Flow cytometry revealed that integrated fluorescence intensity was significantly decreased with 5 μM of UP109, and was further reduced with 20 μM of UP109 (*P* < 0.05, *P* < 0.01; Figure 7B). We also tested the effects of UP12, UP158, and UP163 and found that UP163 slightly enhanced clearance of GFP-TDP-4FL aggregates, but UP158 and UP12 did not have a significant effect (*P* < 0.05; Supplemental Figure 7). When the integrated fluorescence data is plotted against the in vitro k_cat_/K_M_ of VCP with ATP, the k_cat_/K_M_ change is a good predictor of cellular effect of the compounds with same trends of efficacy between the 2 experiments (Figure 7C).

To exclude any potential effect of the GFP tag used in these experiments, additional experiments were conducted using a non-GFP–tagged TDP-4FL following the same experimental timing as above (Figure 7A). Cells treated with only CHX exhibited large and dense TDP-4FL aggregates. However, in the presence of 5 μM UP109, TDP-4FL aggregate appeared to be smaller and in some cases revert to small anisosomes (Figure 7D). This was also confirmed with biochemical analysis of soluble and insoluble protein fractions. Immunoblotting for TDP-4FL demonstrated that the addition of 5 μM UP109 resulted in a significant decrease in insoluble TDP-4FL levels compared with cells treated only with CHX (*P* < 0.05; Figure 7, E–G). Collectively, these findings suggest that activation of VCP activity with UP109 enhances clearance of intranuclear TDP-4FL inclusions.

Because accumulation of insoluble intranuclear TDP-4FL was exacerbated in cells harboring A232E and R155H VCP variants (Figure 3), we hypothesized that UP109 would enhance the clearance of insoluble TDP-4FL aggregates in the background of the pathogenic VCP variants. We first tested the 4 activators in vitro against MSP variants of VCP in vitro. We confirmed that UP109 increases ATPase activity of MSP variants of VCP (*P* < 0.0001 for A232E, R155H, and R159H VCP) and saw that it increases their ATPase activity more than the ATPase activity of WT VCP (Supplemental Figure 8). We then tested UP109 in cellular models with MSP variants of VCP. GFP-TDP-4FL was expressed in VCP-A232E and R155H cell lines and integrated fluorescence intensity was measured using flow cytometry following the same experimental paradigm shown in Figure 7A. In both cell lines, 5 μM of UP109 significantly decreased integrated fluorescence intensity compared with the CHX-only treatment condition (*P* < 0.05; Figure 7, H and I), suggesting that UP109 can enhance clearance of GFP-TDP-4FL aggregates in the setting of pathogenic A232E and R155H VCP variants. Similar to the biochemical analysis in WT cells, sequential extraction of soluble and insoluble fractions was also performed. Immunoblots of TDP-4FL in VCP-A232E and R155H cells revealed that cells treated with 5 μM of UP109 resulted in a significant decrease in insoluble TDP-4FL levels in both VCP-A232E cells (Figure 7, J, K, and M) and VCP-R155H cells (Figure 7, J, L, and N) compared with cells treated only with CHX (*P* < 0.001, Figure 7, J, M, and N).

**Figure 8:**
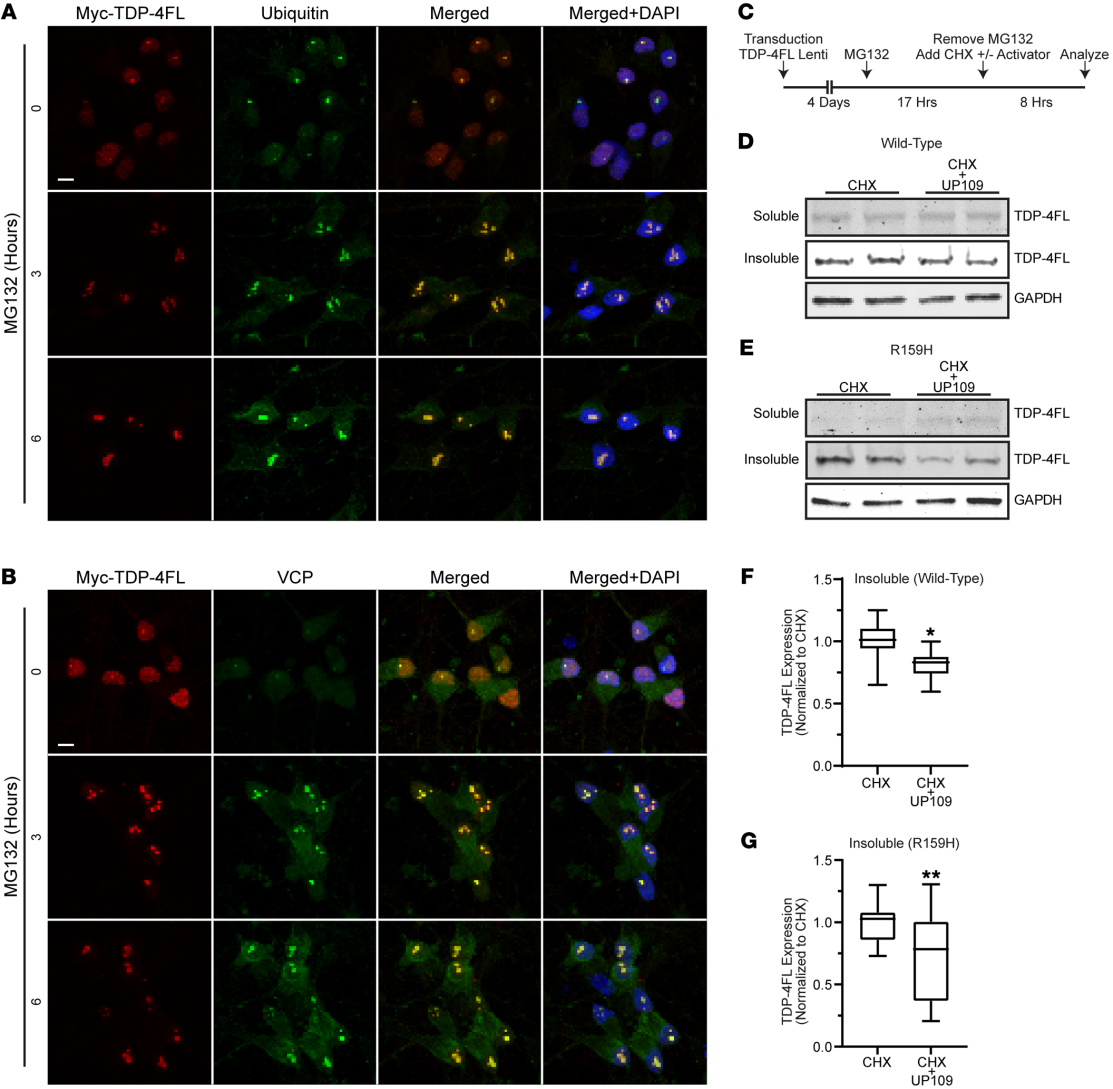
VCP activator enhances clearance of intranuclear TDP-4FL inclusions in iPSC-derived neurons. (**A** and **B**) Immunofluorescence confocal images of iPSC-derived cortical-like neurons expressing TDP-4FL treated with MG132 stained for myc (TDP-4FL, red) and ubiquitin (**A**, green) or VCP (**B**, green). Scale bar: 5 μm. (**C**) Schematic of transfection and drug treatments timing to examine the turnover of intranuclear TDP-4FL inclusions. (**D** and **E**) Immunoblot for myc (TDP-4FL) of soluble and insoluble protein fractions in WT (**D**) and R159H (**E**) neuronal cell lines after treatment with MG132 followed by CHX with or without 20 μM UP109. (**F** and **G**) Quantification of WT (**F**) and R159H (**G**) TDP-4FL immunoblots from insoluble protein fractions (*n* = 3 with 4 technical replicates per experiment; data shown as box-and-whisker plot; LME, **P < 0.01, *P < 0.05).

To test our findings in a more physiologically relevant model, we utilized parental KOLF 2.1J iPSCs and KOLF 2.1J iPSCs with homozygous CRISPR/Cas9 knock-in of VCP variant p.R159H (from the iPSC Neurodegenerative Disease Initiative). A dox-inducible NGN2 piggyBac cassette was expressed in iPSCs to generate cortical-like neurons (37), followed by lentivirus transduction to express TDP-4FL into the neurons. Double immunofluorescence of parental cells expressing TDP-4FL treated with 0, 3, or 6 hours of 4 μM MG132 resulted in larger, irregular TDP-4FL inclusions that colocalized with ubiquitin (Figure 8A) and VCP (Figure 8B) in a similar pattern as what was observed in HeLa cells. To test whether UP109 would enhance clearance of TDP-4FL aggregates in the neurons, cells expressing TDP-4FL were first treated with MG132 for 17 hours to build up insoluble intranuclear aggregates followed by removal of MG132 and addition of CHX with 20 μM of UP109 for 8 hours (Figure 8C). Sequential extraction of RIPA-soluble and -insoluble protein collected after CHX and CHX+UP109 treatment was performed to evaluate the solubility of TDP-4FL protein aggregates. Immunoblot of WT (Figure 8D) and VCP-R159H (Figure 8E) neurons demonstrate that after 8 hours of removal of MG132, soluble TDP-4FL levels were very low. In contrast, the urea fraction contained insoluble TDP-4FL in both WT and VCP-R159H neurons. Moreover, treatment with UP109 resulted in a significant decrease in insoluble TDP-4FL in both WT (Figure 8F, *P* < 0.5) and VCP-R159H (Figure 8G, *P* < 0.01) neurons, suggesting that UP109 is also effective in assisting clearance of TDP-4FL aggregates in a neuronal model.

## Discussion

In this study, we explore VCP’s role in nuclear protein homeostasis and propose that the loss of nuclear VCP function resulting in impaired nuclear proteostasis contributes to MSP pathophysiology. Pathogenic VCP variants affect a variety of extra-nuclear functions including ERAD, autophagy, mitophagy, and endosome maturation (38–44). Thus, while the pathophysiology of MSP is likely to be complex, we concentrate here on the nuclear proteostasis defects associated with pathogenic MSP variants. While there is growing research in understanding how VCP variants alter cellular function, there has been limited knowledge of why VCP variants can affect seemingly distinct organ systems in MSP. Here, we highlight evidence of a common pathologic link between these tissues, showing that in addition to neurons in FTLD-TDP type D and myocytes in IBM, osteoclasts in Paget’s disease of bone contain ubiquitinated intranuclear inclusions. The accumulation of ubiquitinated nuclear inclusions suggested that these diverse diseases may each be associated with a defect in nuclear protein homeostasis. Indeed, MSP affects cell types with a high nuclear proteostasis burden due to being multinucleated (myocytes, osteoclasts) or postmitotic, where proteotoxic load cannot be diluted to daughter cells upon cell division (neurons).

Previous studies have shown that overexpression of pathogenic MSP VCP variants was associated with reduced nuclear VCP expression (28, 29). Comparably, we show that knock-in of A232E or R155H into HeLa cells also resulted in a reduction in nuclear VCP expression levels. The mechanism of VCP nuclear transport has not been clearly defined. Most MSP variants are located between the NTD and D1 cleft of VCP (19). Insight to how this region may affect shuttling has been shown in previous studies where cleavage of the NTD led to a reduction in nuclear VCP expression (28, 45). Additionally, VCP may contain a nuclear localization sequence within the NTD (45). How MSP variants affect nuclear localization remains to be explored. There have been some structural and biochemical studies that suggest VCP with MSP variants prefer the “up” conformation of VCP’s NTD (19), which would obscure the predicted nuclear localization sequence. Cofactor binding, which often occurs toward the N-terminal region of VCP, may also affect exposure of this nuclear localization signal. However, it is difficult to determine if this potential nuclear localization sequence regulates nuclear localization, as it is located at the critical junction between the N-terminus and D1 domains of VCP where mutation of this sequence is likely to have strong effects on VCP’s overall structure and function (19). In addition, posttranslational modification, including phosphorylation of VCP, has been reported to regulate VCP nuclear localization, indicating that the mechanisms that regulate VCP subcellular localization are likely complex (46–49).

VCP’s role in the nucleus has been mostly focused on the role of the yeast VCP homologue, Cdc48, in modulating homeostasis of proteins involved in DNA replication or DNA damage (50–52). However, one study in yeast showed that Cdc48 is important for nuclear protein quality control by facilitating degradation of highly insoluble substrates downstream of the nuclear ubiquitin-protein ligase, San1 (24). Many MSP in vivo models have looked at VCP-dependent defects of mitochondria function, autophagy, and endolysosomal processing; however, few have explored VCP’s role in nuclear protein homeostasis. Earlier MSP mouse models of transgenic expression of MSP variants recapitulated multiple MSP disease phenotypes and also showed some evidence of cytoplasmic TDP-43 pathology in the brain (53). However, while MSP variants of VCP cause cytoplasmic VCP defects in many model systems, MSP variants in humans lead specifically to FTLD-TDP Type D where TDP-43 inclusions are primarily localized within nuclei, which is a phenotype that has not been seen in mouse models.

To specifically understand VCP’s role in intranuclear inclusion formation, we developed a cell model of intranuclear TDP-43 inclusions using expression of an RNA binding–deficient construct of TDP-43, TDP-4FL. In cells expressing TDP-4FL, proteasome inhibition led to larger TDP-4FL aggregates and decreased TDP-4FL solubility. This phenotype was exacerbated in A232E and R155H cells, suggesting that these MSP variants were associated with a loss of nuclear VCP function that resulted in an accumulation of insoluble TDP-4FL aggregates. Even without TDP-4FL, we saw an increase in insoluble nuclear puncta with proteasome inhibition, further indicating that A232E and R155H cells have a baseline defect in nuclear VCP function. While the mechanisms described here focus on the accumulation and clearance of intranuclear TDP-43 protein aggregates, neuropathology studies have suggested that there may also be widespread nuclear clearance of normal nuclear TDP-43 protein independent of intranuclear TDP-43 aggregation, which suggests that additional mechanisms may be important in the pathogenesis of this disease (16).

Understanding whether pathogenic VCP variants cause disease due to a gain or loss of function is crucial to developing therapies to target disease. In vitro studies have shown that most MSP variants lead to an increase in VCP’s ATPase activity in vitro, suggesting a toxic gain of function. In a drosophila model of MSP, expression of VCP variants negatively altered mitochondrial morphology, which was reversed upon treatment with the VCP inhibitor CB5083 (54). Additionally, use of CB5083 in a R155H/R155H mouse model of MSP showed improved markers of muscle pathology (55). This raises the possibility that pathogenic VCP variants may affect certain cellular phenotypes through mechanisms associated with a gain of VCP function. Conversely, conditional knock-out of VCP in young mice or conditional knock in of R155C variants into neurons lead to cortical brain atrophy and the accumulation of insoluble TDP-43 (22). We posit that disease models should mirror the underlying pathology observed in human tissues, namely the presence of ubiquitinated intranuclear inclusions. Using such a model and consistent with a loss of nuclear VCP function, our studies show that pathogenic VCP variants A232E or R155H or pharmacological inhibition of VCP with CB5083 prevented proper clearance of intranuclear TDP-4FL aggregate inclusions. This result raises the possibility that VCP inhibition may be deleterious in the setting of MSP and that increasing VCP activity could be therapeutically beneficial.

While many VCP inhibitors have been identified for various therapeutic purposes, identification of VCP activators has been limited. We used a publicly available high-throughput screen of VCP to identify 4 novel activators that have distinct mechanisms of action from 2 previously published compounds that moderately increase D1 ATPase activity only (56, 57). The compounds identified here all increase D2 ATPase activity, with 1 compound, UP12, increasing both D1 and D2 ATPase activity. The exact roles of the D1 and D2 ATPase domain are not fully understood; however, in vitro data would suggest that the D1 ATPase domain needs to bind, but not hydrolyze, ATP for unfoldase activity, while the D2 domain’s ATPase activity is required for unfoldase activity of VCP (8, 10). We show that the most active compound, UP109, can reduce insoluble nuclear aggregates in WT and mutant cells by boosting the clearance of nuclear TDP-43 aggregates. Our data, combined with previous work (56, 57), suggest that there may be multiple mechanisms of action by which small molecule activators can yield beneficial activation of VCP in cellular systems. What remains to be understood is how these compounds affect VCP’s unfoldase activity and how they affect VCP’s structure and ATPase activity while unfolding a polypeptide. Additionally, further work should be done to characterize if the compounds’ beneficial effect is as simple as increasing VCP activity. If this is the case, since UP109 appears to improve MSP variant pathology in our cellular models, this would suggest that pathogenic MSP variants cause disease via a loss of normal nuclear function.

In summary, our study proposes a new mechanism of disease pathogenesis for MSP that potentially links pathological findings seen in brain, muscle, and bone. We highlight the importance of VCP in maintaining nuclear protein homeostasis and its role in the clearance of insoluble, nuclear TDP aggregates. Additionally, we find that disease-linked VCP variants potentiate the accumulation of TDP inclusions,which can be alleviated by a novel VCP activator. Because VCP is involved in multiple cellular processes, we cannot ignore the likelihood that pathogenic VCP variants result in defects across multiple VCP-dependent protein homeostasis pathways over time, including the various extra-nuclear functions that VCP is known to affect. Thus, further work in understanding the degree in which VCP variants affect different disease-relevant pathways can pave the way for continued development of disease modifying therapies.

## Methods

### Sex as a biologic variable.

Both sexes were included in our pathologic analysis of bone. Although the number of cases examined was relatively low due to the rarity of Paget’s disease of bone biopsies, differences according to sex were not observed.

### Cell culture and plasmid transfections.

WT and knock-in HeLa cell lines harboring A232E or R155H VCP variants were maintained at 37°C and grown in DMEM high glucose (Invitrogen) supplemented with 10% FBS (Atlanta Biologicals) and 2 mM L-Glutamine (Invitrogen). For transfection of CRISPR components, 1.5 μg Cas9/gRNA containing plasmid and 1.5 μg repair template were cotransfected into 6-well dishes using Lipofectamine 3000 (Invitrogen) with a 3:1 transfection reagent to DNA ratio for 48 hours. For transient transfections, 1.5 μg of plasmid was transfected into 6-well dishes using Fugene HD (Promega) with 3:1 transfection reagent to DNA for 24 hours. Myc-TDP-43 and myc-TDP-4FL plasmid were gifted from the Center for Neurodegenerative Disease Research (CNDR, University of Pennsylvania, Philadelphia, PA, USA). GFP-TDP-4FL was generated by introducing F147/149/229/231L mutations using site-directed mutagenesis with QuickChange XL kit (Agilent Technologies) into GFP-TDP-43 plasmid (gifted from CNDR).

### Drug treatments and reagents in cell experiments.

For proteasome inhibition, cells expressing myc-TDP-43 or myc-TDP-4FL were treated with 4 μM of MG132 (M8699, Sigma-Aldrich) for 0, 3, or 6 hours. For recovery experiments, cells expressing myc-TDP-4FL or GFP-TDP-4FL were treated with 4 μM of MG132 for 3 hours followed by removal of MG132 and the addition of 30 μg/μL of CHX (C1988, Sigma-Aldrich) with DMSO or 2.5 μM of CB5083 (19311, Cayman Chemical Company) for 6 hours. To test efficacy of UP109, UP158, UP163, or UP12 activator in HeLa cells, cells expressing myc-TDP-4FL or GFP-TDP-4FL were treated with 4 μM of MG132 for 17 hours followed by removal of MG132 and the addition of 30 μg/μL of CHX with DMSO or 5 μM or 20 μM of compound for 4 hours. To test efficacy of UP109 activator in neurons, cells expressing myc-TDP-4FL were treated with 4 μM of MG132 for 17 hours followed by removal of MG132 and the addition of 30 μg/μL of CHX with DMSO or 20 μM of UP109 for 8 hours. Drug treatments were performed on neurons 7 days after lentiviral transduction and 14 days after neuronal induction.

### Sequential protein extraction.

To collect soluble and insoluble protein fractions, cells were subject to sequential extraction using buffers of increasing strengths. Cells were cultured in a 6-well dish and pelleted by centrifugation. Cell pellets were washed with cold DPBS and resuspended in cold RIPA buffer containing 150 mM NaCl, 1% Triton X-100, 0.5% sodium deoxycholate, 0.1% SDS and 50 mM Tris (pH 8.0) (Thermo Fisher Scientific; Sigma-Aldrich; Invitrogen) supplemented with protease inhibitors. Samples were sonicated and centrifuged at 100,000*g* for 30 minutes at 4°C. Supernatant was collected as the RIPA-soluble fraction. The pellet was then reextracted in RIPA buffer to remove residual soluble proteins. The insoluble pellet was extracted in 1/3 of the starting volume of urea buffer containing 7 M Urea, 2 M Thiourea, 4% CHAPS, and 30 mM Tris (pH 8.5) (Thermo Fisher Scientific; Sigma-Aldrich), sonicated, and centrifuged at 100,000*g* for 30 minutes at 22°C. The supernatant was collected as the RIPA insoluble fraction.

### Total protein extraction.

To collect total protein lysates, cells were cultured in a 6-well dish and pelleted by centrifugation. Prior to lysing the cells, the number of cells were counted for equal loading with Western blot analysis. Cell pellets were washed with cold DPBS and resuspended in cold RIPA buffer. Samples were sonicated and centrifuged at 21,000*g* to remove cellular debris. Supernatant was collected as total protein lysate.

### Nuclear protein isolation by sucrose gradient.

To collect nuclear protein lysate, cells were cultured on a 10 cm dish and pelleted by centrifugation. Cell pellets were washed with cold DPBS and resuspended in 0.25M sucrose in TKM buffer (50mM Tris-HCl, pH 7.5, 25mM KCl, 14mM MgCl_2_) supplemented with protease inhibitors (Thermo Fisher Scientific). The resuspended cell pellet was lysed using a dounce homogenizer. The homogenate was mixed with 2.3 M sucrose with TKM buffer to yield a 1.6 M sucrose solution (Thermo Fisher Scientific). The 1.6 M sucrose solution was then added atop 3 mL of 1.8 M (in TKM) sucrose cushion buffer and spun at 100,000*g* for 2 hours at 4°C (Sw-41Ti rotor). After ultracentrifugation, the remaining pellet was resuspended in PBS. Nuclei were counted for equal loading on Western blot analysis.

### Immunoprecipitation.

Myc immunoprecipitation was carried out using Anti-c-Myc Magnetic Beads (Pierce, Thermo Fisher Scientific). Beads were prepared following the manufacturers instruction and incubated with urea-soluble fractions diluted in 25 mM Tris, 0.15 M NaCl, and 0.05% Tween-20 detergent containing 100 mM N-Ethylmaleimide and 5 mM of EDTA (Thermo Fisher Scientific; Bio-Rad; Sigma-Aldrich; Invitrogen) to preserve ubiquitination of protein lysates, overnight at 4°C. To elute samples from beads, Laemmli sample buffer containing 0.5 mM dithiothreitol was added directly to beads and heated for 10 minutes at 99°C.

### Western blot and antibodies.

Protein concentration was determined using BCA reagent (Thermo Fisher). A total of 25–40μg of protein was run on 7.5%–15% sodium dodecyl sulfate-polyacrylamide gel electrophoresis (SDS-PAGE) gel and transferred using the Trans-Blot Turbo system (Biorad) to nitrocellulose membranes. Membranes were blocked using 5% nonfat milk and incubated in primary antibodies in TBS with 0.1% Tween-20 overnight at 4°C. For immunoprecipitation experiments, nitrocellulose membranes were transferred using Mini Trans-Blot Cell (Biorad) overnight. IR dye secondary antibodies (Licor Biosciences) were used to detect protein using a Licor Odyssey. Primary antibodies used include mouse anti-Myc (9E10 (Cell Center Stockroom, University of Pennsylvania), rabbit anti-Myc (ab9106, Abcam), rabbit anti-TDP-43 (C2089, gift from CNDR), mouse anti-VCP (NB120-11433, Novus Biologicals), mouse anti-VCP (MA3-004, Invitrogen), rabbit anti-ubiquitin (43124, Cell Signaling Technology), rat anti Nup98 (ab50610, Abcam), rabbit anti Hsp90 (4874, Cell Signaling Technology), and rabbit anti-GAPDH (2118, Cell Signaling Technology).

### Immunofluorescence.

HeLa Cells were plated onto glass coverslips and transfected with TDP-4FL using Fugene HD for 24 hours. Cells were fixed with 4% paraformaldehyde (Electron Microscopy Sciences), permeabilized with 0.1% Triton-X (Thermo Fisher Scientific), and blocked with 2% FBS DPBS. Primary antibodies were diluted in 2% FBS DPBS. Cells were stained with 300 nM DAPI, and coverslips were mounted onto glass slides using ProLong Glass Antifade Mountant (Thermo Fisher Scientific). Confocal images were obtained using a Leica TCS SPE laser scanning confocal microscope. Primary antibodies used include mouse anti-Myc (9E10), rabbit anti-Myc (ab9106), mouse anti-VCP (NB120-11433), and mouse anti-ubiquitin (ST1200, Millipore) with Alexa Flour 488 or 568 (Invitrogen) as secondary antibodies.

### Image analysis of nuclear to cytoplasmic fluorescence intensity ratio.

Confocal images of immunofluorescence staining for VCP in WT or knock-in A232E and R155H VCP HeLa cells were analyzed using Fiji (ImageJ). Nuclei and cytoplasmic regions were segmented and fluorescence intensity was determined for each subcellular region. Cytoplasmic intensity values were divided by nuclear intensity value for each individual cell to determine the fluorescence intensity ratio of nuclear-to-cytoplasmic VCP expression.

### RNA isolation and qPCR.

Total RNA was isolated from WT, VCP-A232E, and VCP-R155H HeLa cells using Qiagen RNeasy Mini kit (74104, Qiagen) following the manufacturer’s recommendations including DNAse I digestion. Isolated RNA was quantified using a Qubit 3.0 fluorometer (Q33216, Invitrogen). cDNA was prepared using the High Capacity RNA-to-cDNA Kit (4387406, Applied Biosystems). qPCR was carried out using FastStart Universal SYBR Green mastermix (Roche) on a StepOne Plus RealTime PCR Machine (Life Technologies) using standard cycling. RNA levels of TDP-43 were normalized to the geometric mean of ACTB and GPS. Primers that were used are as followed: TDP-43 Forward – GGCGCTGTACAGAGGACATGA; TDP-43 Reverse – ACATCCCCGTACTGAGAGAAGAAC; GPS Forward – AAGATGCTGGACGAGATGAAGGA; GPS Reverse – ACGGTTGCGAATCTGGGTGTA; ACTB Forward – GCCCTGAGGCACTCTTCCA; ACTB Reverse – ATGCCACAGGACTCCATGC.

### Flow cytometry and data analysis.

HeLa cells were collected using trypsin and pelleted at 1000*g* for 3 minutes. Cell pellets were resuspended in colorless DMEM (Invitrogen) supplemented with 2% FBS and filtered through a BD tube with cell strainer (BD Biosciences) for flow cytometry analysis on LSR II (BD Biosciences). Data analysis was performed on FlowJo v10 software. Integrated fluorescence intensity was determined by multiplying percent positive GFP and GFP median fluorescence values.

### ATPase activity assays.

Experiments with varied activator concentration and screening were measured using the Kinase-Glo Luminescent Kinase Assay (Promega). Reactions were incubated at 25°C for 1 hour then reduced to 4°C in a thermocycler consisting of 50 nM of recombinant VCP with or without 50 nM UFD1/NPLOC4 in 50 uL ATPase buffer (25 mM HEPES, pH 7.5, 100 mM NaCl, 10 mM MgCl_2_) at 2% DMSO with 62.5 μM ATP and a range of activator concentrations (Gibco, Sigma-Aldrich). Equal volumes of reaction and Kinase-Glo reagent were incubated for 20 minutes at room temperature in a 96 well white flat bottom plate (Corning). Luminescence was measured in a microplate reader (Spark 20M, Tecan).

All other experiments were performed using the EnzCheck phosphatase assay kit (Molecular Probes). ATPase activity was measured in a 96-well flat bottom plate (Thermo Fisher Scientific). 50 nM VCP was incubated in 100 μL ATPase buffer with 2% DMSO with 25 μM compound and various ATP concentrations. The absorbance at 360 ± 5 nm was measured for more than 15 minutes at 25°C. Phosphate release was quantified based on a phosphate standard curve performed on the same plate. Initial enzyme velocity was determined by fitting the linear portion of the data to a linear regression model.

Experiments with varied compound concentration were fit to a 3-parameter dose curve with asymmetric profile likelihood 95% confidence intervals in GraphPad Prism 9 (GraphPad). Experiments with varied ATP concentration were fit to a Michaelis-Menten model with asymmetric profile likelihood 95% confidence intervals. Significance was determined for experiments with fixed compound or fixed ATP concentration using a linear mixed-effects model.

### 26S proteasome activity.

26S proteasome and substrate suc-LLVY-AMC were purchased (LifeSensors, Inc.). Fluorescence was measured in a microplate reader using a 96-well optical bottom black plate (Thermo Fisher Scientific). 18 μg/μL 26S proteasome was incubated in 100 μL proteasome buffer (20 mM HEPES, pH 7.5, 0.5 mM EDTA, 0.05% Triton-X) with 100 μM suc-LLVY-AMC, 1 mM ATP, and 25 μM compound or 1% DMSO. Fluorescence was measured at excitation 380 ± 10nm and emission 460 ± 10nm for more than 15 minutes at 37°C. Initial velocity was determined by fitting the linear portion of the data to a linear regression model. Significance was determined for using a linear mixed-effects model.

### Statistics.

1- or 2-way ANOVA statistical analysis and Dunnett’s multiple comparison post hoc analysis were performed using GraphPad Prism 9. 3-parameter dose curve with asymmetric profile likelihood 95% confidence intervals and Michaelis-Menten model with asymmetric profile likelihood 95% confidence intervals were fit in GraphPad Prism 9. Linear mixed effects (LME) regression models were performed using nlme package, and Tukey’s multiple comparison post hoc analysis were performed using emmeans package in RStudio. Box and whiskers plot: whiskers represent minimum to maximum; line represent median, bounds represent 25th to 75th percentiles. *P* value < 0.05 was considered significant.

### Study approval.

This study includes analysis of deidentified/anonymized, leftover biopsy specimens without any contact with human participants, and is therefore not considered human subjects research. Animal studies were not performed.

### Data availability.

Data used to generate the figures are provided in the Supporting Data Values file accompanying this manuscript. See Supplemental Methods for additional study details and descriptions.

## Author contributions

JMP, BCC and EBL conceived of and designed the experiments, analyzed results, and wrote the manuscript. JMP, BCC, ATN, DDB, NFD, and CNM performed the experiments. All authors edited the manuscript.

## Supplementary Material

Supplemental data

Unedited blot and gel images

Supporting data values

## Figures and Tables

**Table 1:**
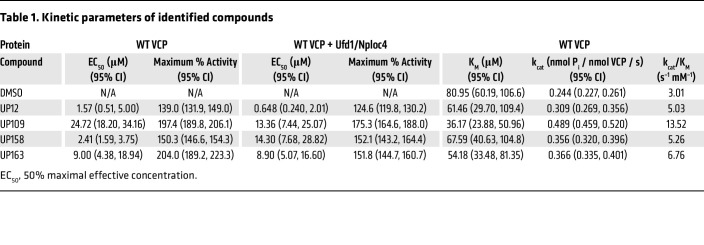
Kinetic parameters of identified compounds

## Version 1 05/24/2024

In-Press Preview

## Version 2 07/15/2024

Electronic publication
