# Supplementary material for: VCP activator reverses nuclear proteostasis defects and enhances TDP-43 aggregate clearance in multisystem proteinopathy models
Source: J Clin Invest. 2024 May 23;134(14):e169039. doi: 10.1172/JCI169039 (PMC11257039; doi:10.1172/JCI169039)
Supplement: Supplemental data [file jci-134-169039-s010.pdf]

VCP activator reverses nuclear proteostasis defects and enhances TDP-43 aggregate clearance multisystem proteinopathy model

Authors: Jessica M. Phan*, Benjamin C. Creekmore*, Aivi T. Nguyen, Darya D. Bershadskaya, Nabil F. Darwich, Carolyn N. Mann, Edward B. Lee

Affiliations:

Translational Neuropathology Research Laboratory, Department of Pathology and Laboratory Medicine, Perelman School of Medicine at the University of Pennsylvania, PA, USA.

*Equal contributions

Correspondence to:

Edward B. Lee

613A Stellar Chance Laboratories

422 Curie Blvd

Philadelphia, PA 19104

(215) 898-0908

edward.lee@pennmedicine.upenn.edu

Conflict-of-interest statement: A patent application related to this work is pending. The authors have declared that no additional conflict of interest exists.

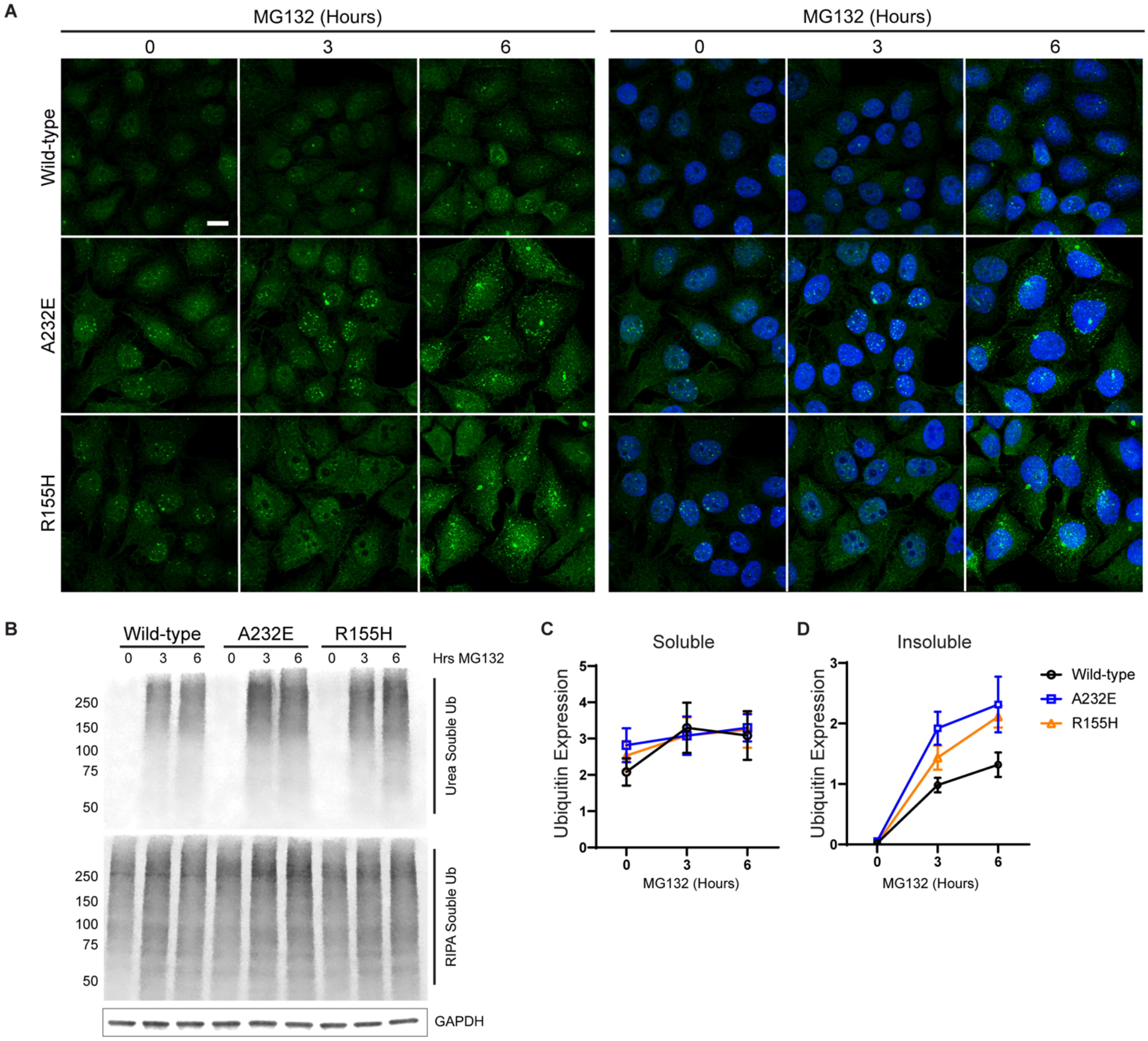

Figure S1: Proteasome inhibition increases ubiquitination in MSP variant.
(A) Immunofluorescence confocal images of HeLa cells treated with 4μM MG132 for 0, 3, or 6 hours stained for ubiquitin (green) or DAPI (blue). Scale bar, 10μm. **(B)** Immunoblots for ubiquitin from soluble and insoluble protein fractions from HeLa cells treated with 4μM MG132 for 0, 3, or 6 hours. GAPDH provided as a loading control. **(C-D)** Quantification of immunoblots for (C) soluble and (D) insoluble ubiquitinated proteins in WT, A232E, and R155H cells treated with 0, 3, 6 hours of 4 μM MG132. (n = 3, results expressed as mean ± SEM over time; two-way ANOVA for soluble protein: time *P < 0.05, genotype P = 0.2264, time x genotype P = 0.1290; for insoluble protein: time **P < 0.01, genotype *P < 0.05, time x genotype P = 0.0629)

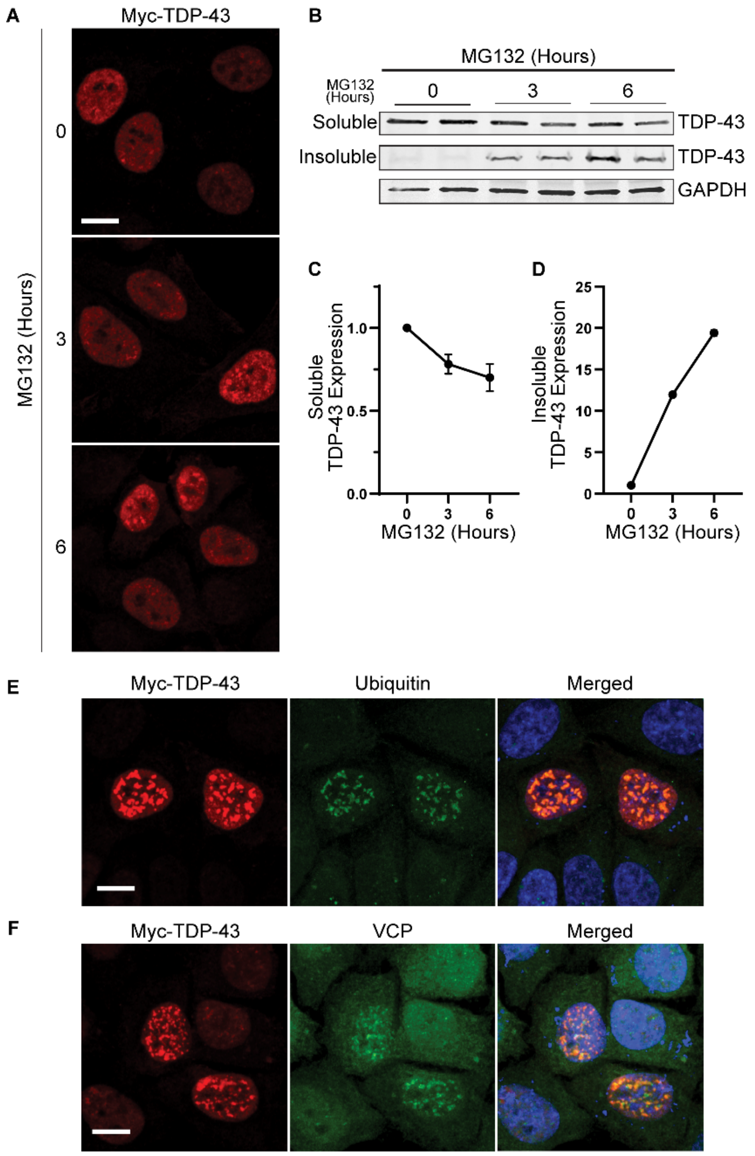

Figure S2: Proteasome inhibition induces insoluble intranuclear TDP-43 inclusions that colocalize with ubiquitin and VCP.

(A) Confocal images of myc (TDP-43) immunofluorescence in HeLa cells expressing myc-TDP-43 treated with 4μM MG132 for 0, 3, or 6 hours. **(B)** Immunoblot for myc (TDP-43) of soluble and insoluble protein fractions from HeLa cells expressing myc-TDP-43 with 4μM MG132 for 0, 3, or 6 hours. GAPDH shown as a loading control. Quantification of **(C)** soluble or **(D)** insoluble TDP-4FL immunoblots. (Soluble, n = 2 experiments each with 2 replicate replicates per condition, results are expressed as mean ± SEM over time. One-way ANOVA, P = 0.170 for soluble protein, *P < 0.05 for insoluble protein). **(E-F)** Immunofluorescence confocal images of HeLa cells expressing TDP-43 treated with 4uM MG132 for 3 hours stained for myc (TDP-43, red) and either ubiquitin (E, green) or VCP (F, green). Scale bar, 10μm.

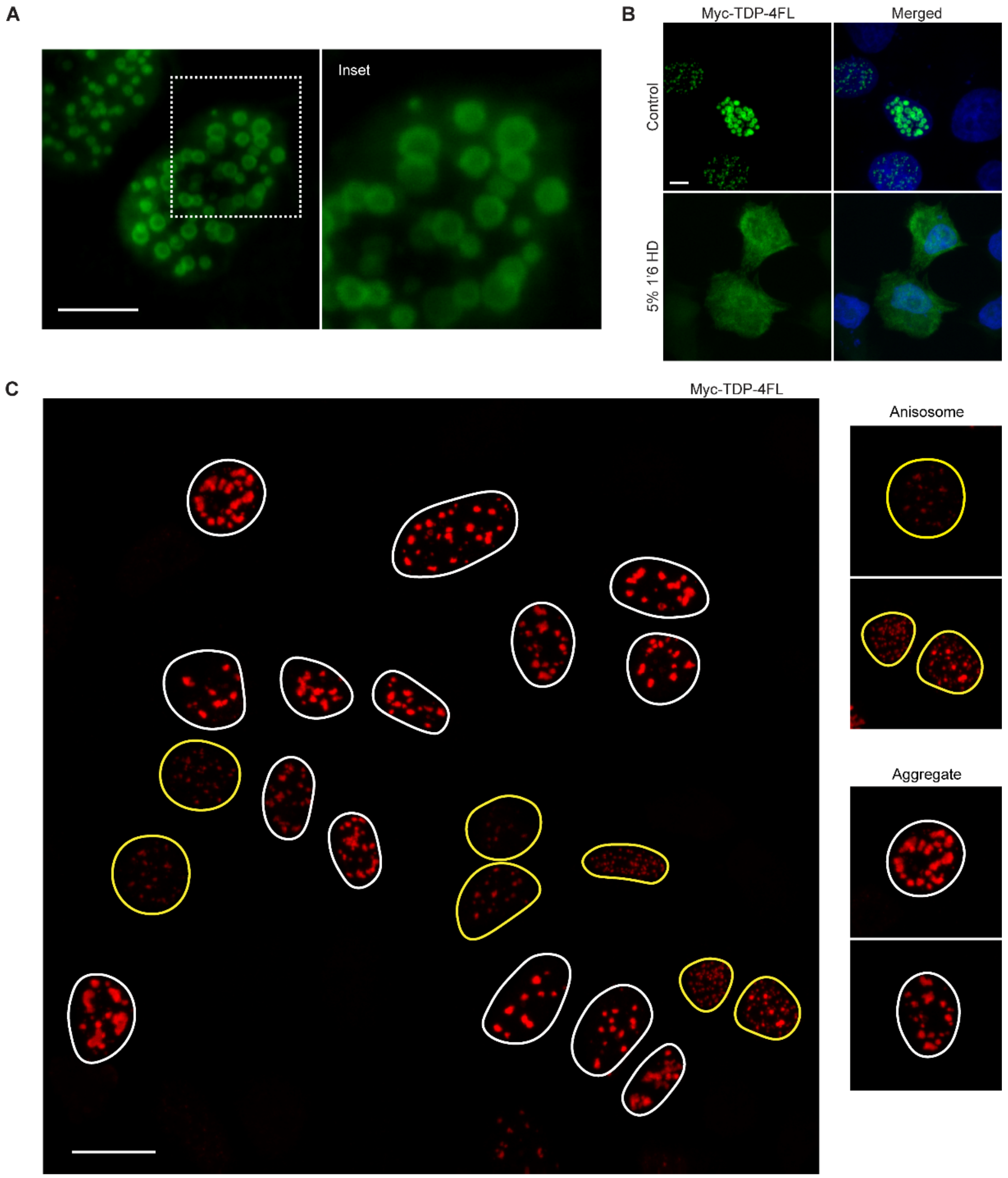

Figure S3: TDP-4FL expression in HeLa cells exhibit anisotropic qualities and displays mix morphologies with MG132 treatment.

(A) High power magnification immunofluorescence confocal images of HeLa cells expressing TDP-4FL stained for myc-TDP (green) Scale bar, 5 µm. **(B)** Immunofluorescence confocal images of HeLa cells treated with or without 5% 1'6-Hexanediol for 10 minutes stained for myc-TDP (green) or DAPI (blue). Scale bar, 5 µm. **(C)** Representative confocal microscopy immunofluorescence confocal images of HeLa cells expressing TDP-4FL treated with 4µM MG132 for 3 hours stained for myc (TDP-4FL, red). Nuclei outlined in yellow represent cells that were categorized as anisosome morphology. Nuclei outline in white represent cells that were categorized as aggregate morphology. Cells with approximately more than 50% anisosome inclusions were categorized as anisosome morphology whereas cells with approximately more than 50% aggregate-like inclusions were categorized as aggregate morphology. Binucleate cells were excluded from the total counts. Scale bar, 10 µm.

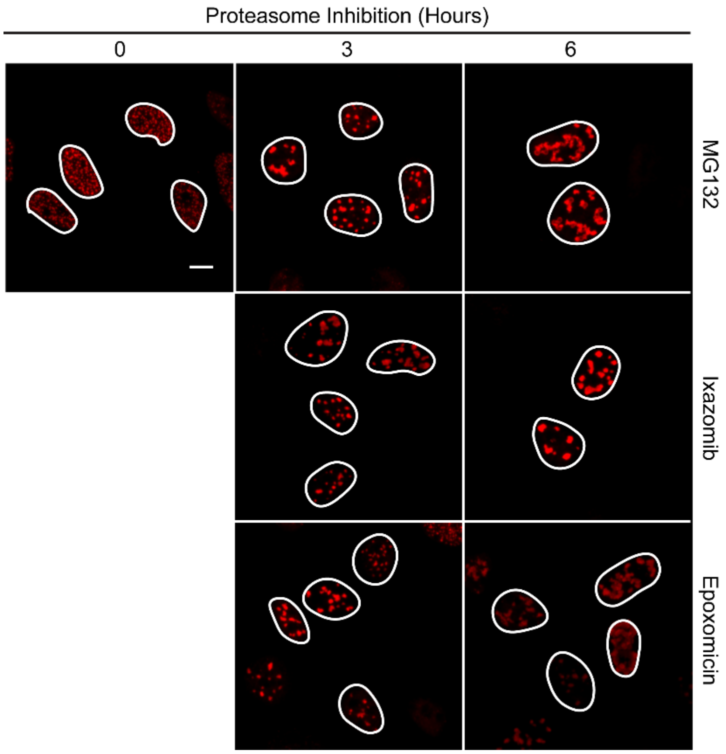

Figure S4: Proteasome inhibition with ixazomib and epoxomicin promotes insoluble intranuclear TDP-4FL inclusions similar to MG132 and is irreversible with epoxomicin.
Immunofluorescence confocal images of HeLa cells expressing TDP-4FL treated with 4μM MG132, 1μM of ixazomib, or 1μM of epoxomicin for 0, 3, or 6 hours stained for myc (TDP-4FL, red). Scale bar, 10μm.

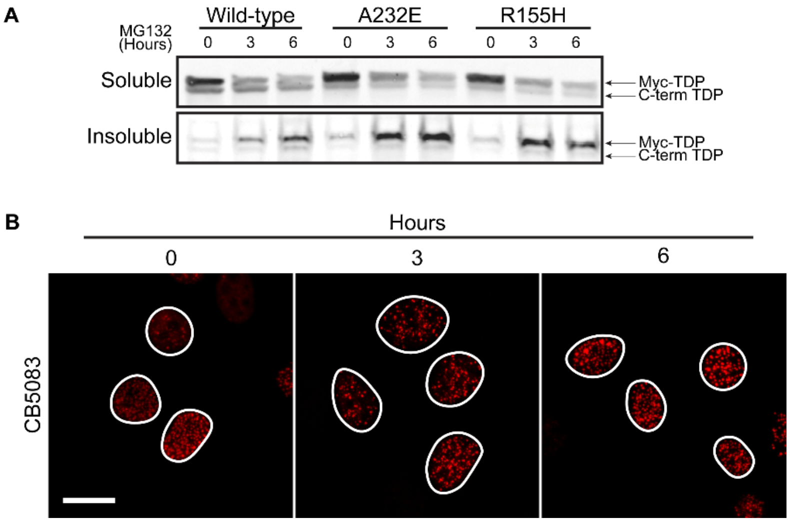

Figure S5: CB5083 treatment only does not promote formation of insoluble intranuclear TDP-4FL aggregates.
(A) The blot from figure 3F was re-probed for endogenous TDP-43 protein (rabbit anti-C-terminal TDP-43 antibody) in WT, A232E, and R155H cells treated with 0, 3, 6 hours of 4µM MG132. **(B)** Immunofluorescence confocal images of HeLa cells expressing TDP-4FL treated with 2.5µM of CB5083 for 0, 3, or 6 hours stained for myc (TDP-4FL, red). Scale bar, 10µm.

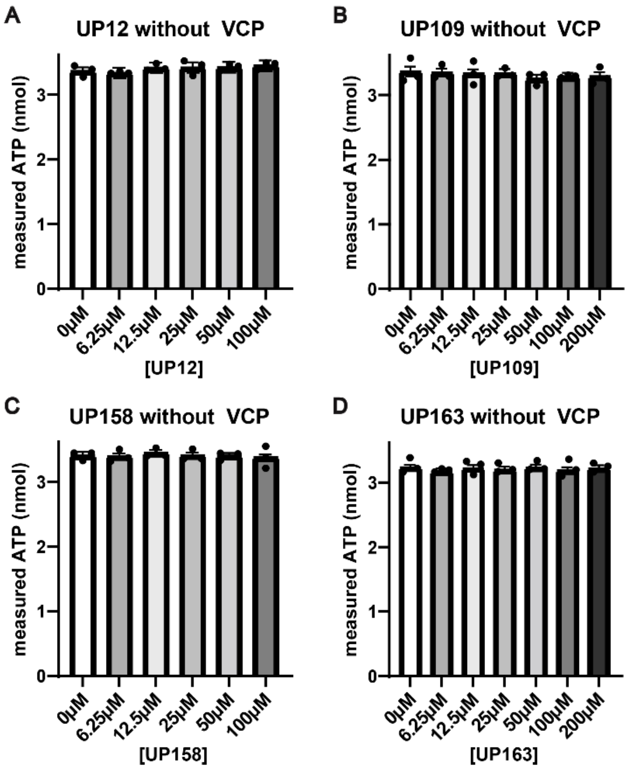

Figure S6. VCP activator compounds do not inhibit luciferase.
(A-D) Measured ATP as determined by a Kinase-Glo assay of 62.5μM ATP in the presence of increasing concentrations of compound without recombinant VCP present. (for all panels: n=3 with 4 technical replicates per experiment, points are expressed as beta ± SE, linear mixed effects model, P>0.05 for each compound).

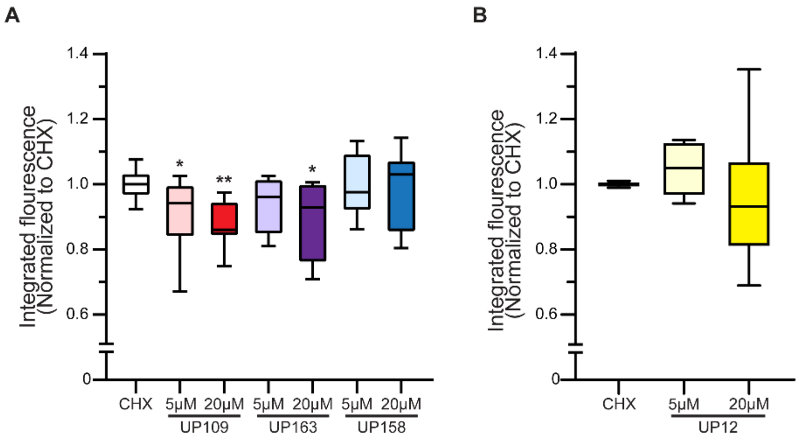

Figure S7: VCP activators UP109 and UP163 enhances clearance of TDP-4FL inclusions.
(A) Cells expressing GFP-TDP-4FL were treated with 4μM MG132 followed by 30 μg/μL CHX with or without 5 or 20μM of activator UP109 (also shown in figure Fig 7b), UP163, UP158 or **(B)** UP12 and analyzed by flow cytometry (n = 4 experiments each with 2 technical replicates per condition, integrated fluorescence intensity shown as box-and-whisker plots, linear mixed effects model: *P < 0.05, **P < 0.01).

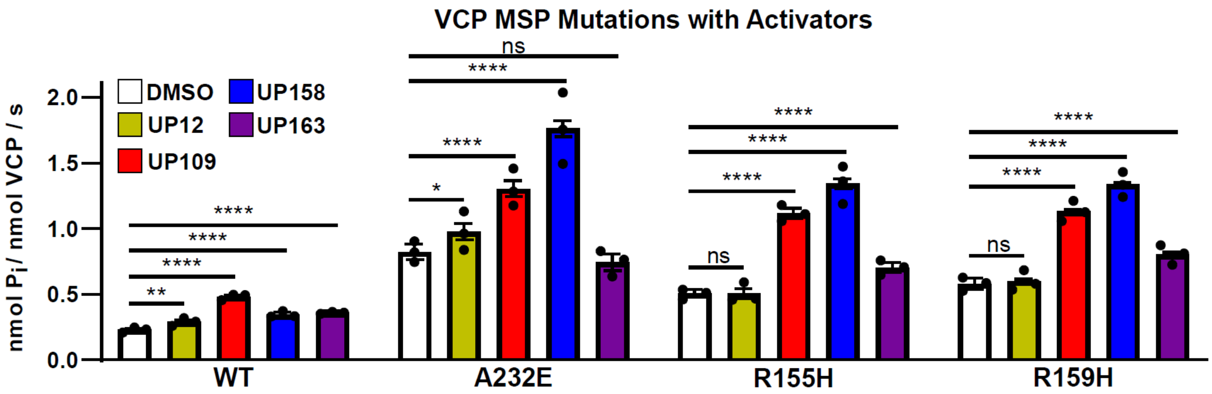

Figure S8. VCP activators variable effect on ATPase activity of MSP associated mutations in recombinant VCP. ATPase activity of 50 nM recombinant VCP protein was measured using an MESG-PNP assay upon treatment with 25 µM compound versus 2% DMSO control at 1mM ATP for WT (also shown in Fig. 5c), A232E, R155H, and R159H (n=3 with 3 technical replicated per experiment; points are expressed as beta ± SE, linear effects model: *P<0.05,**P<0.01; ***P<0.001; ****P<0.0001).

Supplemental Table 1.

A232E gRNA:

5' CACCGCCAATTGCCTTAAAGAGGGC

Primers used to amplify region off-target sequence

Off-target Sequence	Score	Gene	Chromosome	Base Pair Mismatches	Forward Primer	Reverse Primer
GCACCTGCCTTAAAGAGGGC	2.503293808		chrX	3	GCCCTCCTGCAGATTCATTG	CAGAGTTGCTTGCTTGGTGT
CCGTTTCCCTTAAAGAGGGC	1.685813175		chr3	3	CCCCACTTTTCTGCCTTTGG	CCCCTCCAAACACTGTAGCA
CAGATTGCCTGAAAGAGGGC	1.627131455		chr7	3	AAGGAAGGTGAAGGAGGAGC	TCAGTTCGCCATCTTGACCT
CCAAGTGGCTAAAAGAGGGC	1.483122363	ENSG00000166313	chr11	3	CTCAGGGTATGGGCTCTCAG	AGCTTACAGTGATGGGGCTT
CCGATGGCCTGAAAGAGGGC	0.948779615	ENSG00000261150	chr8	3	TGAGTGTCTTCATCCAGGCA	CTAAGGATGGGACCAGGGTG
CCAAATAGCTCAAAGAGGGC	0.546728365	ENSG00000171786	chr1	4	GCTCTCTCACTGAATGCTGC	ATGCAGGTTCTAAGGCCCTT

R155H gRNA

5' CACCGACATTTTTCTTGTCCGTGG

Primers used to amplify region off-target sequence

Off-target Sequence	Score	Gene	Chromosome	Base Pair Mismatches	Forward Primer	Reverse Primer
ACAATTTCCTTGTCCGTGGA	1.682165605		chr12	3	TGGCCCCATCTCTGTGAAT	TGACAGAGGCCAAGTTTTGC
TCATTTTCCTTGTCAGTGGT	0.981117534		chr18	3	AGAAGGGCCATTGGATGTCA	ACACCATTGCTCAGTAGGCT
CAAATTTTGTTGTCCGTGGT	0.934200644		chr10	4	TAGCCTCAAGTCCCAATGCA	GAGCCCAAAGTGCAGTTGAA
ACAGATTTCTTGTCAGTGGT	0.861590525		chr9	3	CCTCTCCCAGCTGTCCTTAG	ATGAAACCATGGCCCTCAGA
AAATATTCCTTGTCCGTGAT	0.634004237		chr7	4	GAAGCTGTGCAGACGGAATT	GGTCATGTGTTTGTGGCTGT
ACACTTTCCTTTTCCGTGGA	0.403810359	ENSG00000101745	chr18	4	GCAGGGATGCCAAAAGGAAA	TGGGCAGTGTTACAGGTGTA
TGATTTTTCTTGTCAGTGGA	0.274572931	ENSG00000124198	chr20	4	GGTGTCTTCTGCCCTCTCTT	AAATGGTGTCCTGGCCTGAT
ATTTTTTTCTTGTCAGTGGA	0.25657315	ENSG00000204450	chr11	4	TCTCATGGCGGCATCAGTAT	GAAACACCCCAACTTGACCC
ATTTTTTTCTTGTCAGTGGA	0.25657315	ENSG00000189253	chr11	4	AAGGTGGAGAGTTGGTGGAG	TCTCATGGCGGCATCAGTAT
AGATTTATTTTGTCAGTGGT	0.196270732	ENSG00000145495	chr5	4	CCAGACTCCTCCTTTACCCC	TCAGGAGTTCGAGACCAACC

Supplemental Table 1: gRNA used for A232E and R155H CRISPR editing and list of predicted off-target sites tested in CRISPR edited cells.

Supplemental Methods

Generation of VCP Mutant CRISPR cell lines

To generate A232E and R155H VCP mutant cell lines, CRISPR gRNAs were designed using Benchling software (http://benchling.com/) and cloned into pSpCas9(BB)-2A-Puro (PX459) V2.0 vector (plasmid #62988, gift from Feng Zhang, Addgene, Cambridge, MA, USA) (Supplemental Table 1). Homology directed repair templates were designed with PAM mutation sites. Templates were synthesized by Integrated DNA Technologies, Inc. (Coralville, IA, USA). PX489 and repair template were co-transfected into HeLa cells using Lipofectamine 3000 (Invitrogen, Waltham, MA, USA). On day two post-transfection, cells were selected in media containing 1.25µg/µL of puromycin for two days. After two days of recovery, cells were plated onto 96-well plates for clonal isolation. DNA was extracted from 96-well plates of confluent clones using QuickExtract DNA Extraction Solution (Lucigen Corporation, Middleton, WI, USA). DNA from clones were PCR amplified and screened for homology directed repair by restriction enzyme digest. CRISRP-edited cell lines were Sanger sequenced to verify knock-in of the mutations. To ensure that the guides were specific to the sites of interest, the top five potential off-target sites as well as targets that were in a gene encoding region were evaluated for cutting or homology directed repair. Primers were designed for each site, amplified with PCR, and analyzed by Sanger sequence analysis to confirm there were no off-target mutations (Supplemental Table 1).

Human induced pluripotent stem cell (iPSCs) culture

Parental KOLF 2.1J iPSCs (JIPSC1000) or KOLF 2.1J iPSCs containing CRISPR/Cas9 homozygous knock-in mutation of R159H (JIPSC1068) were purchased from The Jackson Laboratory. iPCSs were maintained at 37°C and grown in mTeSR Plus medium (100-0276, Stem Cell Technologies) on plates coated with hESC-qualified, LDEV-free matrigel (354277, Corning). mTeSR Plus medium was changed every 2 days and passaged using ReLeSR™ (5872, Stem Cell Techonologies) when cells reached 80-90% confluence.

Differentiation of human induced pluripotent stem cell culture into cortical-like neurons

To differentiate iPSCs into cortical-like neurons, piggyBac TET-ON plasmid expressing NGN2 (PB-TO-hNGN2, gift from iPSC Neurodegenerative Disease Initiative (iNDI) & Michael Ward Addgene plasmid # 172115) was integrated into parental KOLF 2.1J iPSCs or VCP-R159H iPSCs as previously described (1,2). Briefly, iPSCs were collected using accutase and washed with DPBS. Cells were plated in mTeSR Plus

medium supplemented with 50nM Chroman I (MedChem Express, HY-15392), 5uM emricasan (Selleckchem, S7775), polyamine supplement (Sigma, P8483), and 0.7µM of Trans-ISRB (Tocris, 5284) to improve viability. After 5 hours, cells were co-transfected with PB-TO-NGN2 plasmid and EF1 transposase (gift from Michael Ward) using Lipofectamine™ Stem Transfection Reagent (Thermo Fisher, STEM00008) for 24 hours. Wells were then checked for blue fluorescent protein-hNGN2 positive cells and transferred over to a new plate coated with matrigel for puromycin selection. hNGN2 positive cells were selected with 0.5µg/mL of puromycin for two days and recovered for two days. To induce expression of hNGN2, cells were collected using accutase and washed with DPBS. Cells were plated on matrigel-coated plates and grown in neuronal induction medium containing DMEM/F12, HEPES (Thermo Fisher, 11330032), N2 supplement (Gibco, 17502048), MEM non-essential amino acids (Thermo Fisher, 11140050), glutamax (Gibco, 35050-061), 2µg/mL doxycycline (MilliporeSigma, D9891) supplemented with 50nM Chroman I. Medium was switch out each day, excluding chroman I. After 3 days, predifferentiated cells were collected with accutase and replated on plates coated with 0.1mg/mL of poly-L-ornithine (MilliporeSigma, P3655) in neuronal maturation medium containing DMEM/F12, BrainPhys™ Neuronal Medium (Stemcell Technologies, 05790), N21MAX media supplement (R&D Systems, AR008), 10ng/mL recombinant human GDNF (peprotech, 450-10), 10ng/mL recombinant human BDNF (peprotech, 450-02), 10ng/mL NT-3 (peprotech, 450-03), 1ug/mL cultrex 3-D laminin matrix (R&D Systems, 344600501), 2µg/mL doxycycline, 1µM 5-fluoro-2'-deoxyuridine (MilliporeSigma, F0503), and 1µM uridine (MilliporeSigma, U3003). Half media changes were performed every three to four days. On day 10, half media changes were performed using neuronal maturation medium without DMEM/F12.

Lentiviral transduction

To express TDP-4FL into neurons, TDP-4FL lentivirus (generated by the Research Vector Core (RVC), Children's Hospital of Philadelphia) were added to neurons on day 7 of neuronal maturation at a multiplicity of infection of 3. Half media changes were performed every three to four days.

Immunohistochemistry

Immunohistochemistry was performed on 6 µm formalin-fixed, paraffin-embedded tissue sections from FTLD-TDP type D neocortex, inclusion body myopathy muscle, Paget's disease bone, normal control bone, giant cell tumor of the bone, or fracture callus tissue. After deparaffinization and rehydration of the tissue, sections were treated with methanol/H_2O_2 for 30 min then washed for 10 min. Microwave antigen

retrieval in citric acid-based antigen unmasking solution (Vector Laboratories, Burlingame, CA, USA) was then performed. Sections were washed in 0.1 M Tris buffer and blocked in 2% FBS in 0.1 M Tris buffer. Sections were then incubated with primary antibodies overnight at 4°C in a humidified chamber. Sections were again washed in 0.1 M Tris buffer, blocked in 2% FBS in 0.1 M Tris buffer, and then incubated with biotinylated species-specific secondary antibodies for 1–2 h at room temperature. Afterwards, sections were once more washed and blocked and incubated with avidin-biotin solution (Vectastain ABC kit, Vector Laboratories) for 1.5 h at room temperature. Sections were once more washed and developed using DAB (3,3′-diaminobenzidine) peroxidase substrate kit (Vector Laboratories). Finally, sections were dehydrated in an ascending ethanol series, and cleared using xylene. Mounting media (Cytoseal TM 60, Thermo Fisher) and glass coverslips were used to coverslip the slides. Primary antibodies used include rat anti-phosphoTDP-43 (1D3, TDP-43 p409/410, gifted from Drs. Maneula Neumann and Elisabeth Kremmer) and mouse anti-ubiquitin (NB300-130, Novus Biological).

Recombinant Protein Purification

N-terminal histidine tagged wild-type VCP was obtained from Addgene (plasmid #12373, gift from Axel Brunger). N-terminal histidine tagged NSF, VCP ND1L, and VCP LD2 were purchased from Genscript in pET-28a(+) vector. QuikChange II site-directed mutagenesis kit (Agilent Technologies) was used to generate VCP point mutations. Plasmid sequences were confirmed with Sanger sequencing.

For all VCP forms and NSF, Plasmids were transformed into *Eschericia coli* BL21-CodonPlus (DE3)-RIL cells (Agilent Technologies). Cells were grown in Terrific Broth with kanamycin at 37º C and induced at OD_{600} ~0.800 with 1mM IPTG for 18 hours at 18º C. Cells were collected via centrifugation at 4000xg for 15 minutes at 4º C, then resuspended in 20mM NaH_2PO_4 pH 7.4, 500mM NaCl, 10mM $MgCl_2$, 1mM ATP, 2mM β-mercaptoethanol, 20mM imidazole, 1mg/mL lysozyme, EDTA-free Complete Protease inhibitor (Roche) at 4º C followed by sonication. Lysates were cleared via centrifugation at 27,500xg for 30 min at 4º C. The supernatant was filtered, then applied to a 5mL HisTrap™ excel nickel column (Cytiva, Marlborough, MA, USA). The column was washed with 10 column volumes of 20mM NaH_2PO_4 pH 7.4, 500mM NaCl, 10mM $MgCl_2$, 1mM ATP, 2mM β-mercaptoethanol, 20mM imidazole, EDTA-free Complete Protease inhibitor (Roche). Proteins were eluted using a 5mL linear gradient of 20mM NaH_2PO_4 pH 7.4, 500mM NaCl, 10mM $MgCl_2$, 20mM imidazole to 20mM NaH_2PO_4 pH 7.4, 500mM NaCl, 10mM $MgCl_2$, 500mM imidazole followed by 50mL of the 500mM imidazole buffer. Elute was concentrated using an Amicon Ultra-15 centrifugal unit

(Millipore) then loaded onto a Superdex 200 Increase 10/300 GL column (Cytiva). 0.5mL fractions were collected in 50mM HEPES pH 7.4, 150mM KCl, 2mM $MgCl_2$, 5% glycerol. Protein concentration was determined using the Peirce™ BCA protein assay (Thermo Fisher). Fractions were concentrated to 5mg/mL using an Amicon Ultra – 0.5mL centrifugal unit (Millipore), flash frozen, and stored in liquid nitrogen. Expression plasmid was isolated from each protein preparation and confirmed by Sanger sequencing. Each preparations purity was assessed by gel electrophoresis.

UFD1 and NPLOC4 were purified by MD Anderson Cancer Center Core for Biomolecular Structure and Function similarly via the following protocol. NPLOC4 untagged plasmid and His-UFD1 were expressed separately in *E. coli* (DE3) cells grown in terrific broth at 37º C until ~OD_{600} of 1.0, then were induced with 0.4mM IPTG overnight at 16º C. Cells were harvested by centrifugation, then re-suspended in 50mM Tris pH 7.4, 500mM KCl, 5mM $MgCl_2$, 20mM imidazole, 5% glycerol, and 2mM β-mercaptoethanol with complete protease inhibitors. The resuspension pellets were mixed 1:1 Ufd1:Nploc4 and lysed by sonication. The lysate was centrifuged for 1 hour to remove insoluble material. The Ufd1-Nploc4 complex was purified from lysate using Ni-NTA resin in 50mM Tris pH 8.0, 500mM KCl, 5mM $MgCl_2$, 20mM imidazole, 5% glycerol, and 2mM β-mercaptoethanol. Resin was washed using the same buffer, then eluted using 300mM imidazole. The protein elute was applied to an S75 16/16 column (Cytiva) with buffer 20mM HEPES pH 7.4, 250mM KCl, 1mM $MgCl_2$, 5% glycerol, and 0.5mM TCEP. Concentration of UFD1-NPLOC4 was estimated as 1mg/mL, flash frozen, and stored at -80º C. Purity was assessed via gel electrophoresis and dynamic light scattering.

Supplemental References

1. Flores EL et al. iNDI Transcription Factor-NGN2 differentiation of human iPSC into cortical neurons Version 2. Published online June 14, 2023. Accessed March 19, 2024. https://www.protocols.io/view/indi-transcription-factor-ngn2-differentiation-of-cvpxw5pn

2. Cookson M et al. iNDI PiggyBac-TO-hNGN2 transfection protocol Version 1. Published online August 8, 2023. Accessed March 19, 2024. https://www.protocols.io/view/indi-piggybac-to-hngn2-transfection-protocol-versi-cyd6xs9e
