# Supplementary material for: VCP activator reverses nuclear proteostasis defects and enhances TDP-43 aggregate clearance in multisystem proteinopathy models
Source: J Clin Invest. 2024 May 23;134(14):e169039. doi: 10.1172/JCI169039 (PMC11257039; doi:10.1172/JCI169039)
Supplement: Unedited blot and gel images [file jci-134-169039-s011.pdf]

Uncropped/Unedited Western blots (Revisions)

JCI Manuscript Submission

Figure 1

Figure 1, Panel F

Total and Nuclear Lysate
Antibody: Mouse anti VCP (Invitrogen, MA3-004)

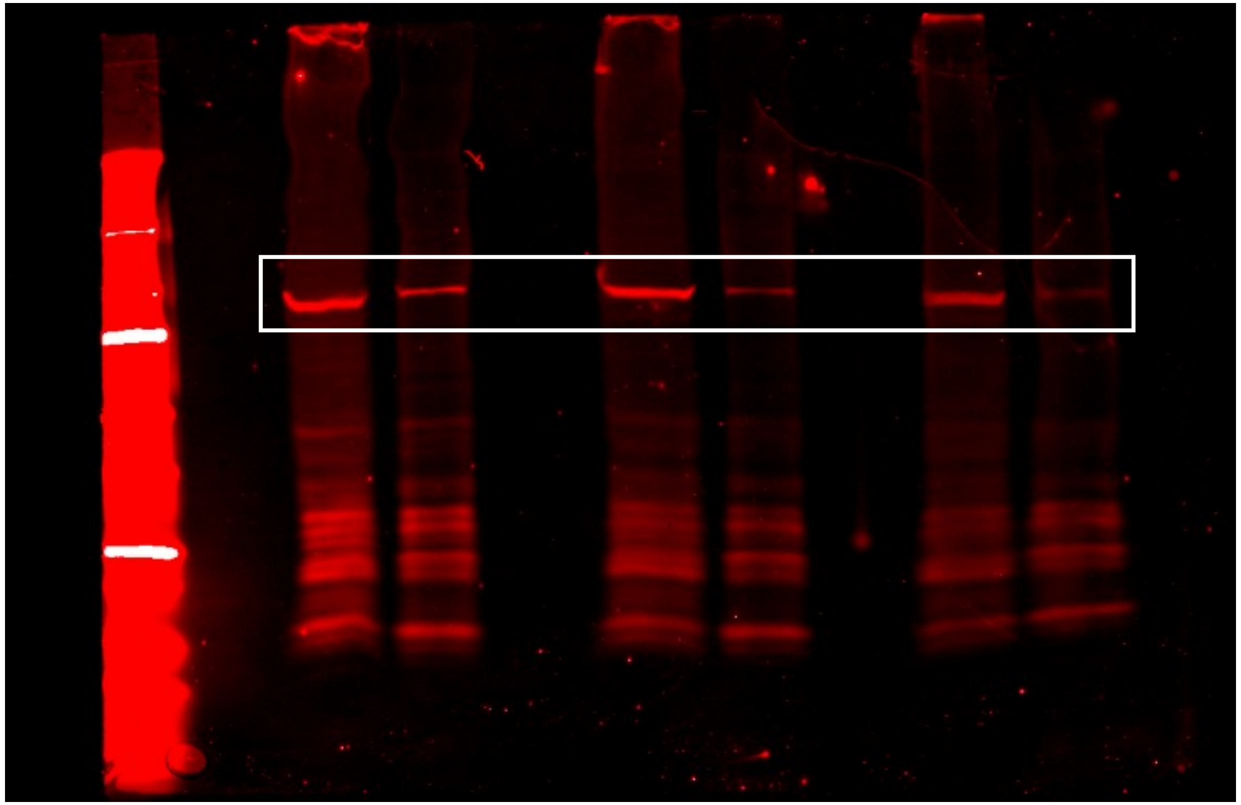

Cropped sections used for figures denoted by white box.

Figure 1, Panel F

Total and Nuclear Lysate
Antibody: Rat anti Nup98 (Abcam, ab50610)

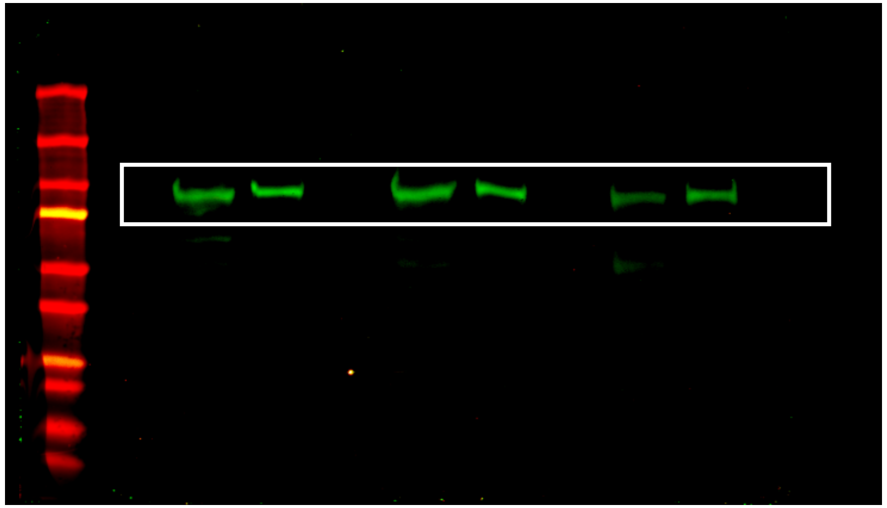

Total and Nuclear Lysate
Antibody: Rabbit anti HSP90 (Cell Signaling, 4874)

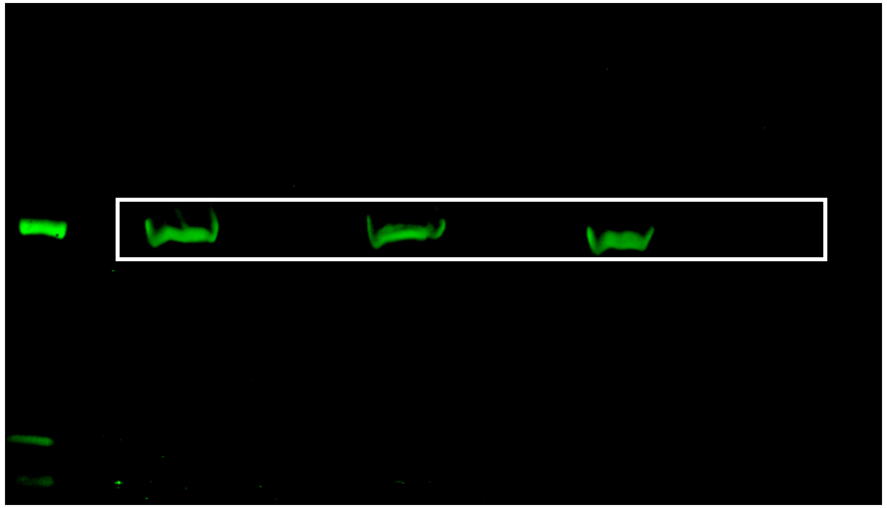

Same blot as above (not shown in figures)
Antibody: Mouse anti VCP (Invitrogen, MA3-004)

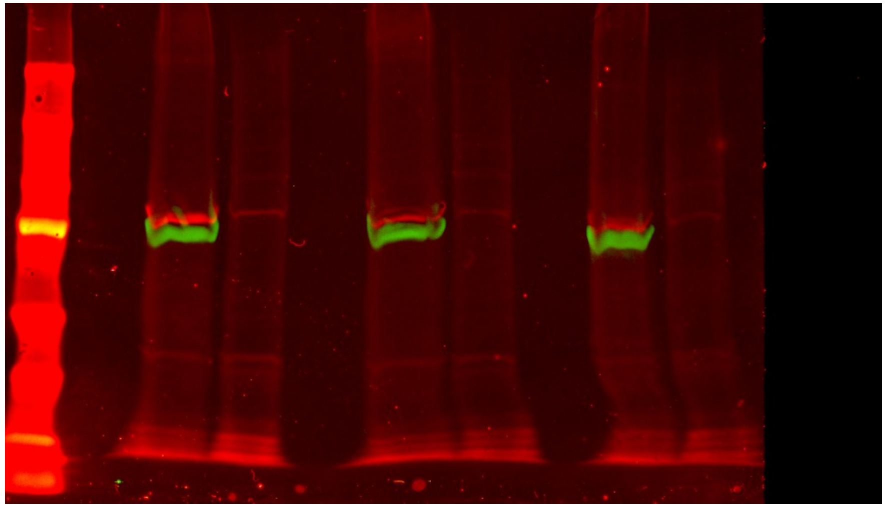

Cropped sections used for figures denoted by white box.

Figure 2

Figure 2, Panel C

Soluble TDP-4FL
Antibody: Mouse anti Myc (9E10)

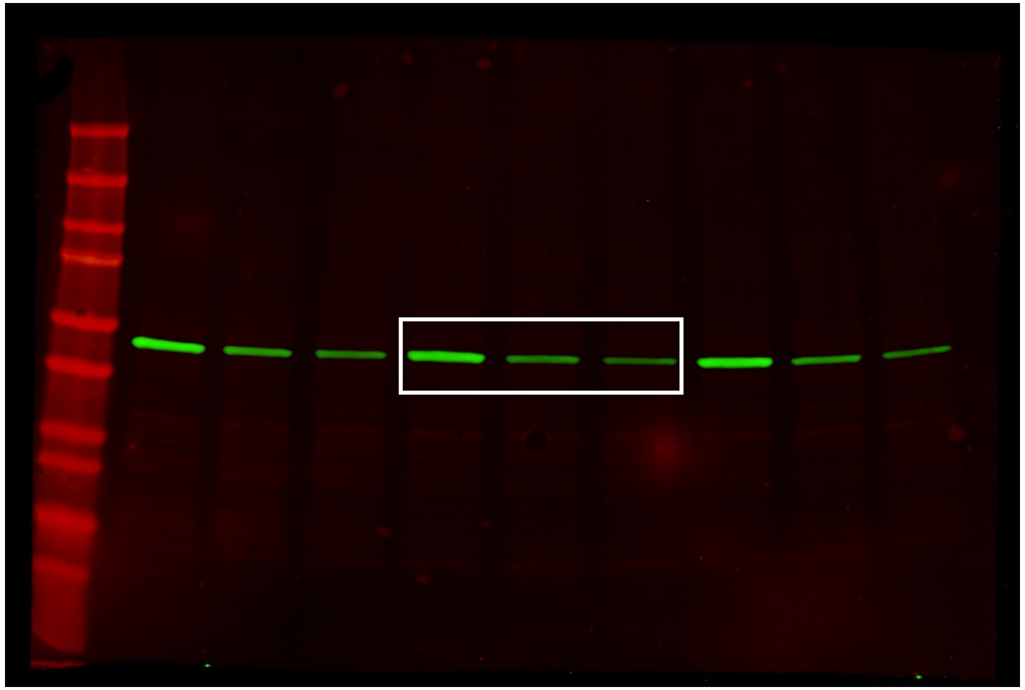

Insoluble TDP-4FL
Antibody: Mouse anti Myc (9E10)

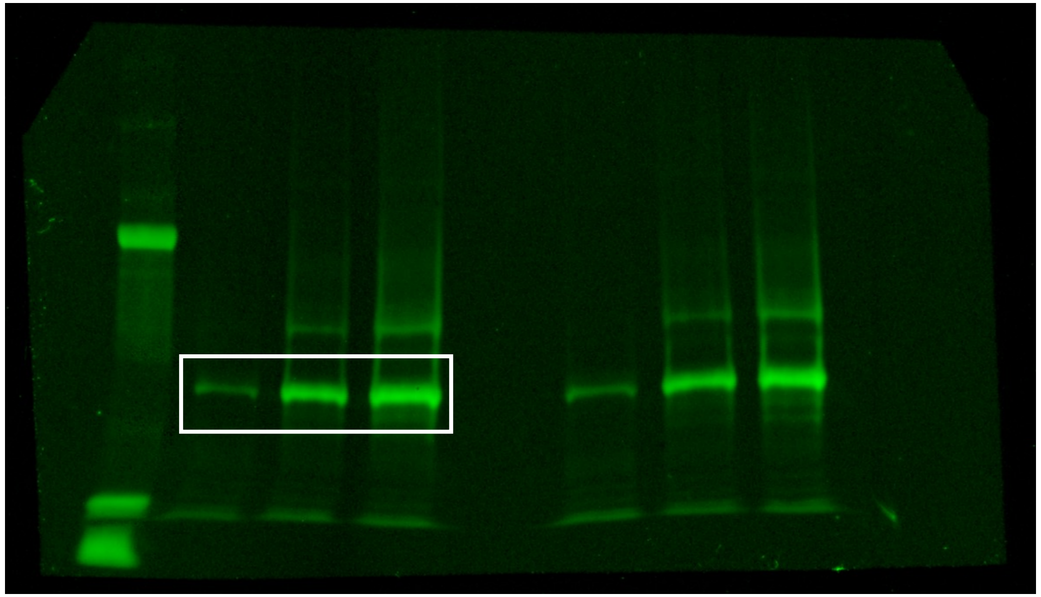

Cropped sections used for figures denoted by white box.

Figure 2, Panel C

Loading Control
Antibody: Rabbit anti GAPDH (Cell Signaling, 2118)

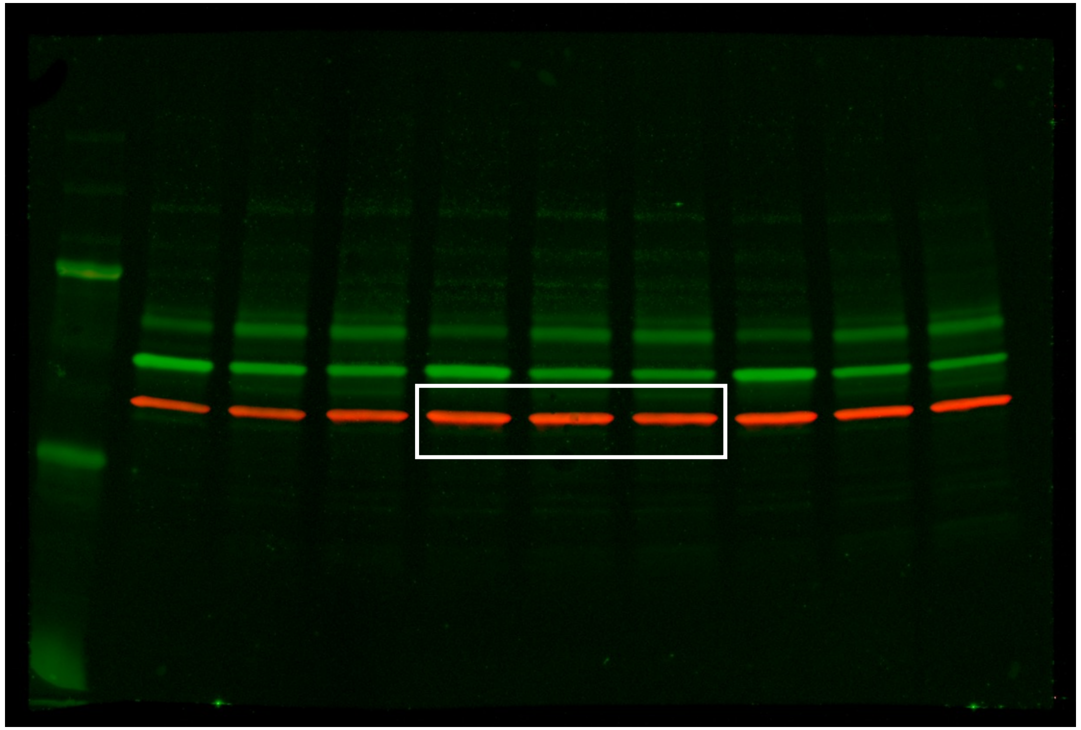

Cropped sections used for figures denoted by white box.

Figure 2, Panel F

Myc Immunoprecipitation

Antibody: Rabbit anti Ubiquitin (Cell Signaling, 43124); Mouse anti Myc (9E10)

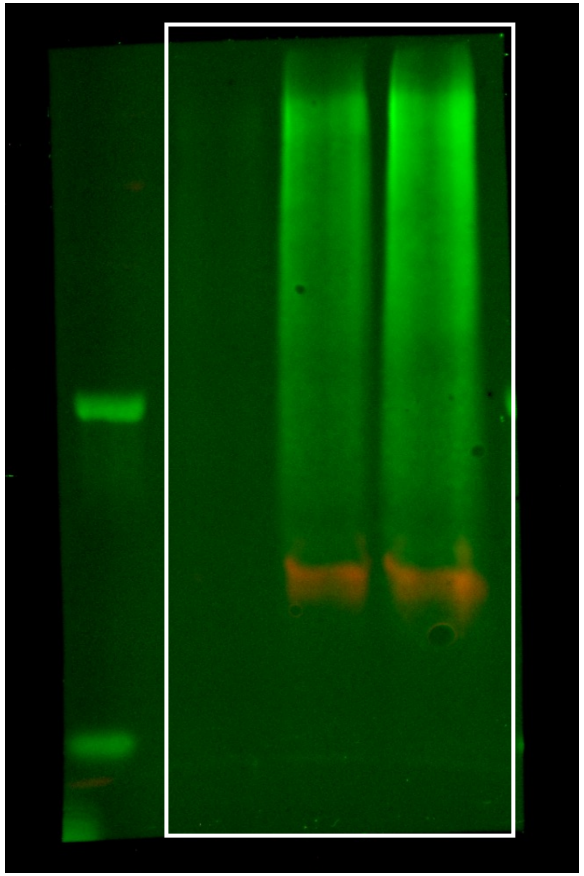

Figure 3

Figure 3, Panel F

Soluble TDP-4FL
Antibody: Mouse anti Myc (9E10)

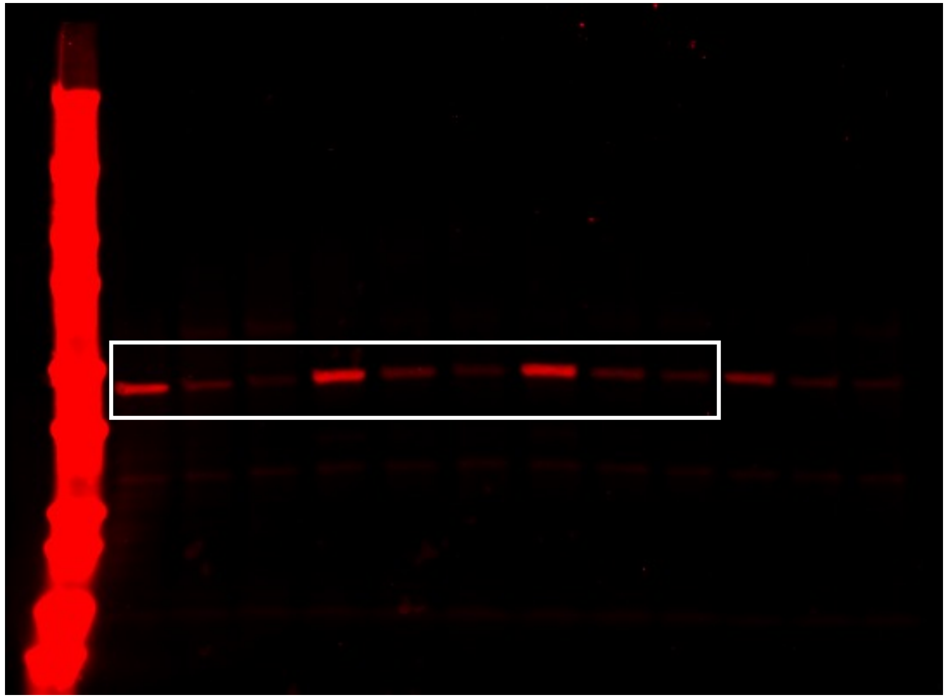

Insoluble TDP-4FL
Antibody: Mouse anti Myc (9E10)

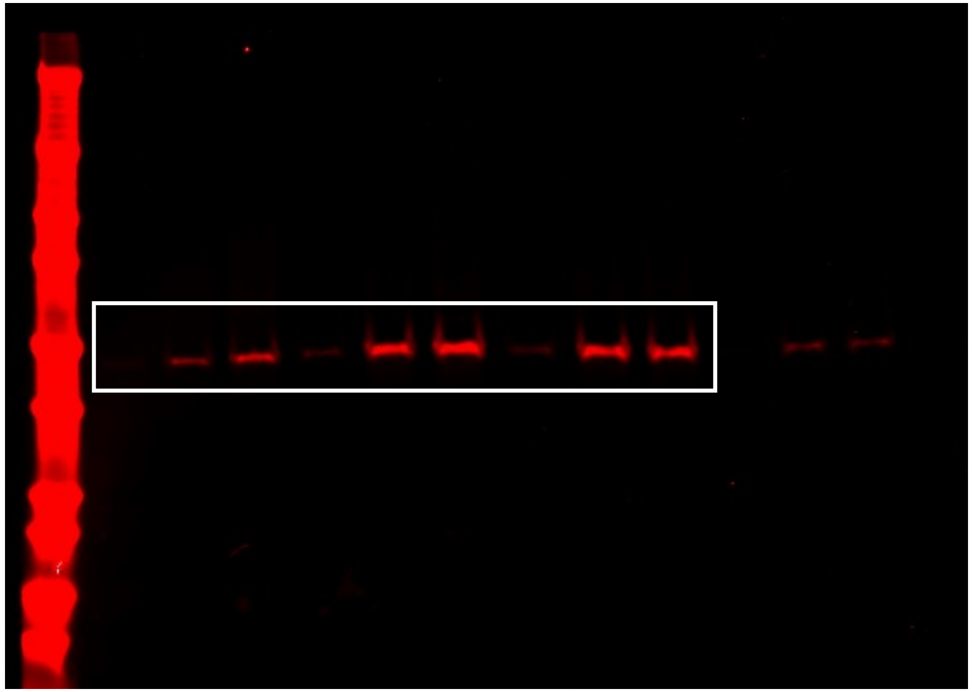

Cropped sections used for figures denoted by white box.

Figure 3, Panel F

Loading Control
Antibody: Rabbit anti GAPDH (Cell Signaling, 2118)

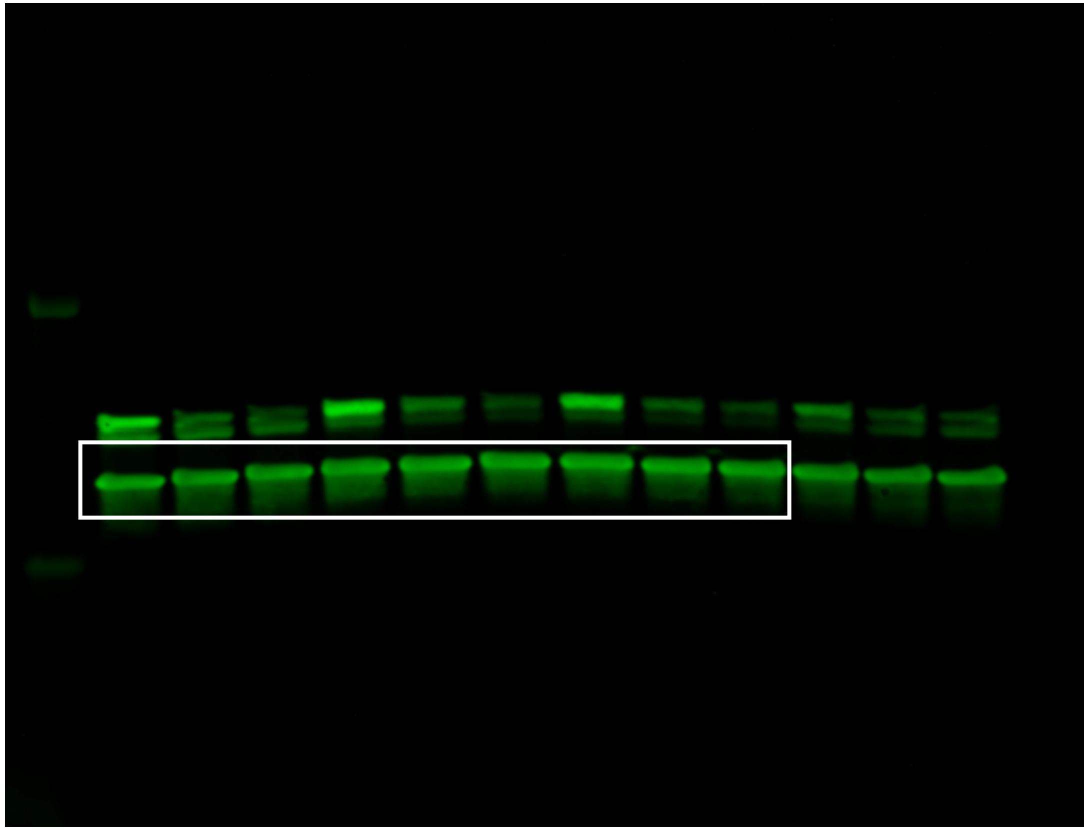

Cropped sections used for figures denoted by white box.

Figure 4

Figure 4, Panel C

Soluble TDP-4FL
Antibody: Mouse anti Myc (9E10)

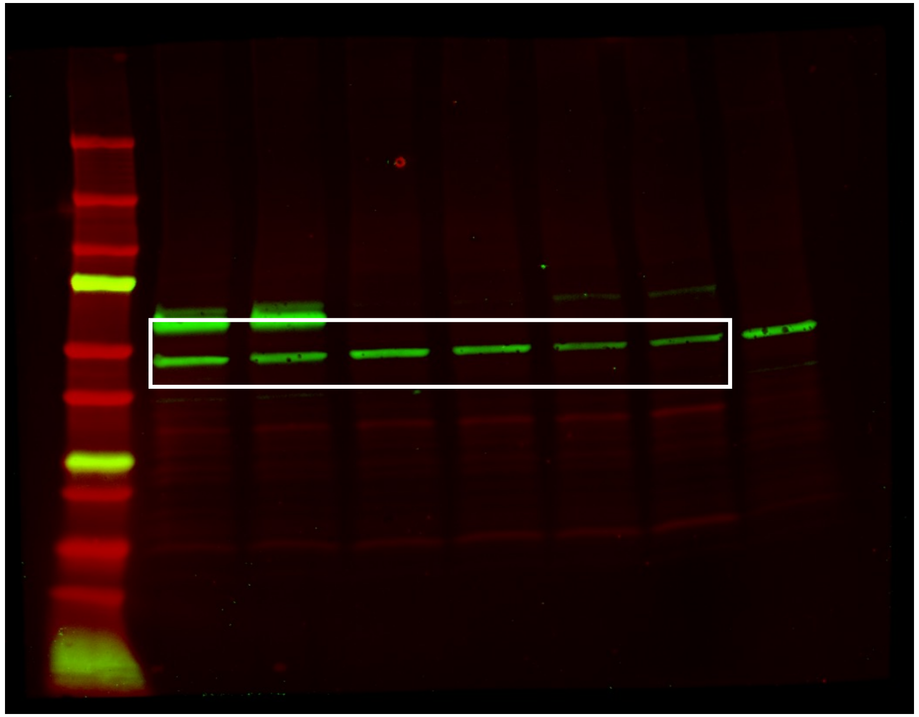

Insoluble TDP-4FL
Antibody: Mouse anti Myc (9E10)

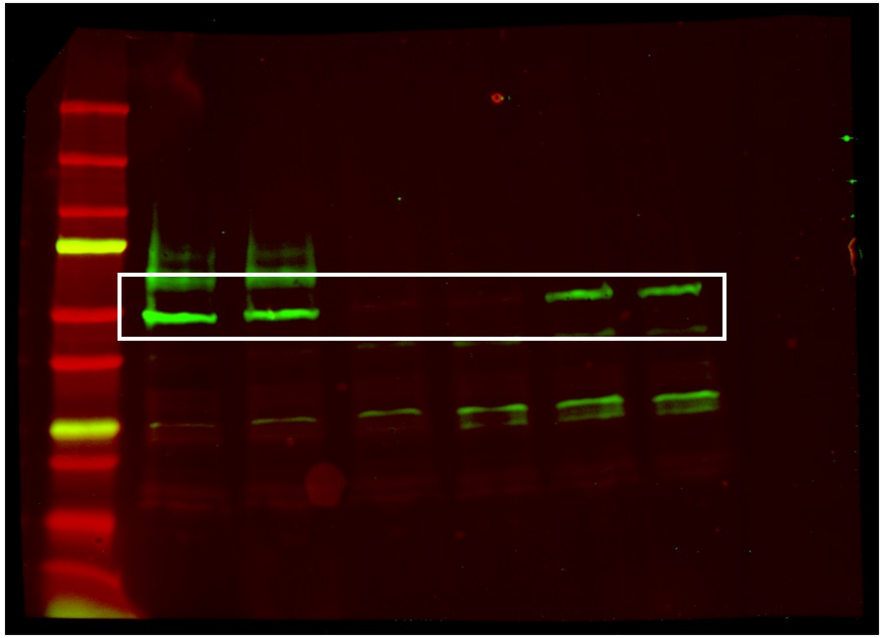

Cropped sections used for figures denoted by white box.

Figure 4, Panel C

Loading Control
Antibody: Rabbit anti GAPDH (Cell Signaling, 2118)

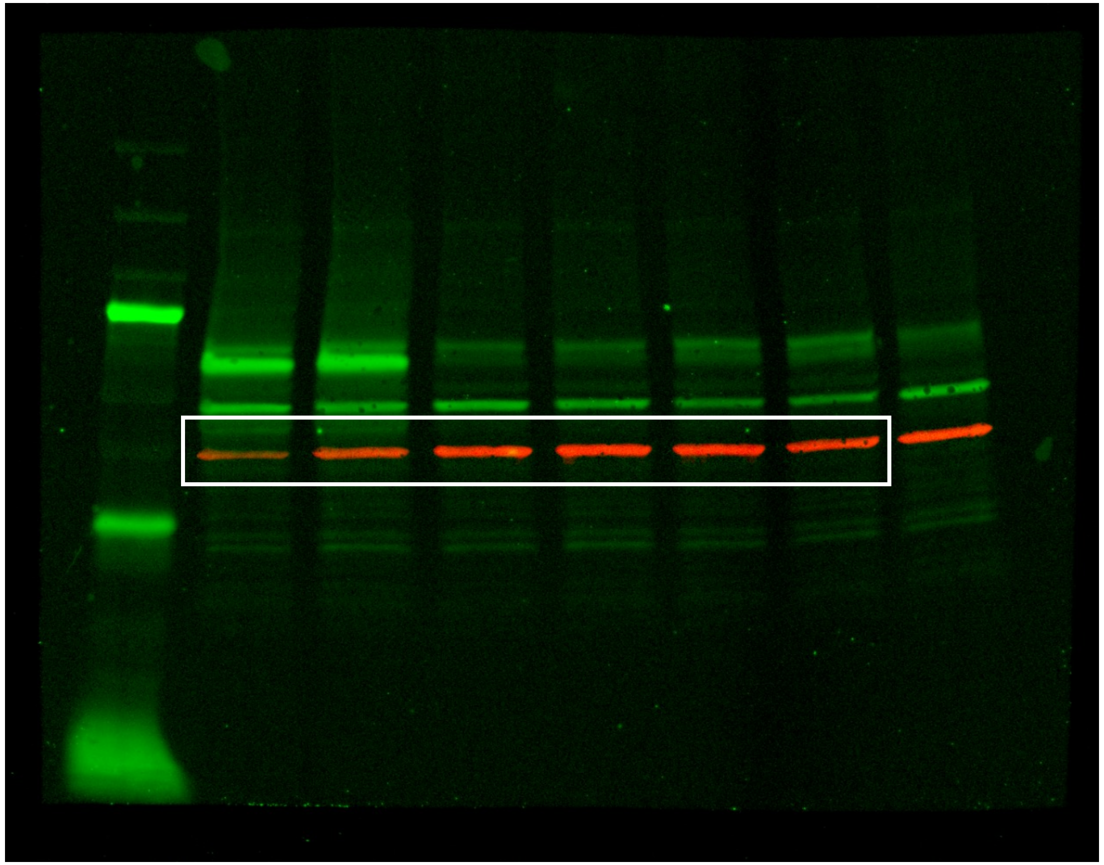

Cropped sections used for figures denoted by white box.

Figure 7

Figure 7, Panel E

Soluble TDP-4FL
Antibody: Mouse anti Myc (9E10)

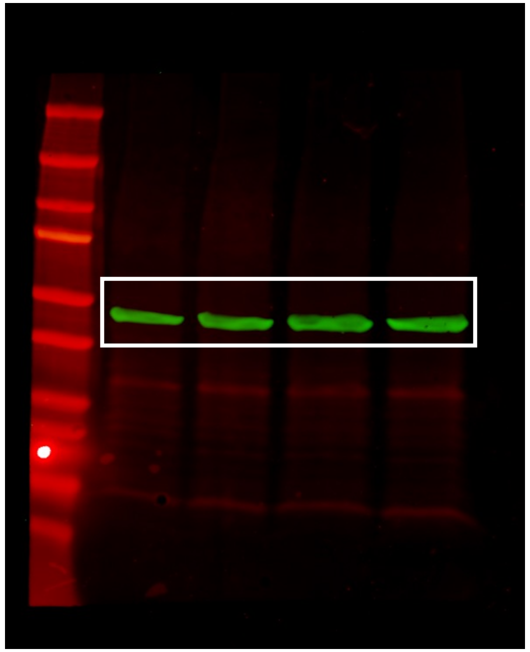

Insoluble TDP-4FL
Antibody: Mouse anti Myc (9E10)

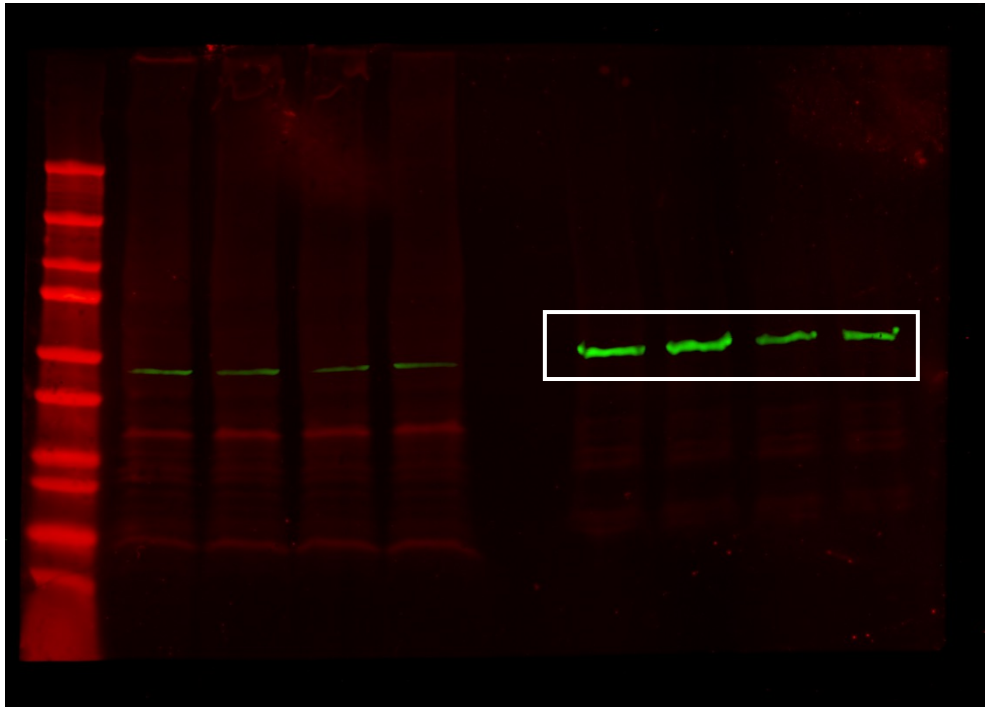

Cropped sections used for figures denoted by white box.

Figure 7, Panel E

Loading Control
Antibody: Rabbit anti GAPDH (Cell Signaling, 2118)

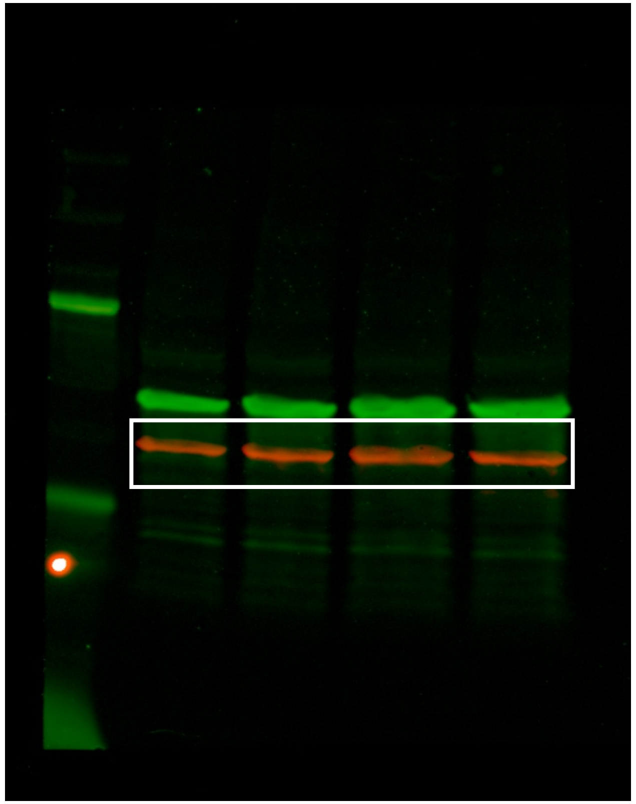

Cropped sections used for figures denoted by white box.

Figure 7, Panel J (VCP-A232E)

Soluble TDP-4FL
Antibody: Mouse anti Myc (9E10)

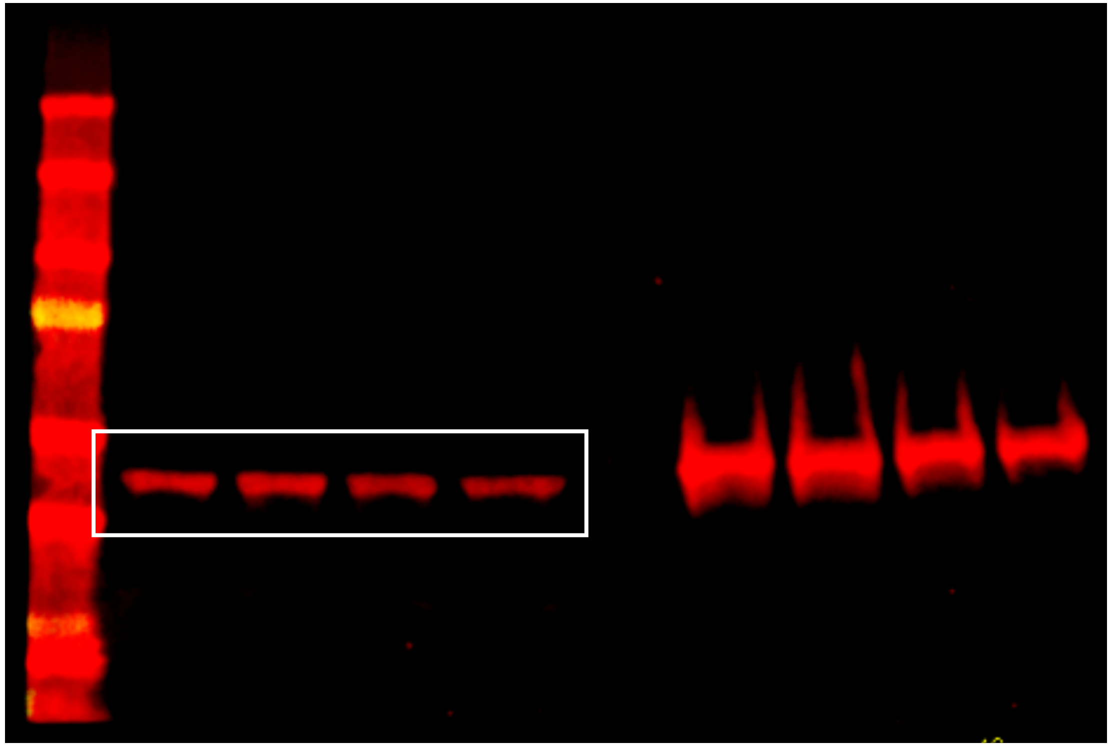

GAPDH Loading Control (Green) & Insoluble TDP-4FL (Red)
Antibodies: 1) Green: Rabbit anti GAPDH (Cell Signaling, 2118)
2) Red: Mouse anti Myc (9E10)

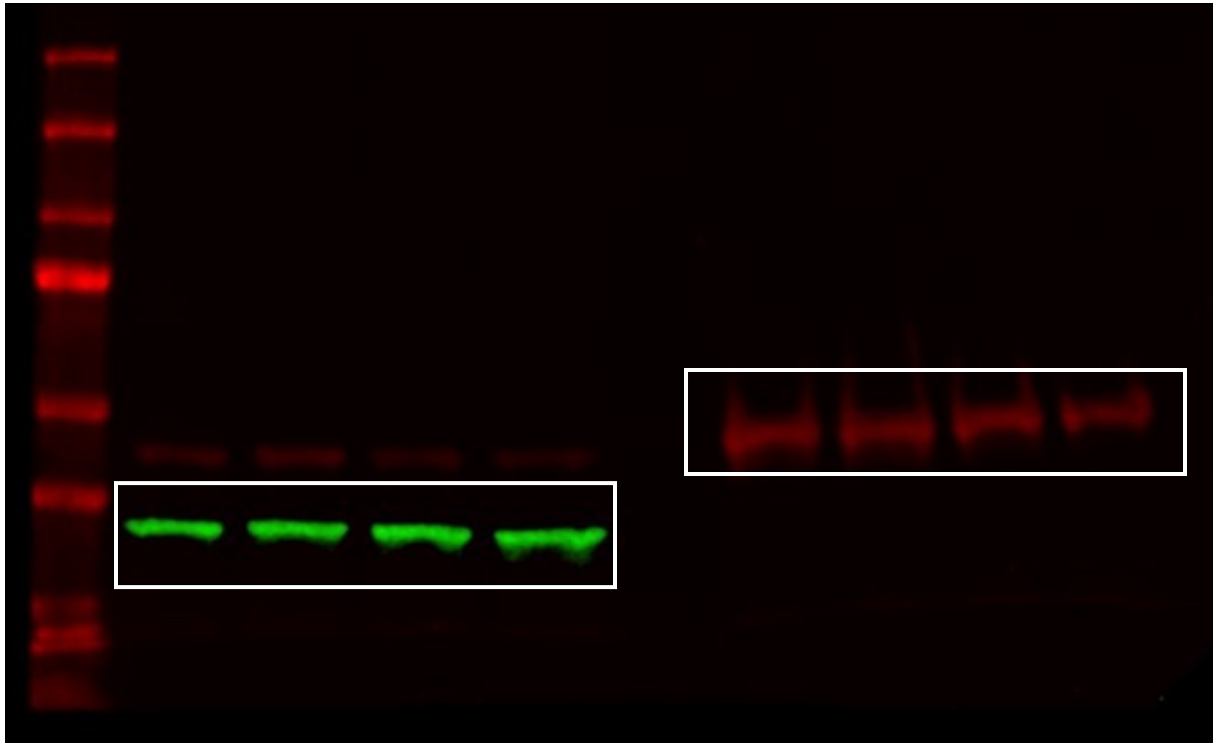

Both blots are the same; Left blot is higher exposure
Cropped sections used for figures denoted by white box.

Figure 7, Panel J (VCP-R155H)

Soluble TDP-4FL
Antibody: Mouse anti Myc (9E10)

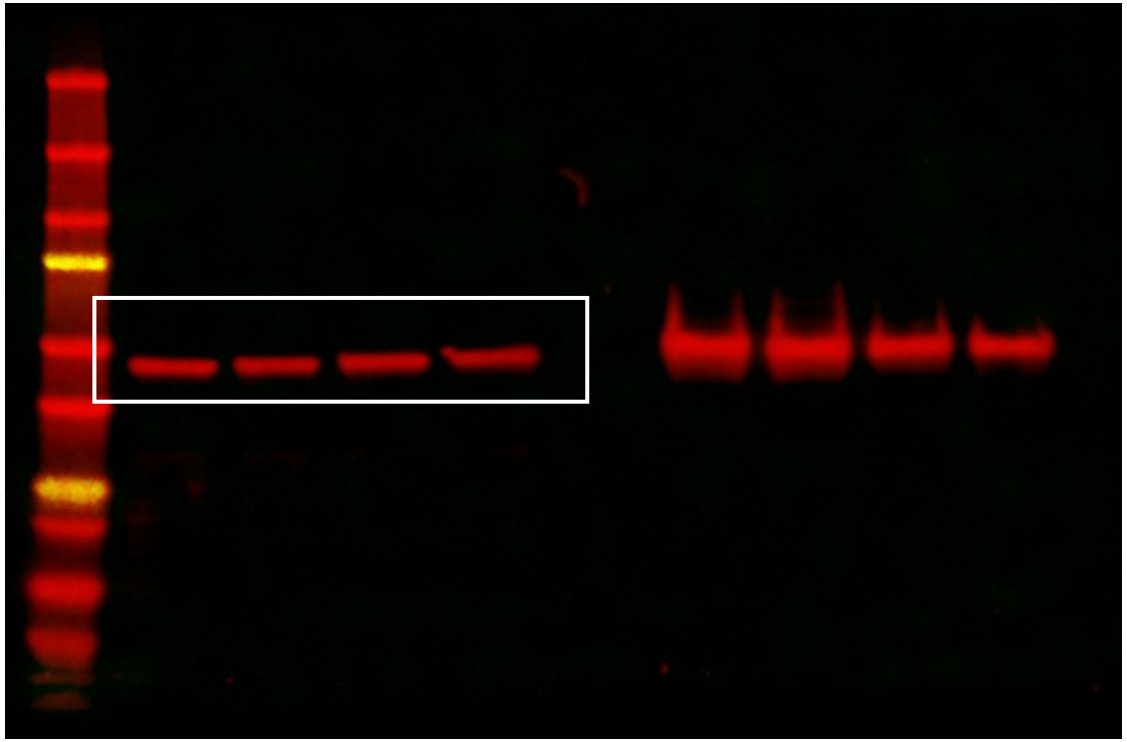

GAPDH Loading Control (Green) & Insoluble TDP-4FL (Red)
Antibodies: 1) Green: Rabbit anti GAPDH (Cell Signaling, 2118)
2) Red: Mouse anti Myc (9E10)

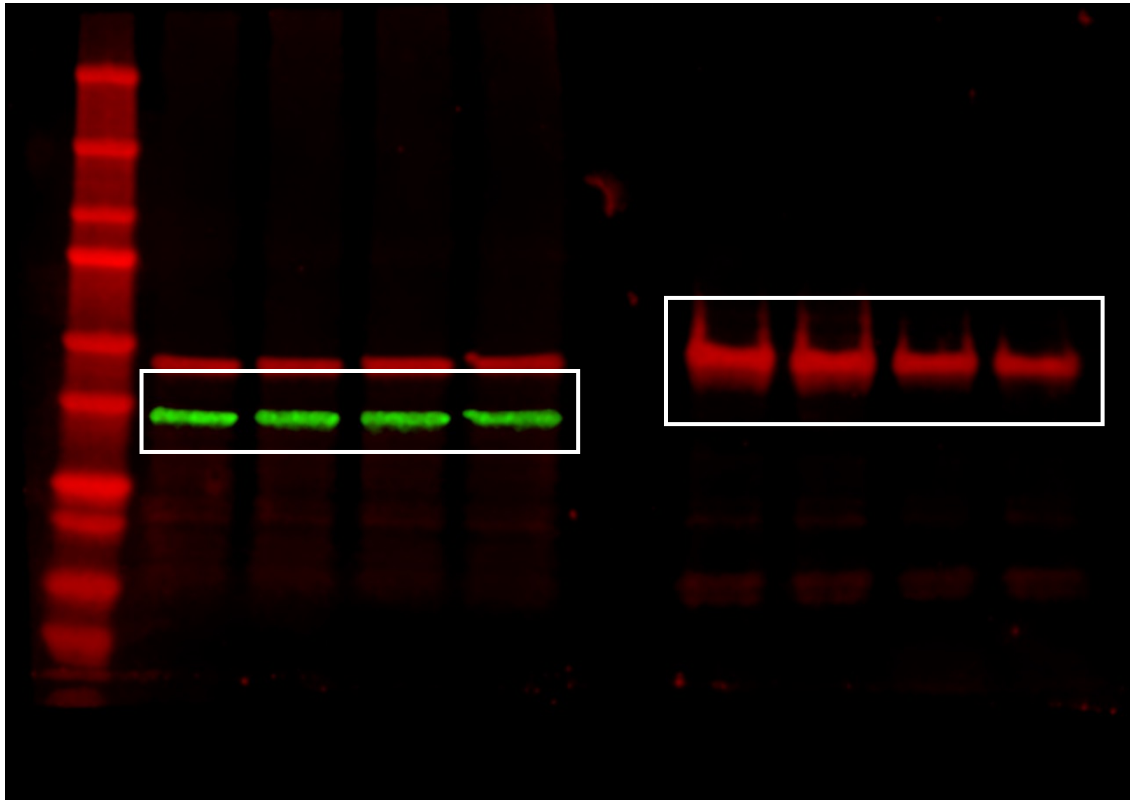

Both blots are the same; Left blot is higher exposure
Cropped sections used for figures denoted by white box.

Figure 8

Figure 8, Panel D (Parental cells)

Insoluble TDP-4FL
Antibody: Mouse anti Myc (9E10)

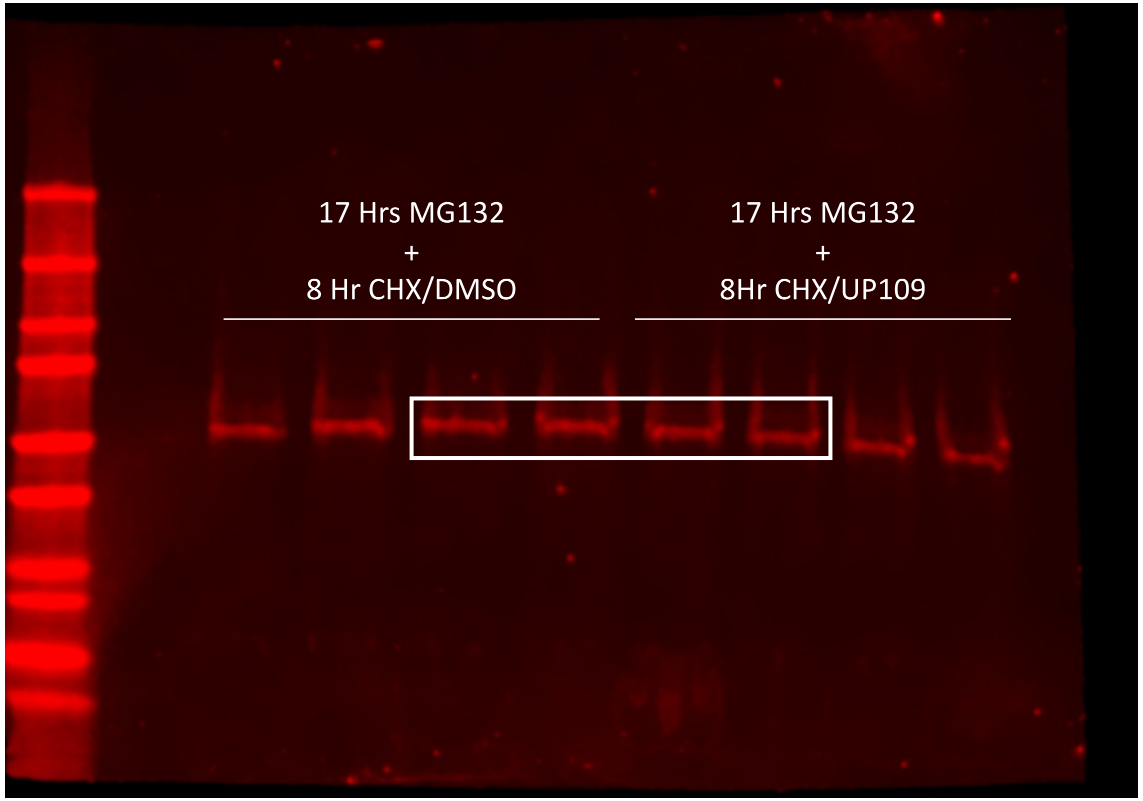

GAPDH Loading Control (Green) & Soluble TDP-4FL (Red)
Antibodies: 1) Green: Rabbit anti GAPDH (Cell Signaling, 2118)
2) Red: Mouse anti Myc (9E10)

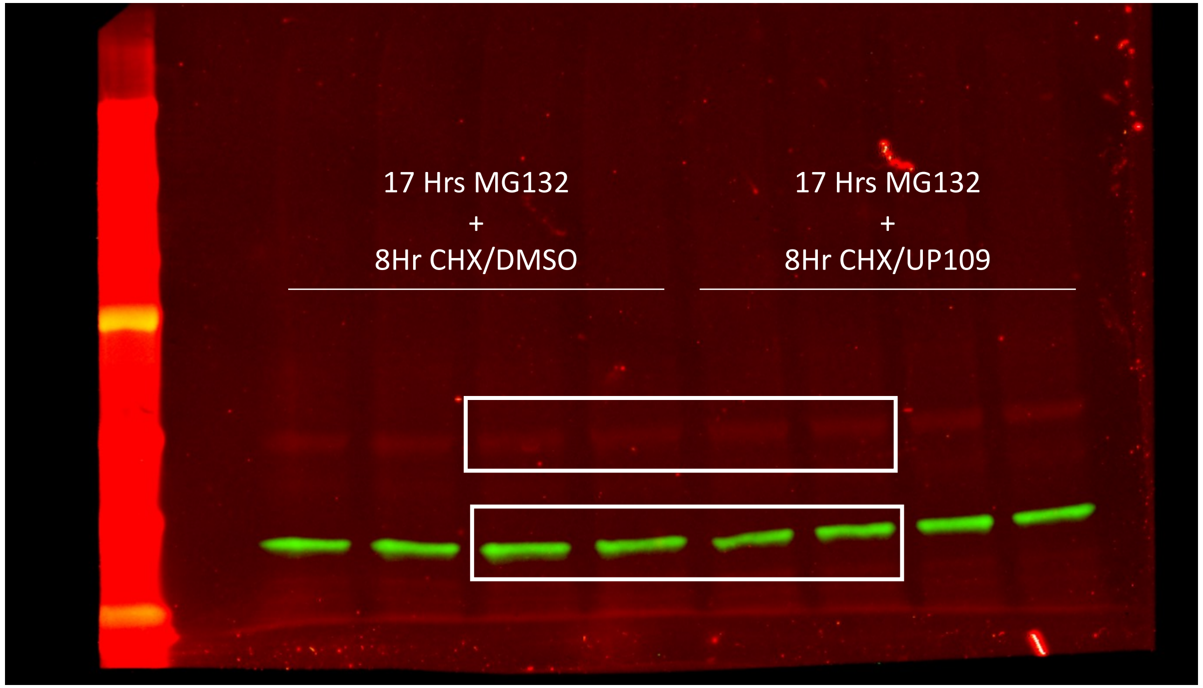

Cropped sections used for figures denoted by white box.

Figure 8, Panel E (VCP-R159H)

Insoluble TDP-4FL
Antibody: Mouse anti Myc (9E10)

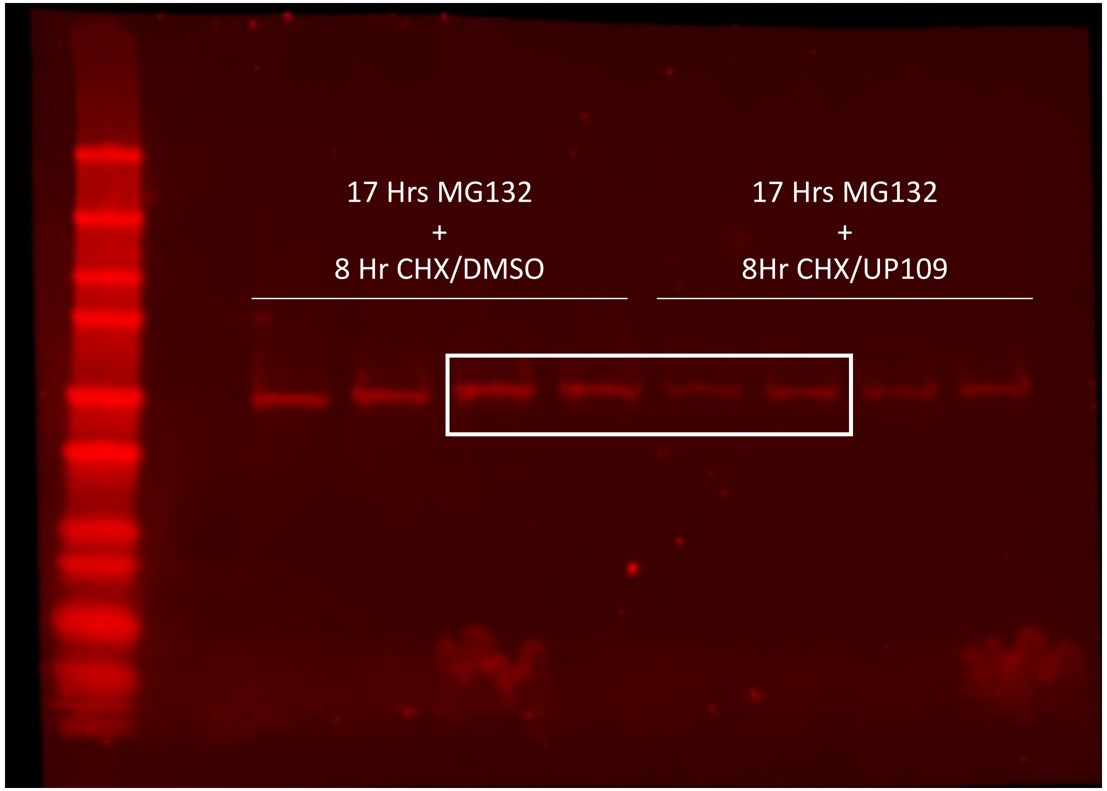

GAPDH Loading Control (Green) & Soluble TDP-4FL (Red)
Antibodies: 1) Green: Rabbit anti GAPDH (Cell Signaling, 2118)
2) Red: Mouse anti Myc (9E10)

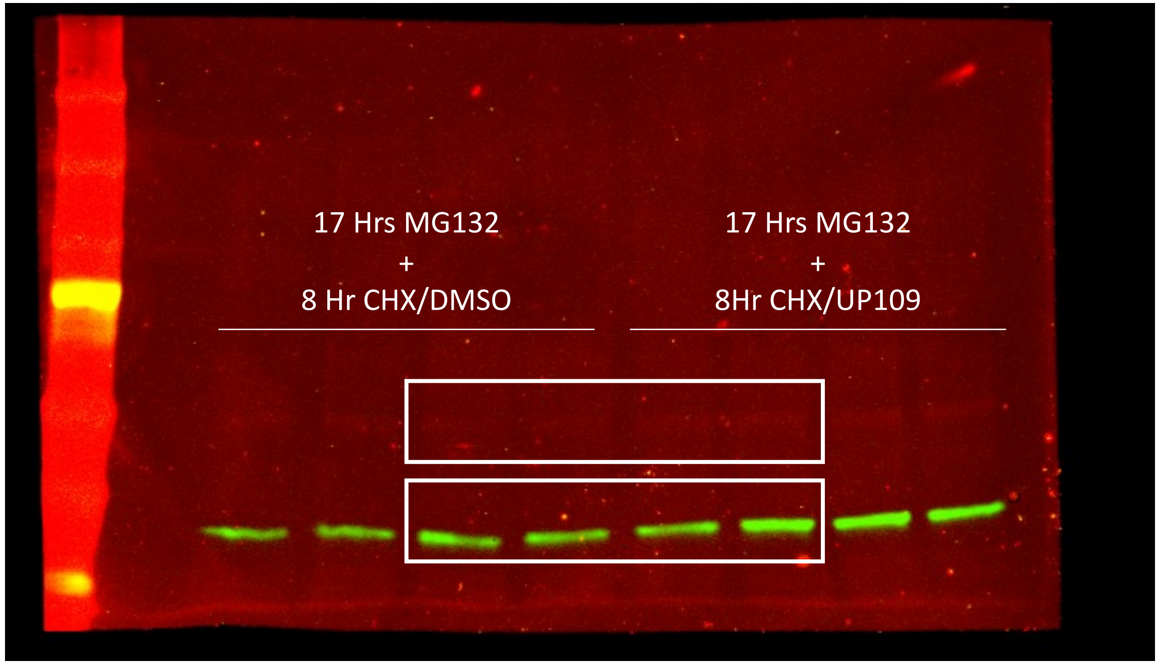

Cropped sections used for figures denoted by white box.

Supplemental Figure 1

Supplemental Figure 1, Panel B

Soluble TDP-4FL
Rabbit anti Ubiquitin (Cell Signaling, 43124)

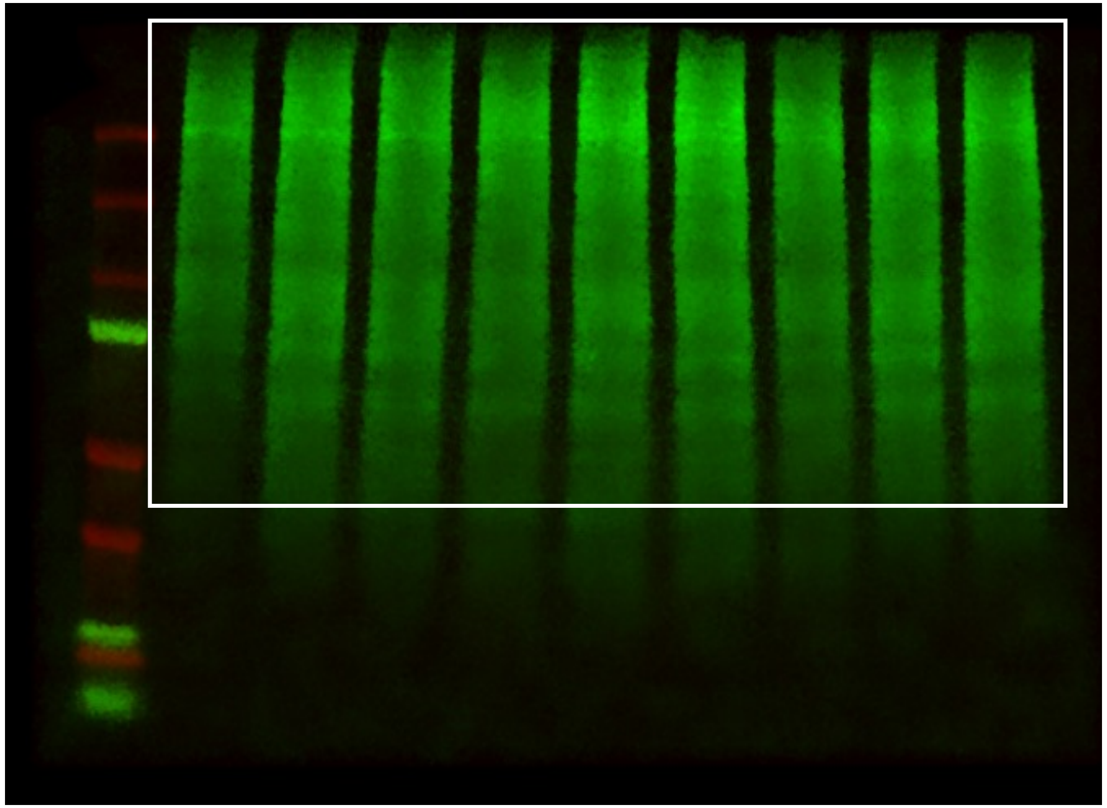

Insoluble TDP-4FL
Rabbit anti Ubiquitin (Cell Signaling, 43124)

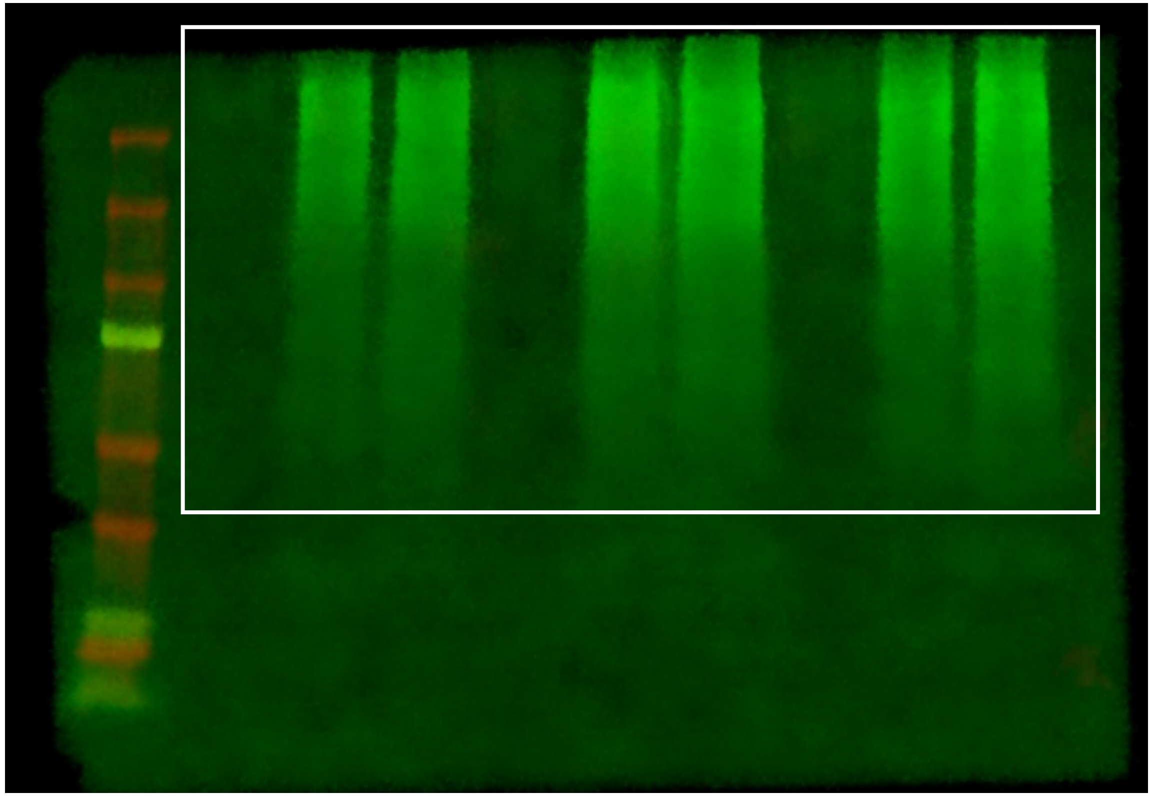

Cropped sections used for figures denoted by white box.

Supplemental Figure 1, Panel B

Loading Control
Antibody: Rabbit anti GAPDH (Cell Signaling, 2118)

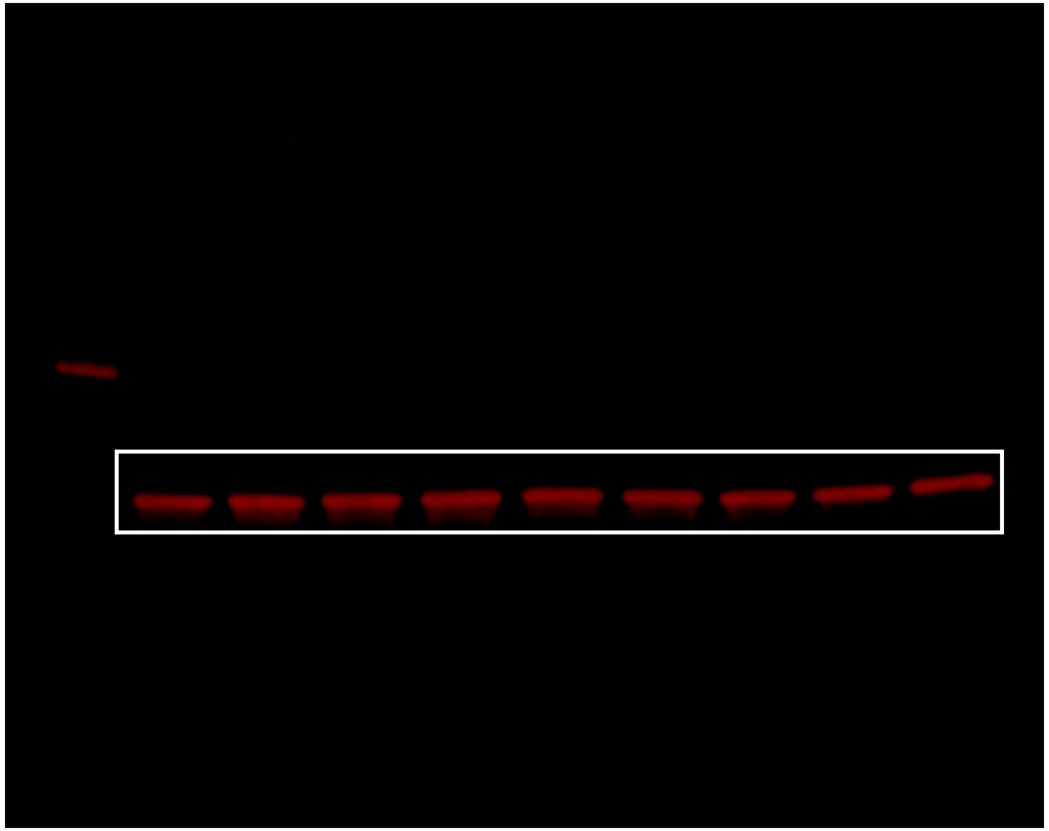

Cropped sections used for figures denoted by white box.

Supplemental Figure 2

Supplemental Figure 2, Panel B

Soluble TDP-4FL
Antibody: Mouse anti Myc (9E10)

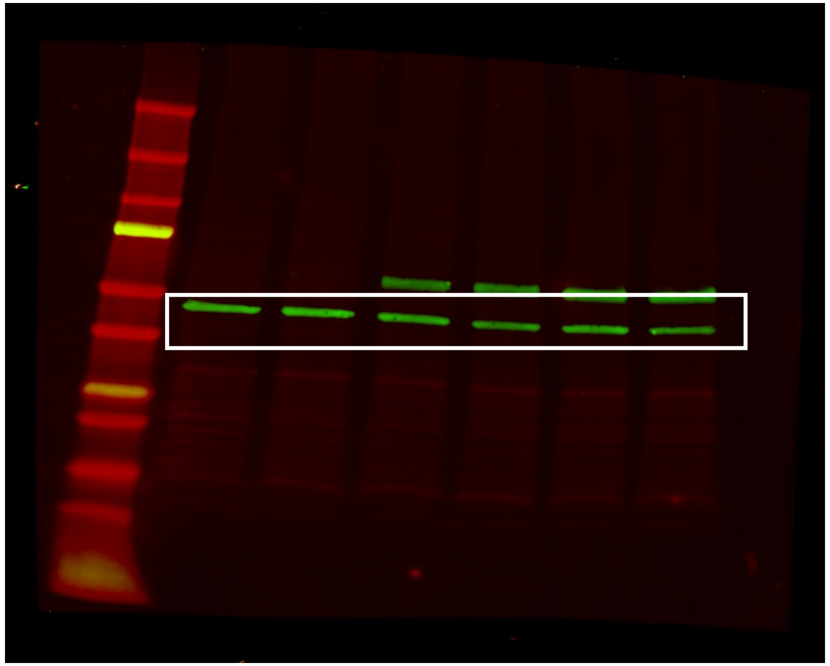

Insoluble TDP-4FL
Antibody: Mouse anti Myc (9E10)

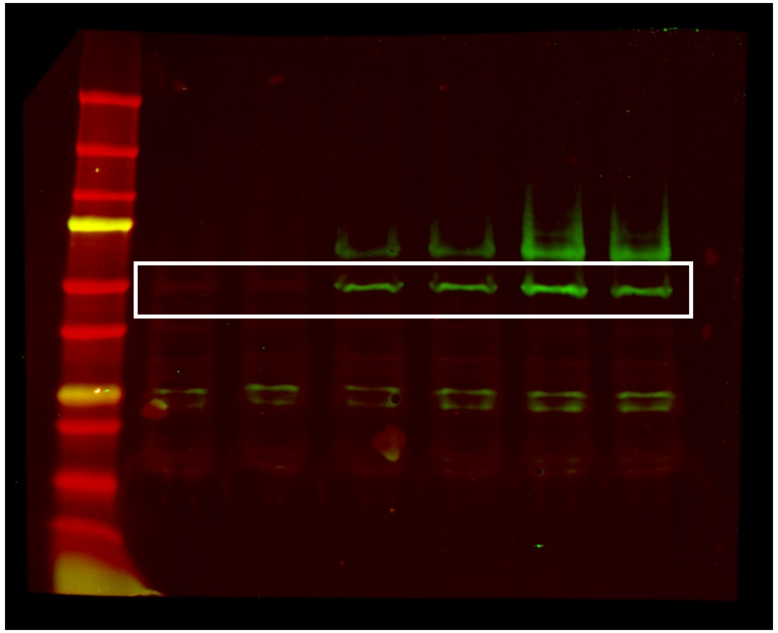

Cropped sections used for figures denoted by white box.

Supplemental Figure 2, Panel B

Loading Control
Antibody: Rabbit anti GAPDH (Cell Signaling, 2118)

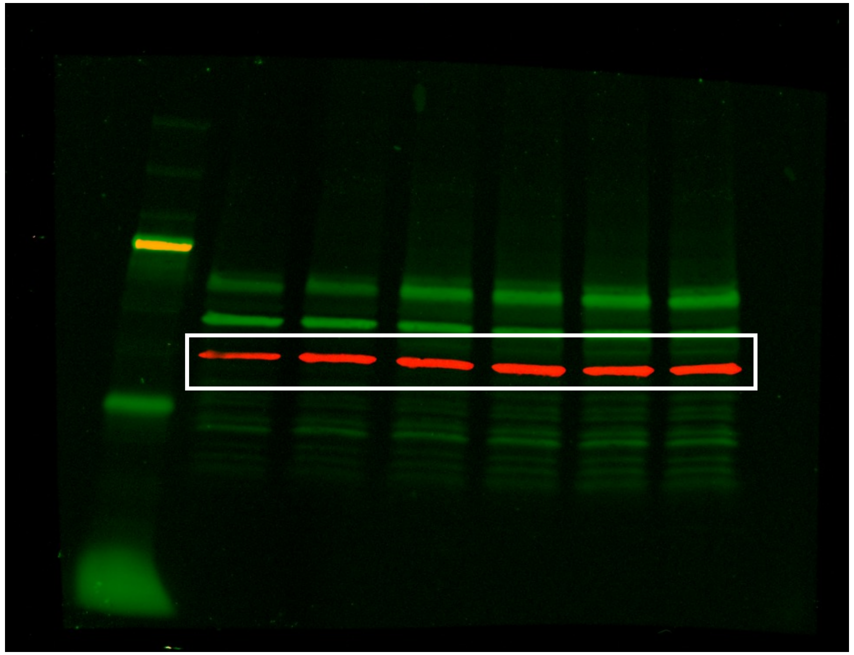

Cropped sections used for figures denoted by white box.

Supplemental Figure 5

Supplemental Figure 5, Panel A

Soluble TDP-4FL
Rabbit anti C-terminal TDP (C2089, Gift from CNDR)

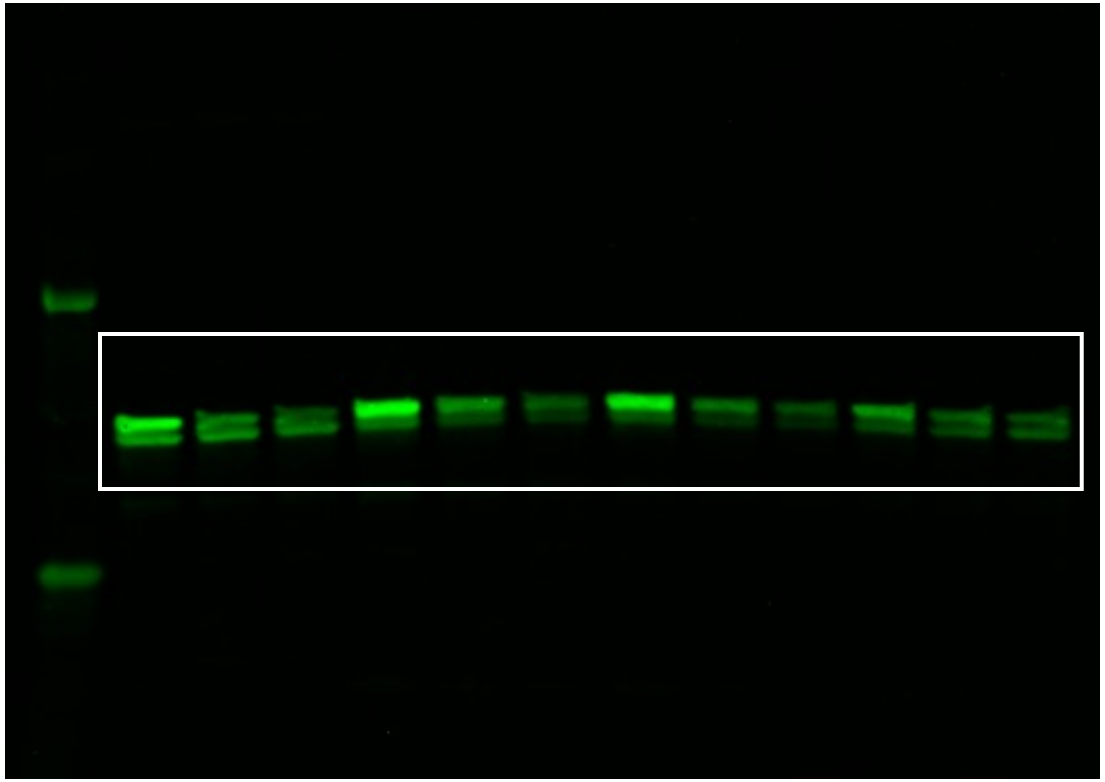

Insoluble TDP-4FL
Rabbit anti C-terminal TDP (C2089, Gift from CNDR)

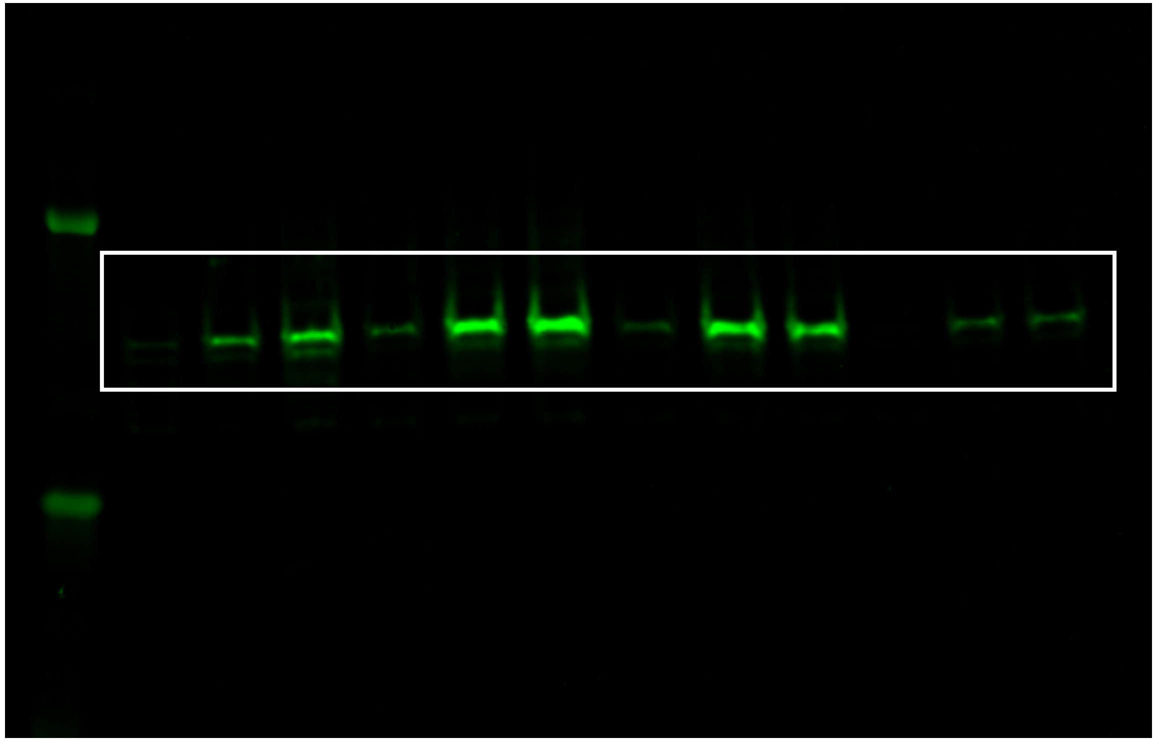

Cropped sections used for figures denoted by white box.
